# A practical synthesis of enantiopure *syn*-β-amino-α-hydroxy acids from α-amino acids with application in the formal syntheses of l-TFB-TBOA and (S)-vigabatrin

**DOI:** 10.1039/d5ra05586e

**Published:** 2025-09-26

**Authors:** Bohua Long, Peng Zhang, Mengmeng Jiang, Pengfei Guo, Xuanluan Chen, Shunlei Shang, Zhengzhi Wu

**Affiliations:** a The First Affiliated Hospital of Shenzhen University, Shenzhen Second People's Hospital Shenzhen 518035 China bhlong121@163.com szwzz001@163.com; b Shenzhen Institute of Geriatric Medicine Shenzhen 518035 China; c Department of Nephrology, China-Japan Friendship Hospital Beijing 100029 China 18810568600@163.com

## Abstract

Enantiopure *syn*-β-amino-α-hydroxy acids have been synthesized from α-amino acids in a multi-step procedure that exhibits a high level of stereoselectivity and good overall yields. A stepwise oxidation of the terminal olefin to a carboxylic acid delivered an essentially quantitative yield *via* a cleaner process relative to the conventional one-pot oxidation. The practical value of this transformation has been demonstrated in the formal synthesis of l-TFB-TBOA and (S)-vigabatrin.

## Abstract 2

Enantiopure *syn*-β-amino-α-hydroxy acids have been synthesized from α-amino acids in a multi-step procedure that exhibits a high level of stereoselectivity and good overall yields.

## Graphical abstract

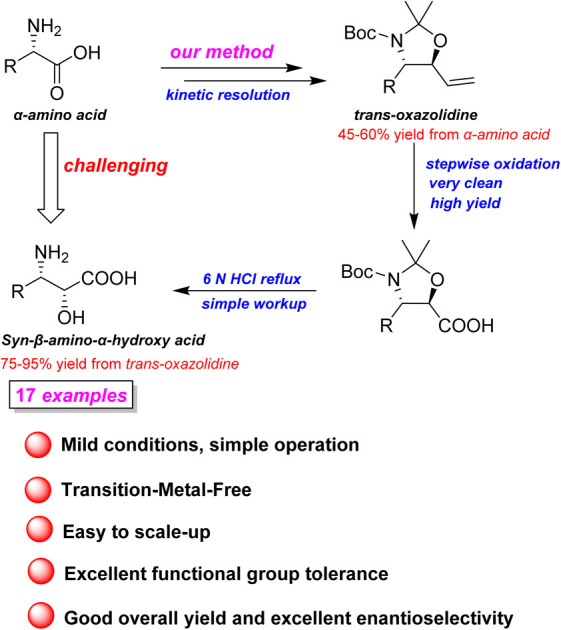

*Syn*-β-amino-α-hydroxy acid fragments are present in numerous biologically active molecules, drugs and natural products, exhibiting diverse biological activities. For example, Taxol and Docetaxel have significant activity in breast, non-small-cell lung, ovarian and head and neck cancers.^1^ The analogs, ABT-271 and BMS-275183, showed superior activities compared to Taxol in tumor cell line cytotoxicity assays and *in vivo* tests.^2^ Moreover, natural active peptides, such as Probestin,^3^ Microginin,^4^ Amastatin,^5^ Rakicidin A^6^ and KRI1314 (ref. 7) have demonstrated significant therapeutic potential as protease inhibitors, a new hypoxia-selective cytotoxin and an orally active renin inhibitor (Scheme 1).

**Scheme 1:**
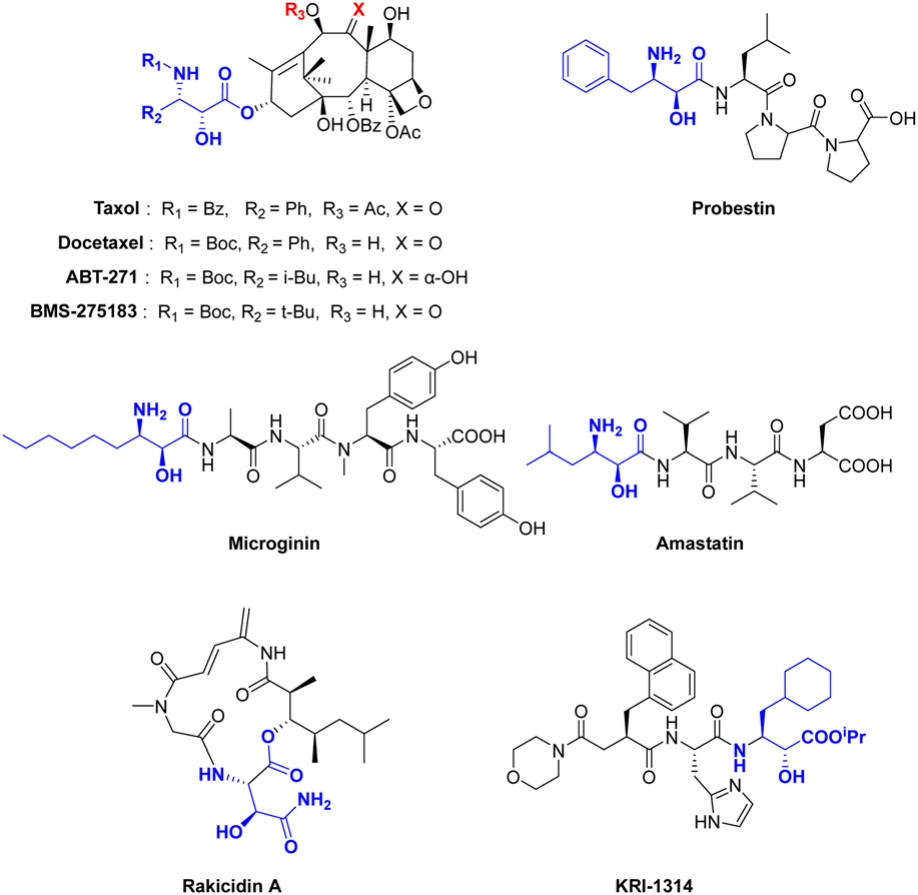
Representative bioactive molecules containing the *syn*-β-amino-α- hydroxy acid moiety.

Oxidation of the corresponding α-hydroxy amide generates the α-keto amide in high yield^8,9^ (Scheme 2). The α-ketoamide moiety is found in many drugs and natural products, such as the HCV NS3/4 A protease inhibitor Telaprevir,^10^ potent protease inhibitors cyclotheonamides A-B^11,12^ and cyclotheonellazoles A–C.^13^ The α-ketoamide is a peculiarly reactive ambident proelectrophile and pronucleophile moiety. It has been widely utilized by medicinal chemists to develop compounds with favorable biological activities, low toxicity, and promising pharmokinetic (PK) and drug-like properties with respect to highly complex biological targets.^14^

**Scheme 2:**
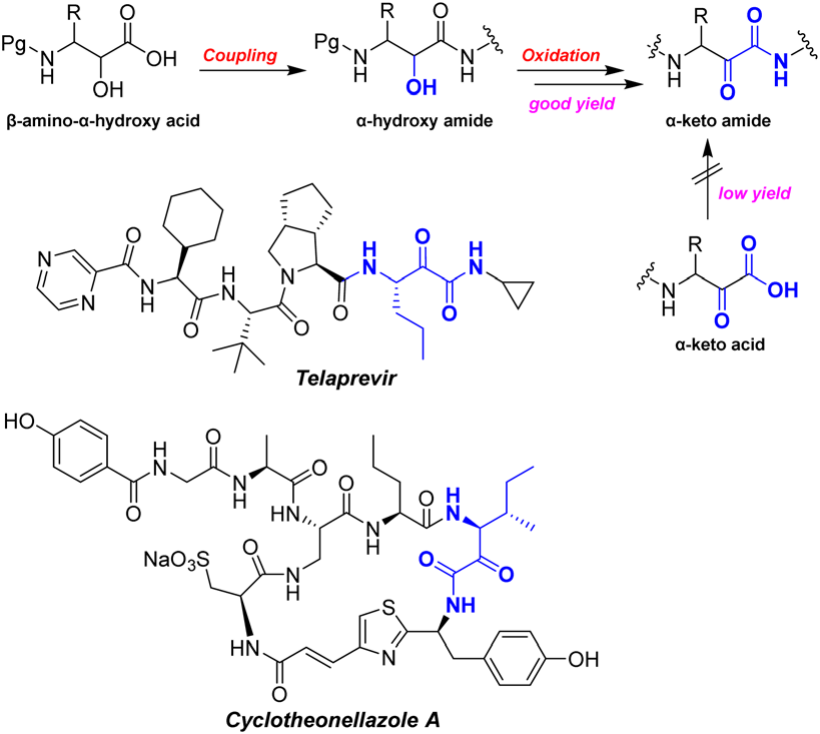
Synthesis of α-keto amide from β-amino-α-hydroxy acid.

All the *syn*-β-amino-α-hydroxy acid are not readily accessible in an enantiomerically pure form. A method that is capable of rapidly and efficiently producing *syn*-β-amino-α-hydroxy acid with high stereoselectivity warrants comprehensive exploratory work.

Current methods face several limitations. The most widely used procedure based on the conversion of natural α-amino acids is illustrated in Scheme 3. The protected aldehydes are treated with hazardous NaCN^15^ or KCN^16^ or ACH^9,17^ to give cyanohydrin intermediates in essentially quantitative yields, with subsequent heating under reflux in aqueous HCl to obtain a *ca.* 1:1 mixture of *syn*- and *anti*-diastereomers.

**Scheme 3:**
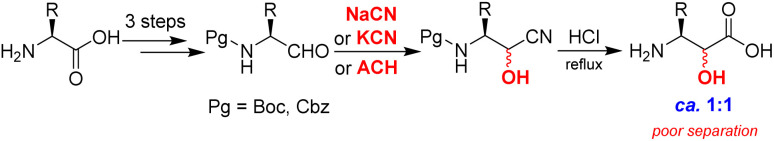
Cyanation reaction for the synthesis of *syn*-β-amino-α-hydroxy acid.

Chiral reagent-controlled synthetic methods, such as selective opening of a chiral epoxide^18^ and asymmetric aminohydroxyation,^19^ have been employed as shown in Scheme 4. However, when applied to the alkyl acrylate, these methods deliver poor regioselectivity and stereoselectivity.^19^

**Scheme 4:**
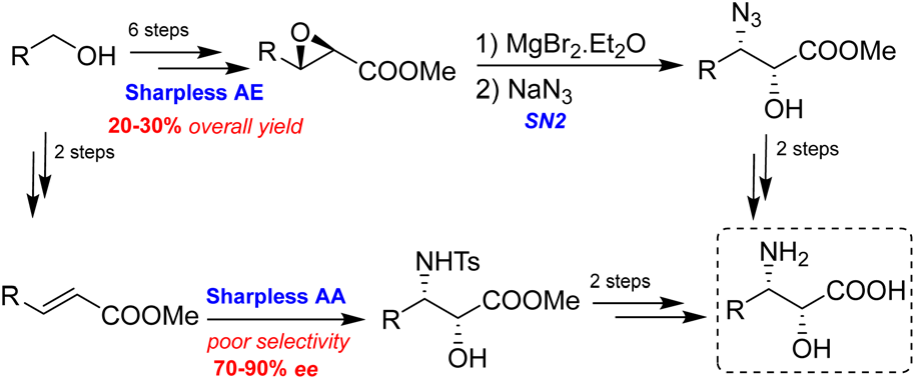
Sharpless reaction for the synthesis of *syn*-β-amino-α-hydroxy acid.

Naturally occurring α-amino acids have been widely used as chiral building blocks in organic synthesis. As shown in Scheme 5, l-norvaline 1 was selected as the starting material, which was subjected to esterification, Boc protection, and LiAlH_4_ reduction. The target α-amino alcohol 2 was oxidized to the corresponding α-amino aldehyde 3*via* 2-iodoxybenzoic acid (IBX)-mediated oxidation in quantitative yield with no observed racemization at the stereogenic center.

**Scheme 5:**
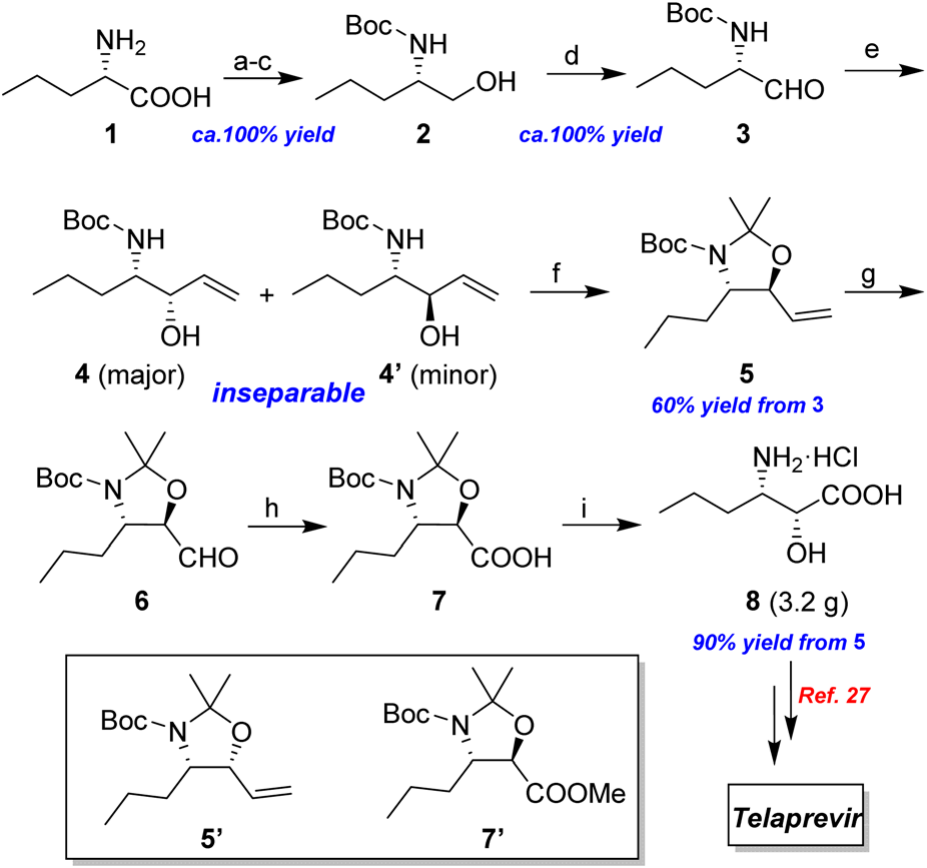
Synthesis of (2*R*,3*S*)-3-amino-2-hydroxyhexanoic acid (8). (a) SOCl_2_, MeOH, reflux, 2 h; (b) Boc_2_O, NaHCO_3_, THF/H_2_O, rt, 10 h; (c) LiAlH_4_, THF, rt, 2 h; (d) IBX, MeCN, reflux, 1 h; (e) vinylmagnesium bromide, DCM, 0 °C, 30 min; (f) DMP, *p*-TsOH, DCM, 0 °C, 30 min; (g) K_2_OsO_4_·2H_2_O, NMO, Me_2_CO/H_2_O, rt, 15 h, then NaIO_4_, rt, 1.5 h; (h) 1 M KMnO_4_, *t*-BuOH, aq. NaH_2_PO_4_, rt, 0.5 h; (i) 6 N HCl, reflux, 2 h.

Following a simple workup, vinylmagnesium bromide (2.5 equiv.) was added to a DCM solution of the aldehyde at 0 °C, resulting in an inseparable mixture of the target *syn*-amino alcohol 4 as the major product and *anti*-alcohol 4′. The *syn* diastereomer 4 can be fully converted into the corresponding *trans*-oxazolidine 5 by treatment with 2,2-dimethoxypropane and a catalytic amount of *p*-toluenesulfonic acid in DCM at 0 °C for 30 min. Under these conditions, the *cis*-oxazolidine 5′ was not formed due to torsional strain. After quenching with excess Et_3_N, the mixture was purified by flash chromatography to afford the *trans*-oxazolidine 5 as a mixture of rotamers in 60% yield over two steps.

Following the synthesis of the oxazolidine 5, the complete and efficient conversion of the allyl moiety into the carboxylic acid 7 was investigated. The use of a one-pot oxidative cleavage procedure, such as the Sharpless NaIO_4_/RuCl_3_ method^20,21^ or Lemieux-von Rudloff oxidation^22^ did not result in the clean production of acid 7 (Table 1). An increase in reaction scale resulted in complex product formation.

**Table 1:** Oxidation conditions for the *trans*-oxazolidine 5

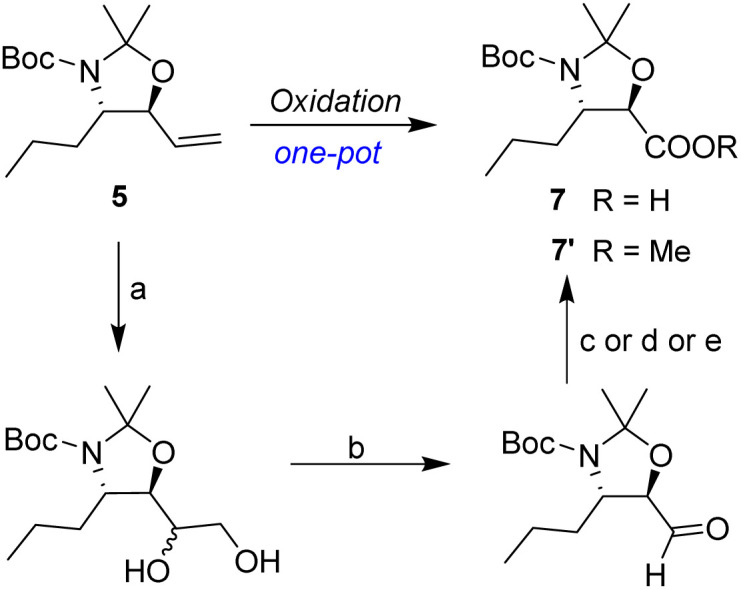		
Entry	Oxidation conditions	Result
1	NaIO_4_/RuCl_3,_ CCl_4_/MeCN/H_2_O (one-pot)	(7) Mess
2	NaIO_4_/RuCl_3,_ EA/MeCN/H_2_O (one-pot)	(7) Mess
3	NaIO_4_/KMnO_4_/NaHCO_3,_*t*-BuOH/H_2_O (one-pot)	(7) Mess
4	NaIO_4_/KMnO_4_/NaHCO_3,_ Me_2_CO/H_2_O (one-pot)	(7) Mess
5	a, b, c (stepwise)	(7) Clean *ca.* 85% yield
6	a, b, d (stepwise)	(7′) Clean *ca.* 80% yield
7	a, b, e (stepwise)	(7) Clean *ca.* 100% yield

A stepwise synthesis route was considered where the terminal alkene in 5 underwent dihydroxylation with NMO/K_2_OsO_4_·2H_2_O (as catalyst) under standard Upjohn conditions.^23^ The required diol was cleaved directly with NaIO_4_ to give the corresponding aldehyde 6 in quantitative yield without the need for work-up. The transformation of aldehyde 6 into carboxylic acid 7 was evaluated. Treatment of 6 with NaClO_2_ in the presence of NaH_2_PO_4_ and 2-methyl-2-butene as a scavenger^24^ gave the required acid 7 in 85% overall yield. Oxidation of aldehyde 6 with KOH/I_2_/MeOH system^25^ generated the corresponding methyl ester 7′ with 80% overall yield under mild conditions. Moreover, aldehyde 6 underwent effective oxidation with KMnO_4_ to the corresponding carboxylic acid 7 using a mixture of *t*-BuOH and aqueous NaH_2_PO_4_ (ref. 26) in essentially quantitative yield (Table 1).

The subsequent removal of N,O-acetonide under standard conditions, including the use of TsOH, aqueous HCl, aqueous H_2_SO_4_, and aqueous TFA is challenging. The products formed are complex due to a partial deprotection of 7. Following extensive experiments, all the protecting groups associated with 7 were fully removed by refluxing with 6 N HCl for 2 h. After a simple workup, the target enantiopure (2*R*,3*S*)-3-amino-2-hydroxyhexanoic acid product 8 was obtained as a hydrochloride salt in 90% overall yield over 3 steps from the starting compound 5, which has been used as a key intermediate for the synthesis of Telaprevir as reported by Porala.^27^ As determined by NMR spectra, the reactions take place with a very high stereoselectivity, giving only *syn*-β-amino-α-hydroxy acid 8 with complete retention of the starting configuration at C-2.

*Anti*-β-amino-α-hydroxy acids have also received considerable attention as crucial components in natural products such as perthamide C^18^ and largamide H.^28^ In order to secure ready access to the desired *anti*-β-amino-α-hydroxy acid, an inversion of the alcohol configuration in the *syn*-product 8 was studied.

Following esterification and Boc-protection, inversion of the α-hydroxy stereocenter in the corresponding compound 9 was achieved using a standard Mitsunobu procedure^29^ with *p*-nitrobenzoic acid, diisopropyl azodicarboxylate (DIAD) and PPh_3_, affording 10 in 85% yield. A subsequent mild saponification was conducted using K_2_CO_3_ in MeOH to produce *anti-N*-Boc-β-amino-α-hydroxy acid methyl ester 11 in 80% yield with complete inversion of the stereochemical configuration of the alcohol starting material determined by NMR spectra (Scheme 6).

**Scheme 6:**
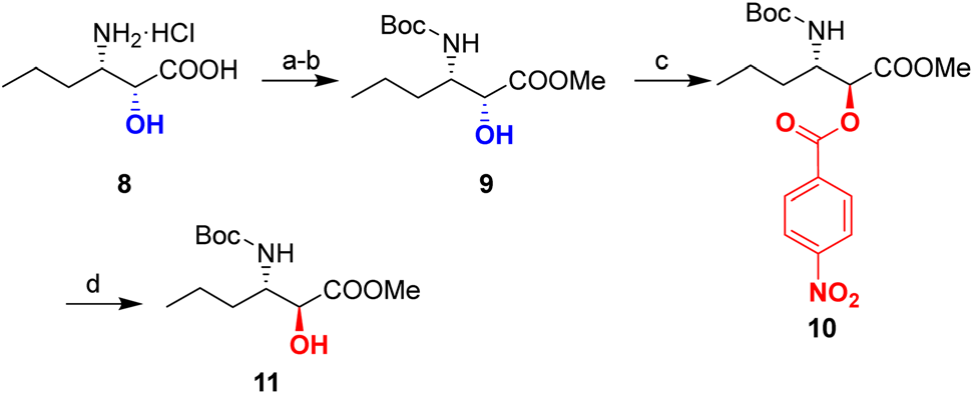
Synthesis of *anti-N*-Boc-β-amino-α-hydroxy acid methyl ester (11). (a) SOCl_2_, MeOH, reflux, 2 h; (b) Boc_2_O, NaHCO_3_, THF/H_2_O, rt, 10 h; (c) *p*-nitrobenzoic acid, DIAD, PPh_3_, THF, rt, 2 h; (d) K_2_CO_3_, MeOH, 0 °C, 20 min.

The general applicability of these optimal conditions was examined in the preparation of other *syn*-β-amino-α-hydroxy acids from α-amino acids: the results are given in Tables 2 and 3. All the reactions delivered the target products in high overall yield. Details regarding experimental procedures are provided in the SI (SI).

**Table 2:** Synthesis of *syn*-β-amino-α-hydroxy acids (8a–8k) from α-amino acids (1a–1k)

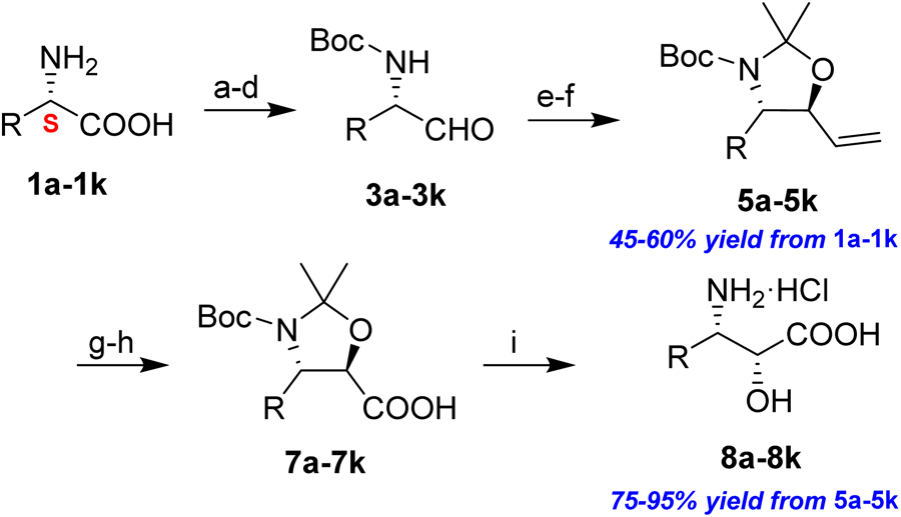			
Entry	Starting material (S)-α-amino acid (1a–1k)	*trans*-Oxazolidine (5a–5k)	*syn*-β-amino-α-hydroxy acid hydrochloride salt (8a–8k)
1	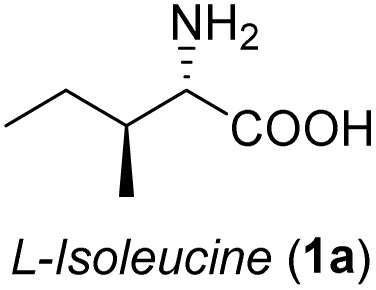	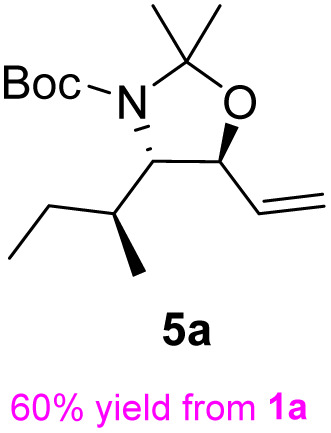	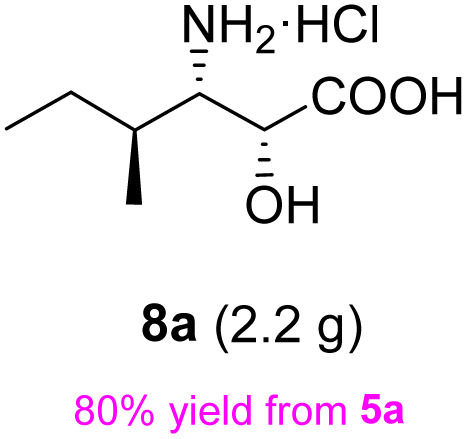
2	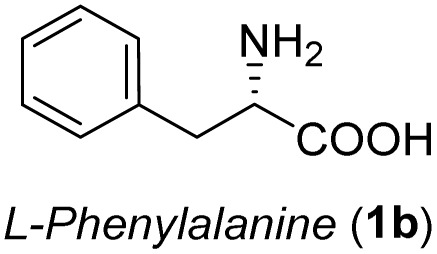	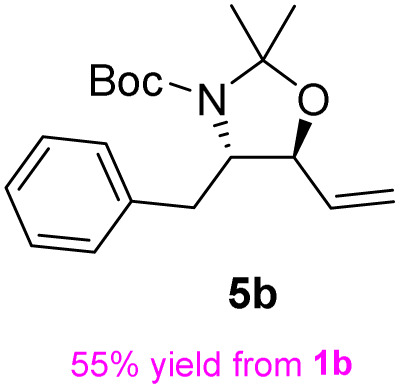	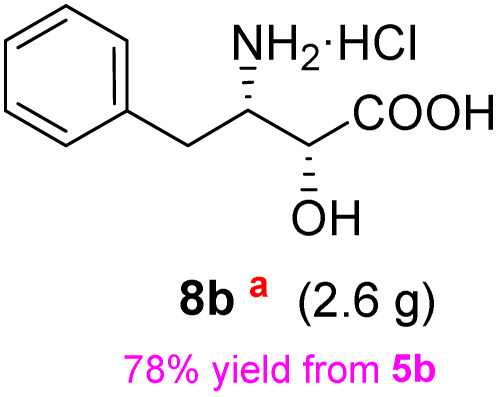
3	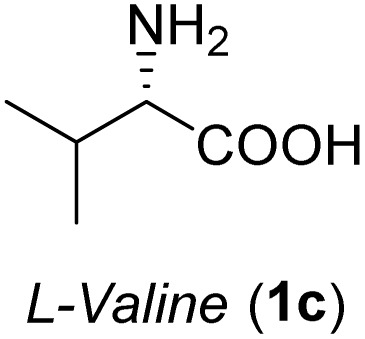	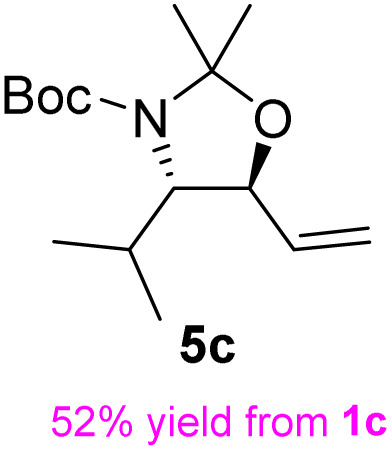	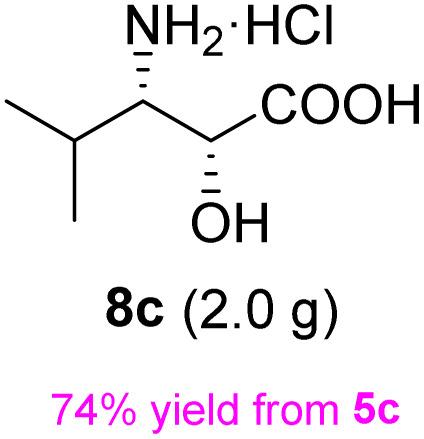
4	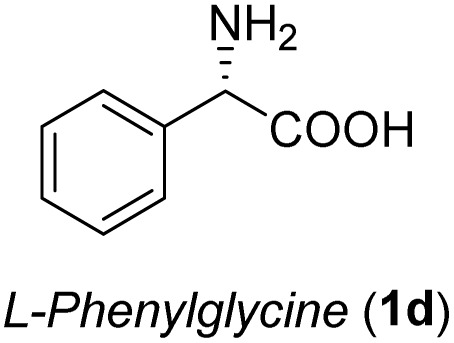	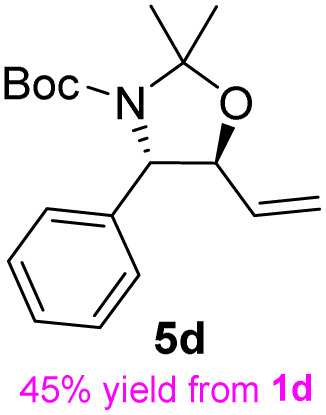	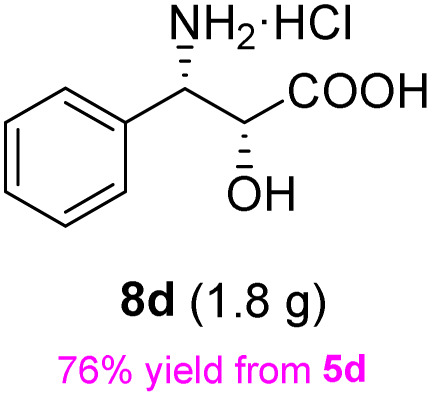
5	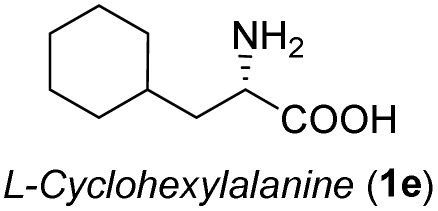	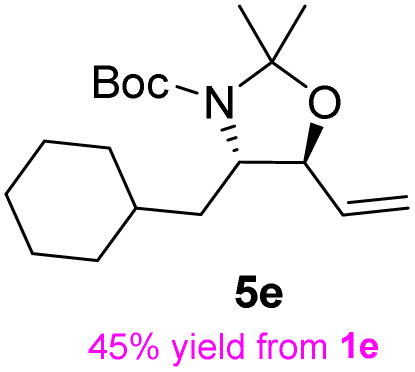	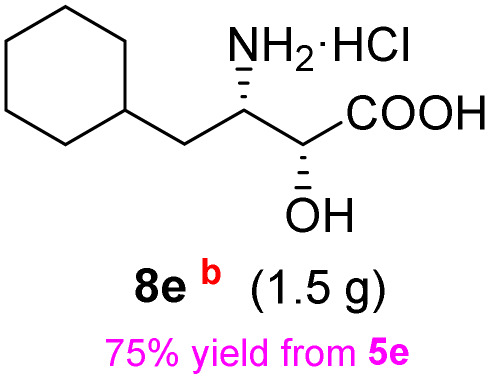
6	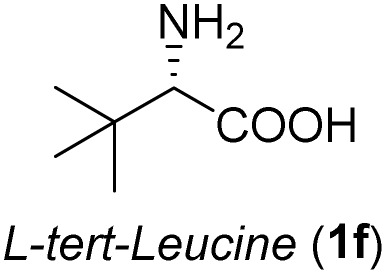	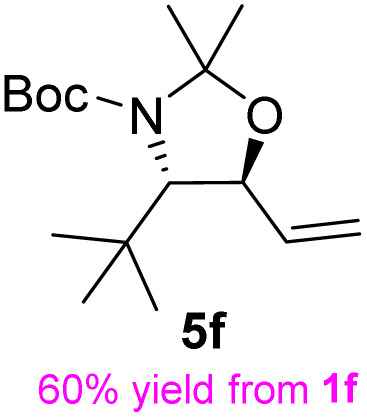	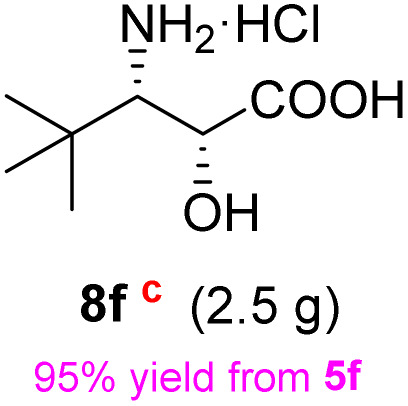
7	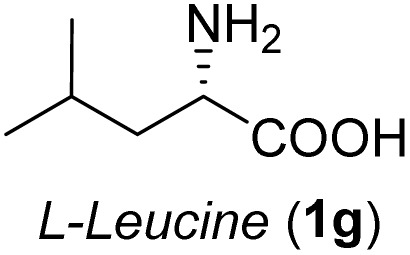	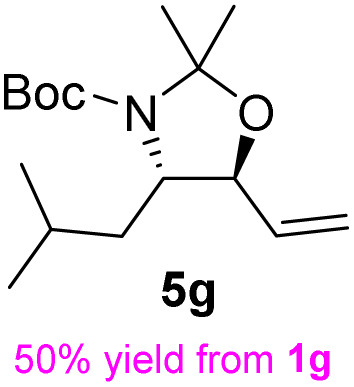	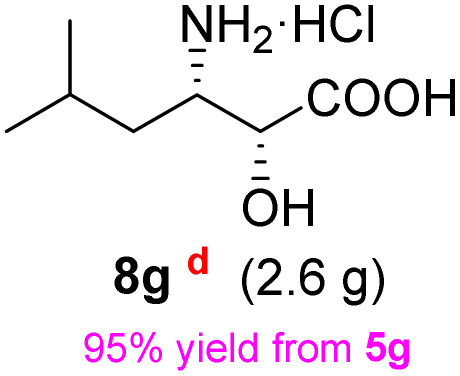
8	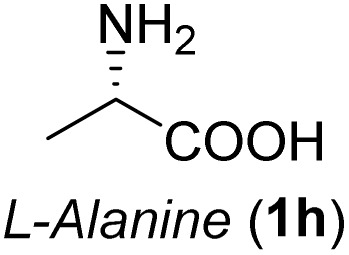	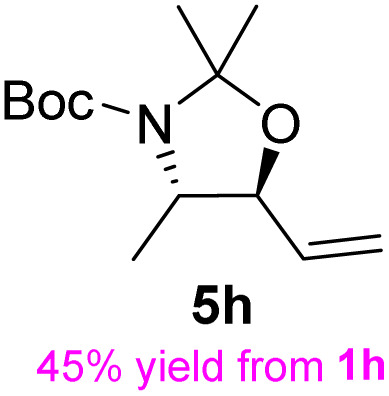	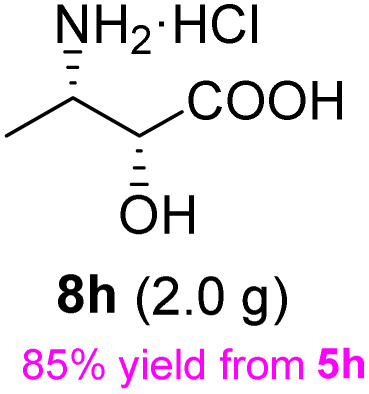
9	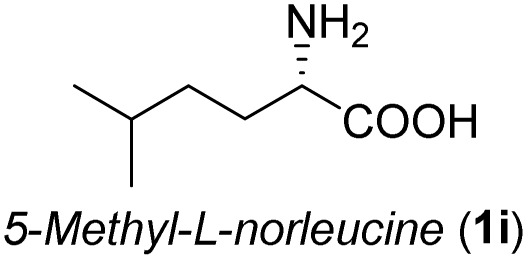	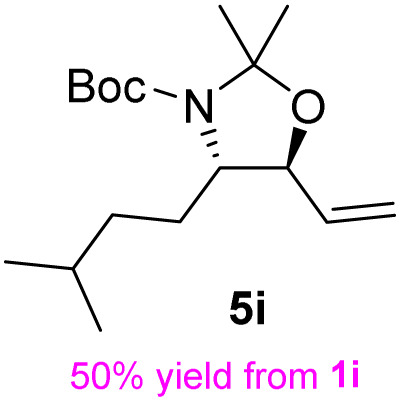	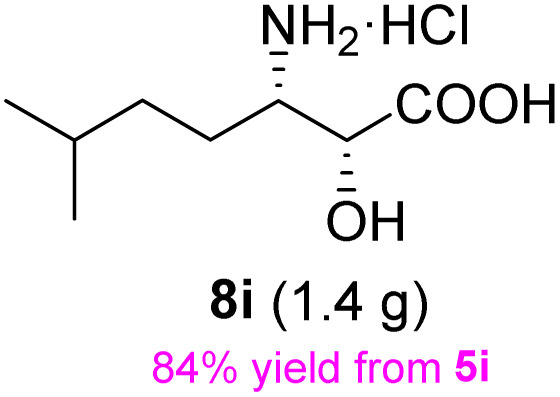
10	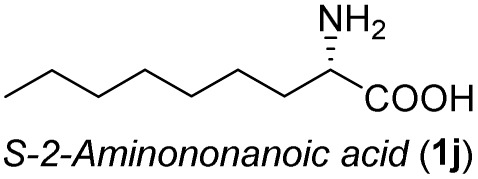	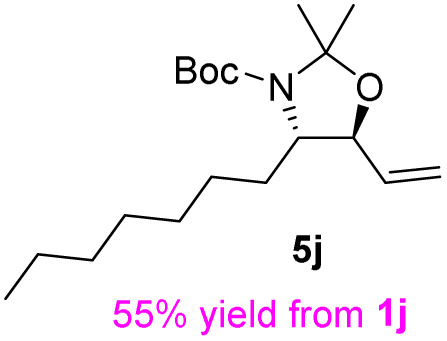	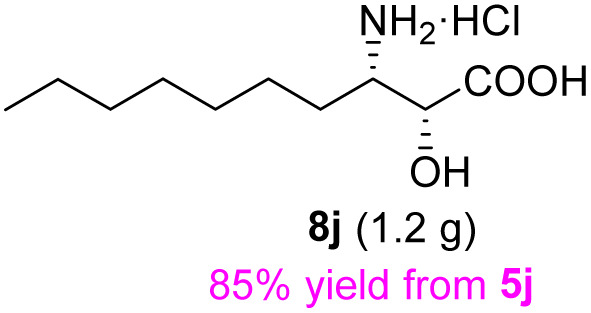
11	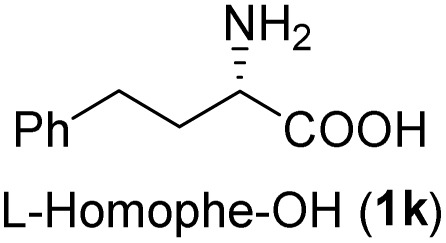	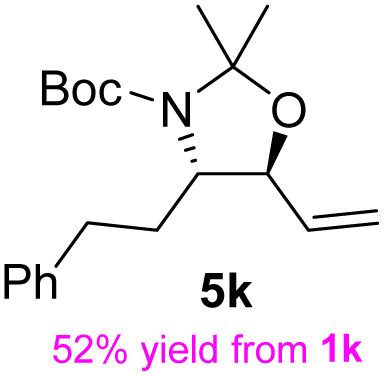	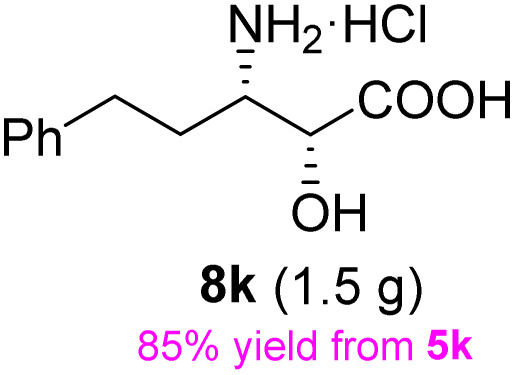

a Key component of Taxol.

b Key component of KRI-1314.

c Key component of BMS-275183.

d Key component of ABT-271and KRI-1230.

**Table 3:** Synthesis of *syn*-β-amino-α-hydroxy acids (8l–8p) from α-amino acids (1l–1p)

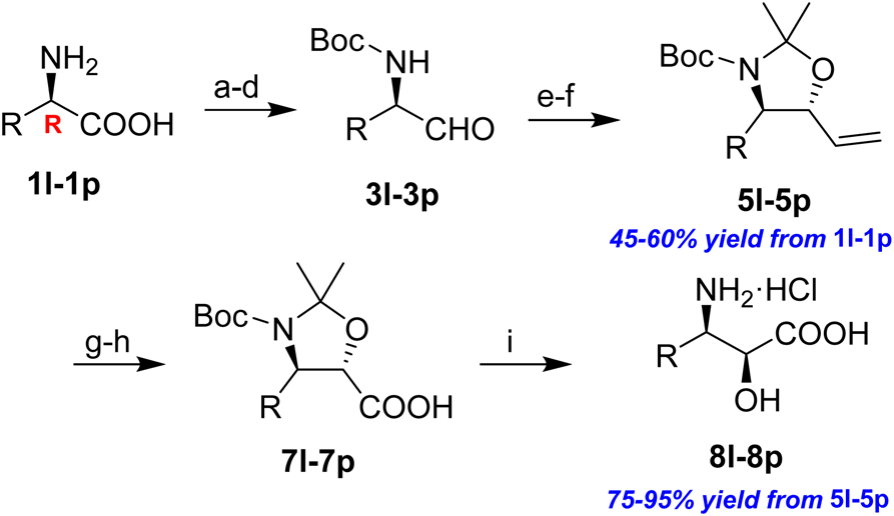			
Entry	Starting material (R)-α-amino acid (1l–1p)	*trans*-Oxazolidine (5l–5p)	*syn*-β-amino-α-hydroxy acid hydrochloride salt (8l–8p)
1	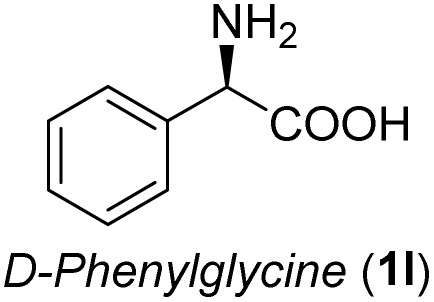	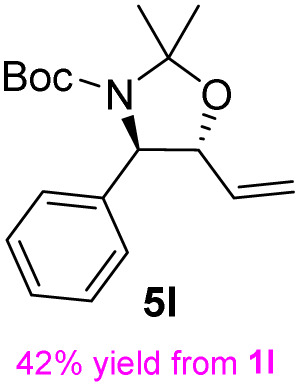	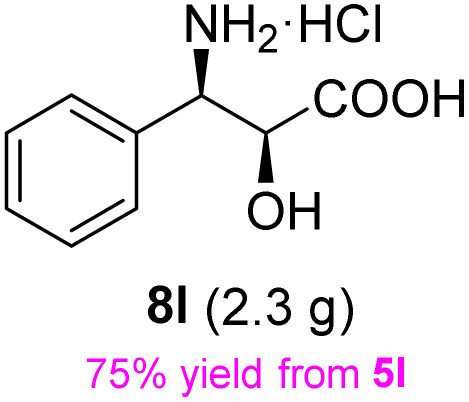
2	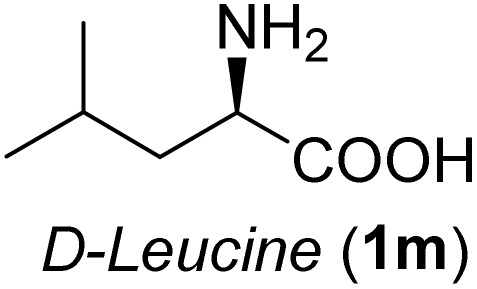	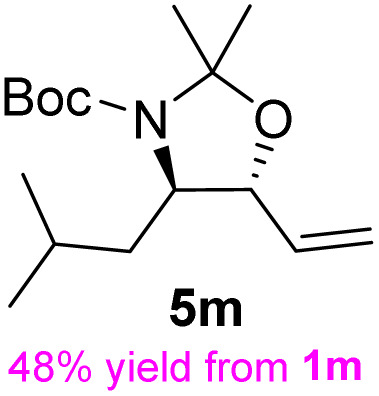	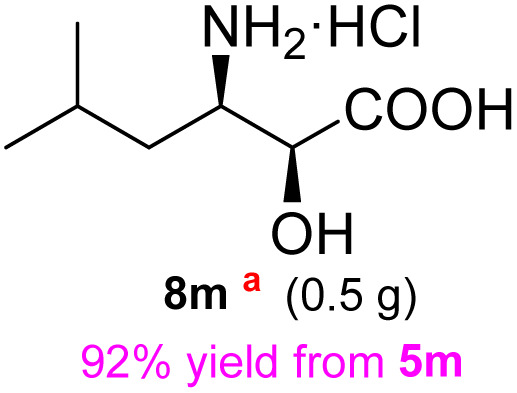
3	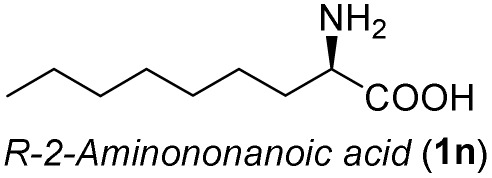	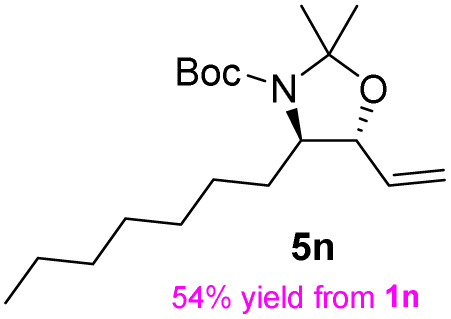	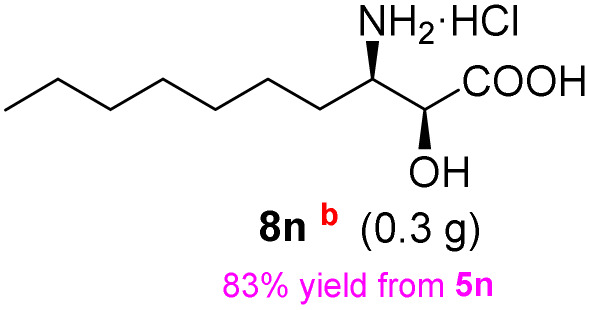
4	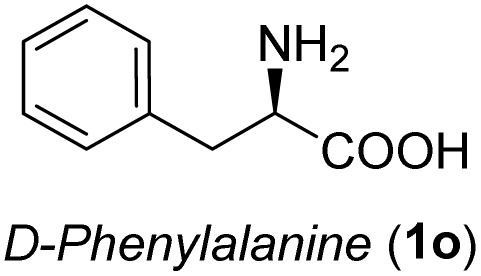	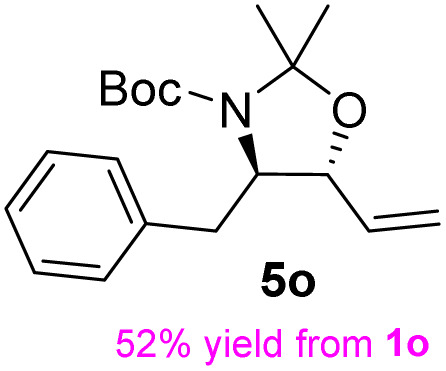	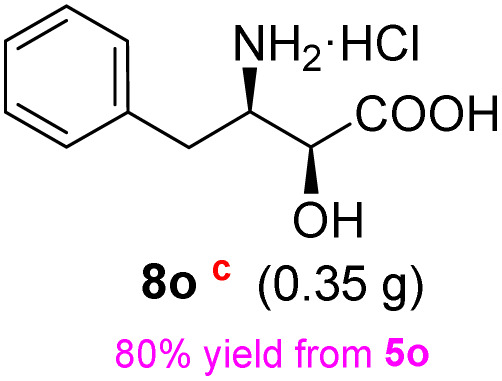
5	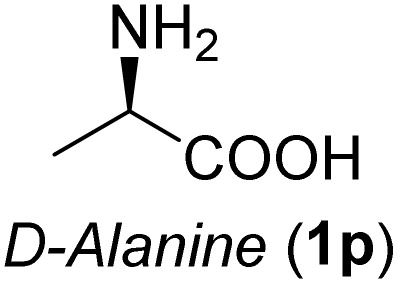	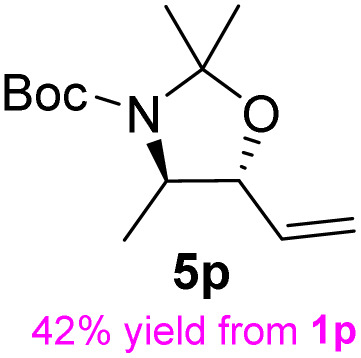	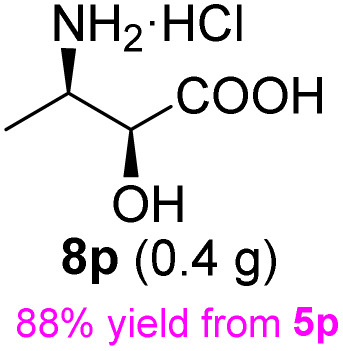

a Key component of Amastatin.

b Key component of Microginin.

c Key component of Bestatin, Phebestin and Probestin.

In order to demonstrate the application of the proposed method in terms of “total” synthesis, we have conducted the formal syntheses of the corresponding biologically active molecules. In addition, we derivatized compound 7l by methylation^30^ and converting the phenyl moiety *via* oxidative cleavage with NaIO_4_/RuCl_3_ to the carboxylic acid,^31^ producing the acid 12 in good yield. Following global deprotection with 6 N HCl, l-*threo*-3-hydroxyaspartic acid 13, which is a potent excitatory amino acid transporter (EAAT) inhibitor and a crucial component of Rakicidin A, was synthesized as the hydrochloride salt in 70% yield over five steps from 5l (Scheme 7). The production of 13 represents the formal syntheses of l-TFB-TBOA as 13 had been used to generate the complex amino acid by Poelarends.^32^ It should be noted that l-TFB-TBOA exhibits nanomolar affinity for EAAT1 and EAAT2 and lacks affinity with respect to glutamate-gated ion channels.

**Scheme 7:**
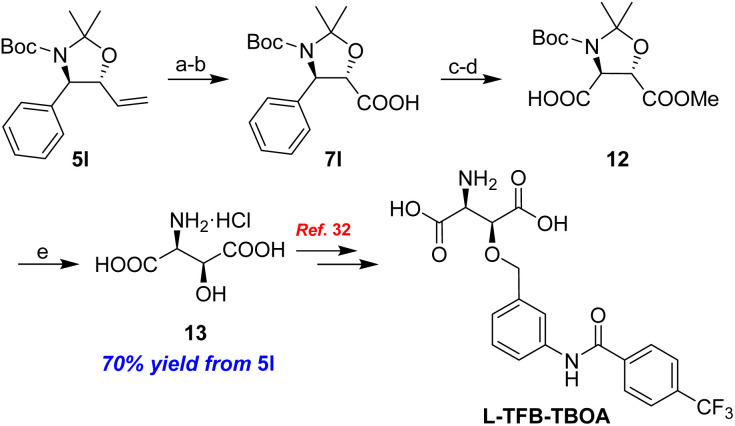
Formal synthesis of l-TFB-TBOA. (a) K_2_OsO_4_·2H_2_O, NMO, Me_2_CO/H_2_O, rt, 16 h, then NaIO_4_, rt, 1.5 h; (b) 1M KMnO_4_, *t*-BuOH, aq. NaH_2_PO_4_, rt, 0.5 h; (c) MeI, K_2_CO_3_, Me_2_CO, reflux, 3 h; (d) NaIO_4_, RuCl_3_, CCl_4_/MeCN/H_2_O, reflux, 3 h; (e) 6 N HCl, reflux, 2 h.

As shown in Scheme 8, dihydroxylation of the olefin in 5k followed by glycol cleavage with sodium periodate produced the corresponding aldehyde, which was reduced with NaBH_4_ to afford the alcohol 14 in 95% yield over three steps. The acetonide group of 14 can be readily deprotected to generate 15 in quantitative yield by treatment with a methanolic solution of *p*-TsOH. The conversion of the 15 diastereomer to (S)-vigabatrin has been reported.^33,34^ Therefore, the sequence presented in this study constitutes a formal synthesis of (S)-vigabatrin, which serves as an irreversible gamma-aminobutyric acid (GABA)-transaminase inhibitor. The S-isomer is pharmacologically active, whereas the R-isomer is inactive.

**Scheme 8:**
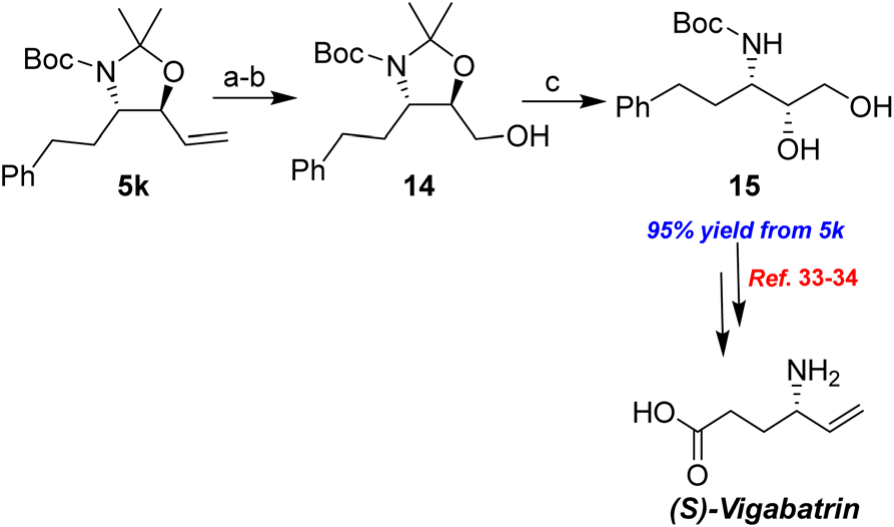
Formal synthesis of (S)-vigabatrin. (a) K_2_OsO_4_·2H_2_O, NMO, Me_2_CO/H_2_O, rt, 14 h, then NaIO_4_, rt, 1.5 h; (b) NaBH_4_, MeOH, rt, 1 h; (c) *p*-TsOH, MeOH, rt, 12 h.

## Conclusions

In summary, we have developed a practical synthetic route to enantiopure *syn*-β-amino-α-hydroxy acid from α-amino acid that exhibits a high level of stereoselectivity. Following the addition of a vinyl Grignard reagent to a *N*-Boc-α-amino aldehyde, kinetic resolution accompanied acetonide formation at 0 °C for 30 min to give the corresponding *trans*-oxazolidine diastereo-selectively in good yield. A dihydroxylation-oxidative cleavage sequence of the terminal olefin in *trans*-oxazolidine afforded the aldehyde, which underwent side-chain oxidation to deliver the carboxylic acid in very high yield. A final global deprotection in refluxing 6 N hydrochloric acid generated *syn*-β-amino-α-hydroxy acid as the hydrochloride salt. Inversion of the α-hydroxy stereocenter of the *syn-N*-Boc-β-amino-α-hydroxy acid methyl ester was completed in a Mitsunobu reaction with subsequent saponification to give the *anti-N*-Boc-β-amino-α-hydroxy acid methyl ester in good yield.

The synthesis of l-*threo*-3-hydroxyaspartic acid 13 and *N*-Boc-aminodiol 15 represents a formal approach to the total synthesis of l-TFB-TBOA and (S)-vigabatrin. The proposed procedure offers a viable alternative to current methods for preparing enantiopure *syn*-β-amino-α-hydroxy acids and represents a viable route in the preparation of a variety of biologically important compounds containing this crucial amino acid moiety. Our strategy represents a simple and scalable process with a low environmental impact. Further work is now in progress.

## Conflicts of interest

There are no conflicts to declare.

## Supplementary Material

RA-015-D5RA05586E-s001

## Data Availability

The authors declare that the data supporting the findings of this study are available within the paper and its Supplementary Information files (SI). Should any raw data files be needed in another format they are available from the corresponding author upon reasonable request. Source data are provided with this paper. Supplementary information is available. See DOI: https://doi.org/10.1039/d5ra05586e.

## References

[cit1] Gallego-Jara J., ozano-Terol G. L., Sola-Martinez R. A., Canovas-Diaz M., Diego Puente T., de Diego Puente T. (2020). Molecules.

[cit2] Li L., Thomas S. A., Klein L. L., Yeung C. M., Maring C. J., Grampovnik D. J., Lartey P. A., Plattner J. J. (1994). J. Med. Chem..

[cit3] Pathuri G., Thorpe J. E., Disch B. C., Bailey-Downs L. C., Ihnat M. A., Gali H. (2013). Bioorg. Med. Chem. Lett..

[cit4] Okino T., Matsuda H., Murakami M., Yamaguchi K. (1993). Tetrahedron Lett..

[cit5] Rich D. H., Moon B. J., Harbeson S. (1984). J. Med. Chem..

[cit6] Yamazaki Y., Kunimoto S., Ikeda D. (2007). Biol. Pharm. Bull..

[cit7] Etoh Y., Miyazaki M., Saitoh H., Toda N. (1993). Jpn. J. Pharmacol..

[cit8] Cui Y., Zhang M., Xu H., Zhang T., Zhang S., Zhao X., Jiang P., Li J., Ye B., Sun Y., Wang M., Deng Y., Meng Q., Liu Y., Fu Q., Lin J., Wang L., Chen Y. (2022). J. Med. Chem..

[cit9] Long B., Pu L., Liu Z., Zeng X., Wu Z. (2023). Org. Biomol. Chem..

[cit10] Klibanov O. M., Williams S. H., Smith L. S., Olin J. L., Vickery S. B. (2011). Pharmacotherapy.

[cit11] Fusetani N., Matsunaga S. (1990). J. Am. Chem. Soc..

[cit12] Murakami Y., Takei M., Shindo K., Kitazume C., Tanaka J., Higa T., Fukamachi H. (1998). J. Nat. Prod..

[cit13] Issac M., Aknin M., Gauvin-Bialecki A., Voogd N. D., Ledoux A., Frederich M., Kashman Y., Carmeli S. (2017). J. Nat. Prod..

[cit14] Robello M., Barresi E., Baglini E., Salerno S., Taliani S., Settimo F. D. (2021). J. Med. Chem..

[cit15] Kohr M., Walt C., Dastbaz J., Müller R., Kazmaier U. (2022). Org. Biomol. Chem..

[cit16] Johnson E. P., Hubieki M. P., Combs A. P., Teleha C. A. (2011). Synthesis.

[cit17] Göhl M., Zhang L., El Kilani H., Sun X., Zhang K., Brönstrup M., Hilgenfeld R. (2022). Molecules.

[cit18] Sepe V., D'Auria M. V., Bifulco G., Ummarino R., Zampella A. (2010). Tetrahedron.

[cit19] Li G., Chang H. T., Sharpless K. B. (1996). Angew. Chem., Int. Ed. Engl..

[cit20] Commandeur M., Commandeur C., Cossy J. (2011). Org. Lett..

[cit21] Zimmermann F., Meux E., Mieloszynski J. L., Lecuire J. M., Oget N. (2005). Tetrahedron Lett..

[cit22] Expósito A., Fernández-Suárez M., Iglesias T., Munoz L., Riguera R. (2001). J. Org. Chem..

[cit23] VanRheenen V., Kelly R. C., Cha D. Y. (1976). Tetrahedron Lett..

[cit24] Bal B. S., Childers Jr W. E., Pinnick H. W. (1981). Tetrahedron.

[cit25] Yamada S., Morizono D., Yamamoto K. (1992). Tetrahedron Lett..

[cit26] Abiko A., Roberts J. C., Takemasa T., Masamune S. (1986). Tetrahedron Lett..

[cit27] Porala S., Yerrabelly J. R., Kasireddy V. R., Yerrabelly H., Ghojala V. R., Rebelli P. (2019). ChemistrySelect.

[cit28] Plaza A., Bewley C. A. (2006). J. Org. Chem..

[cit29] Swamy K. C. K., Kumar N. N. B., Balaraman E., Kumar K. V. P. P. (2009). Chem. Rev..

[cit30] Seo Y., Kim H., Chae D. W., Kim Y. G. (2014). Tetrahedron: Asymmetry.

[cit31] Novak T., Tan Z., Liang B., Negishi E. (2005). J. Am. Chem. Soc..

[cit32] Fu H., Younes S. H. H., Saifuddin M., Tepper P. G., Zhang J., Keller E., Heeres A., Szymanski W., Poelarends G. J. (2017). Org. Biomol. Chem..

[cit33] Alcón M., Poch M., Moyano A., Pericàs M. A., Riera A. (1997). Tetrahedron: Asymmetry.

[cit34] Dagoneau C., Tomassini A., Denis J. N., Vallée Y. (2001). Synthesis.

