# Correction: A practical synthesis of enantiopure *syn*-β-amino-α-hydroxy acids from α-amino acids with application in the formal syntheses of l-TFB-TBOA and (*S*)-vigabatrin

**DOI:** 10.1039/d5ra90118a

**Published:** 2025-10-20

**Authors:** Bohua Long, Peng Zhang, Mengmeng Jiang, Pengfei Guo, Xuanluan Chen, Shunlai Shang, Zhengzhi Wu

**Affiliations:** a The First Affiliated Hospital of Shenzhen University, Shenzhen Second People's Hospital Shenzhen 518035 China bhlong121@163.com szwzz001@163.com; b Shenzhen Institute of Geriatric Medicine Shenzhen China; c Department of Nephrology, China-Japan Friendship Hospital Beijing China 18810568600@163.com

## Abstract

Correction for ‘A practical synthesis of enantiopure *syn*-β-amino-α-hydroxy acids from α-amino acids with application in the formal syntheses of l-TFB-TBOA and (*S*)-vigabatrin’ by Bohua Long *et al.*, *RSC Adv.*, 2025, **15**, 35635–35641, https://doi.org/10.1039/D5RA05586E.

The authors regret an error in the spelling of Shunlai Shang's name in the original article. The correct spelling is as shown in this Correction article.

The Royal Society of Chemistry apologises for these errors and any consequent inconvenience to authors and readers.

