# Supplementary material for: A practical synthesis of enantiopure syn-β-amino-α-hydroxy acids from α-amino acids with application in the formal syntheses of l-TFB-TBOA and (S)-vigabatrin
Source: RSC Adv. 2025 Sep 26;15(42):35635–41. doi: 10.1039/d5ra05586e (PMC12466934; doi:10.1039/d5ra05586e)
Supplement: RA-015-D5RA05586E-s001 [file RA-015-D5RA05586E-s001.pdf]

Supplementary Information (SI) for RSC Advances.
This journal is © The Royal Society of Chemistry 2025

A practical synthesis of enantiopure *syn*-β-amino-α-hydroxy acids from α-amino acids with application in the formal syntheses of L-TFB-TBOA and (S)-vigabatrin

Content

General Experimental **page 2**

Experimental Procedures **page 3**

NMR Spectra of New Compounds and Selected Known Compounds **page 22**

General Experimental

Commercially available reagents were used without further purification unless otherwise stated. All solvents were distilled prior to use: toluene, benzene, diethyl ether and tetrahydrofuran were distilled from Na/benzophenone; while DCM, dimethylformamide, acetonitrile, triethylamine and diisopropylethylamine were distilled from CaH_2. Methanol was distilled under a N_2 atmosphere from Mg/I_2. All reactions were conducted in oven-dried (120 °C) or flame-dried glasswares under a N_2 atmosphere, and at ambient temperature (20 to 25 °C) unless otherwise stated. All non-aqueous reactions were performed by standard syringe in septa techniques. Evaporation and concentration under reduced pressure was performed at 50-500 mbar. ^{1}H NMR spectra were recorded in $CDCl_3$ (unless stated otherwise) on a Bruker Avance AV600 at 600 MHz (151 MHz). Chemical shifts are reported as δ values (ppm) referenced to either a tetramethylsilane (TMS) internal standard or the signals due to the solvent residual. Data for ^{1}H NMR are reported as follows: chemical shift (*δ* ppm), multiplicity (s = singlet, brs = broad singlet, d = doublet, t = triplet, q = quartet, m = multiplet), coupling constant (Hz), integration. Some peptide intermediates exist as rotational conformers, the chemical shift for the minor isomers were indicated using parentheses next to the peak for their major isomers. Mass spectra were measured on ABI Q-star Elite. Optical rotations were measured on a Perkin-Elmer 351 polarimeter at 589 nm with a 100 mm path length cell at 20 °C (reported as follows: concentration (*c* in g/100 mL), solvent). The reaction progresses were checked on pre-coated thin layer chromatography (TLC) plates. TLC was carried out using pre-coated sheets (Qingdao silica gel 60-F250, 0.2 mm) which, after development, were visualized under UV light at 254nm. Flash column chromatography was performed using the indicated solvents on E. Qingdao silica gel 60 (230-400 mesh ASTM). Yields refer to chromatographically purified compounds, unless otherwise stated.

Experimental procedures

General Procedure A (GPA):

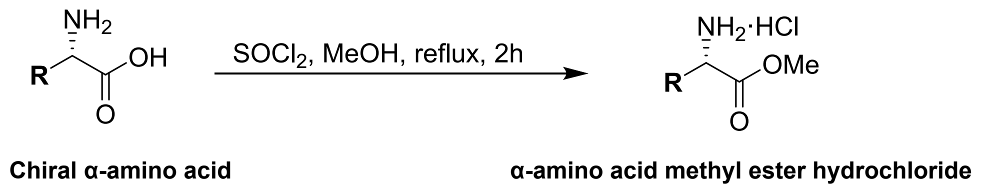

To a solution of **Chiral α-amino acid** (10 mmol, *1.0 eq.*) in MeOH (100 mL) was dropwise added $SOCl_2$ (2.2 mL, 30 mmol, *3.0 eq.*) at 0 °C. After 30 min, the resultant mixture was heated to reflux and stirred for 2h. The solution was cooled to room temperature and volatiles of the reaction mixture were removed in vacuo to obtain the **α-amino acid methyl ester hydrochloride** in essentially quantitative yield, which was used for next step directly.

(**Textbook synthetic method**)

General Procedure B (GPB):

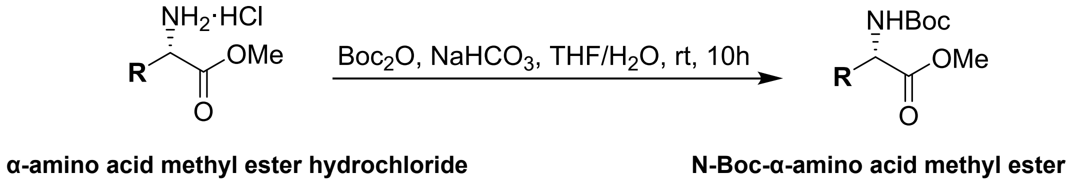

To a solution of the above-obtained **α-amino acid methyl ester hydrochloride** (10 mmol, *1.0 eq.*) in THF/H_2O (1:1) (100 mL) was added $NaHCO_3$ (2.52 g, 30 mmol, *3.0 eq.*) and Boc_2O (2.3 mL, 10 mmol, *1.0 eq.*) at 0 °C. After being stirred at room temperature for 10 h, volatiles of the reaction mixture were removed in vacuo. The solution was then diluted with water (200 mL). The aqueous phase was extracted with ethyl acetate for 3 times (3×300 mL). The combined organic phase was washed by brine (200 mL), dried over sodium sulfate (anhydrous) and concentrated in *vacuo* to give the **N-Boc-α-amino acid methyl ester** in essentially quantitative yield, which was used for next step directly.

(**Textbook synthetic method**)

General Procedure C (GPC):

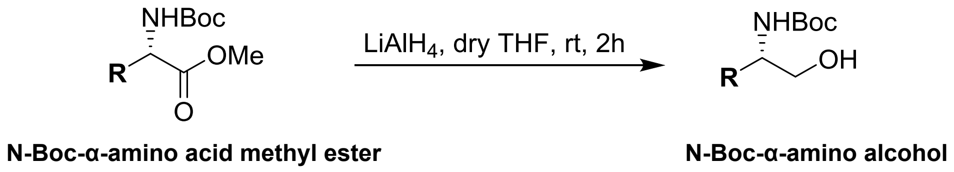

To a solution of the above-obtained **N-Boc-α-amino acid methyl ester** (10 mmol, *1.0 eq.*) in dry THF (100 mL) was added $LiAlH_4$ (0.57 g, 15 mmol, *1.5 eq.*) at temperatures below -50 °C. After 30 min, the resultant mixture was allowed to warm to room temperature slowly and stirred for 2h. The reaction was then quenched with saturated aqueous Na_2SO_4 solution (100 mL) at temperatures below -50 °C. Volatiles of the reaction mixture were removed in vacuo. The solution was then diluted with water (200 mL). The aqueous phase was extracted with ethyl acetate for 3 times (3×300 mL). The combined organic phase was washed by brine (200 mL), dried over sodium sulfate (anhydrous) and concentrated in *vacuo* to give the **N-Boc-α-amino alcohol** in essentially quantitative yield, which was used for next step directly.

(**Textbook synthetic method**)

General Procedure D (GPD):

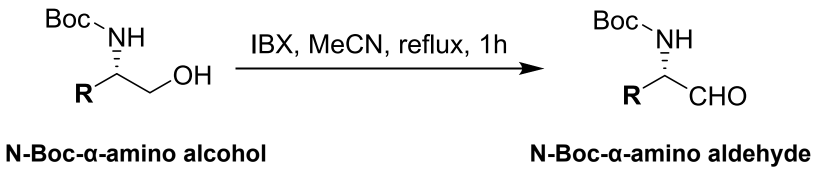

To a solution of the above-obtained **N-Boc-α-amino alcohol** (10 mmol, *1.0 eq.*) in dry MeCN (100 mL) was added IBX (3.36 g, 12 mmol, *1.2 eq.*) at room temperature. After 15 min, the resultant mixture was heated to reflux and stirred for 1h. The solution was cooled to room temperature and the solid was removed by filtration through a pad of celite and washed with MeCN (100 mL). The total filtrate was concentrated in vacuo to afford the **N-Boc-α-amino aldehyde** in essentially quantitative yield, which was used immediately for next step directly. (**Textbook synthetic method**)

General Procedure E (GPE):

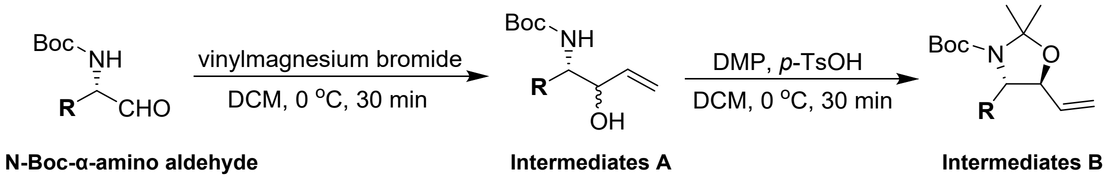

To a solution of the above-obtained **N-Boc-α-amino aldehyde** (10 mmol, *1.0 eq.*) in dry DCM (100 mL) was added vinylmagnesium bromide (1.0 M solution in THF) (25 mL, *2.5 eq.*) at 0 °C. After being stirred at 0 °C for 30 min, the reaction mixture was quenched with saturated aqueous solution of NH_4Cl (100 mL). Volatiles of the mixture were removed in vacuo. The solution was then diluted with water (200 mL). The aqueous phase was extracted with ethyl acetate for 3 times (3×300 mL). The combined organic phase was washed by brine (200 mL), dried over sodium sulfate (anhydrous) and concentrated in *vacuo* to give the **Intermediates A**, which was used for next step immediately and directly.

To a solution of the above-obtained **Intermediates A** in dry DCM (100 mL) was added *p*-TsOH (86 mg, 0.5 mmol, *0.05 eq.*) and 2,2-dimethoxypropane (DMP) (3.7 mL, 30 mmol, *3.0 eq.*) at 0 °C. After being stirred at 0 °C for 30 min, the reaction mixture was immediately quenched with excess Et_3N (0.28 mL, 2 mmol, *0.2 eq.*), then concentrated in *vacuo,* then purified by flash silica gel column chromatography (EA/PE=1/20) to afford the **Intermediates B**.

(**Structural Characterization by ^{1}HNMR, ^{13}CNMR and HRMS**)

General Procedure F (GPF):

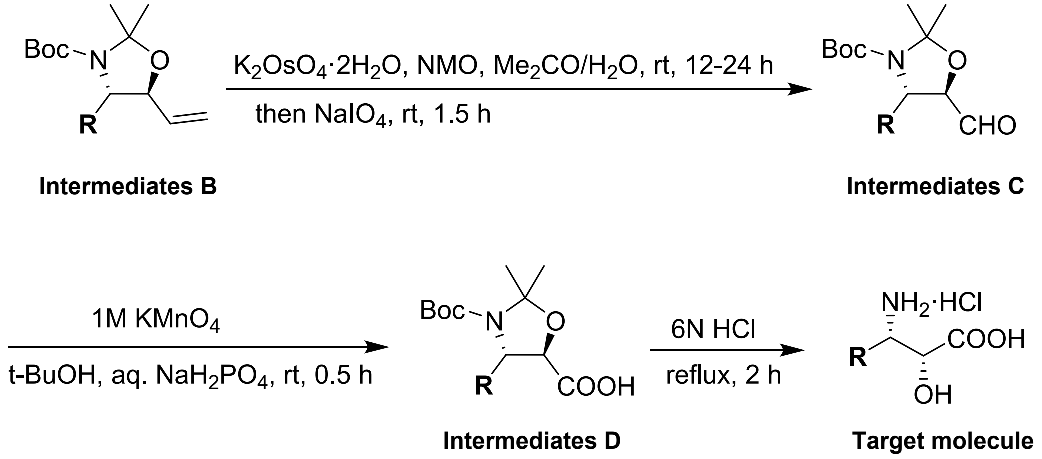

To a solution of the above-obtained **Intermediates B** (10 mmol, *1.0 eq.*) in Me_2CO/H_2O (5:1) (120 mL) was added $K_2OsO_4 \cdot 2H_2O$ (37 mg, 0.1 mmol, *0.01 eq.*) and NMO (5.9 g, 50 mmol, *5.0 eq.*). The mixture was stirred at room temperature for 12 - 24 h until all the starting material had been consumed (monitored by TLC).

Then $NaIO_4$ (4.3 g, 20 mmol, *2.0 eq.*) and H_2O (50 mL) were added. The reaction mixture was stirred vigorously for 1.5 h, then quenched with saturated aqueous solution of Na_2SO_3 (200 mL). Volatiles of the mixture were removed in vacuo. The solution was then diluted with water (200 mL) and adjusted to pH 2 by slow addition of solid $KHSO_4$. The aqueous phase was extracted with ethyl acetate for 3 times (3×400 mL). The combined organic phase was washed by brine (300 mL), dried over sodium sulfate (anhydrous) and concentrated in *vacuo* to give the **Intermediates C**, which was used for next step immediately and directly.

To a solution of the above-obtained **Intermediates C** in *t*-BuOH/NaH_2PO_4 (5% aqueous solution) (5:3) (160 mL) was added $KMnO_4$ (1M aqueous solution) (60 mL, 60 mmol, *6.0 eq.*). After being stirred at room temperature for 0.5 h, the reaction mixture was quenched with saturated aqueous solution of Na_2SO_3 (200 mL). The solution was then diluted with water (200 mL) and adjusted to pH 2 by slow addition of solid $KHSO_4$. The aqueous phase was extracted with ethyl acetate for 3 times (3×400 mL). The combined organic phase was washed by brine (300 mL), dried over sodium sulfate (anhydrous) and concentrated in *vacuo* to give the **Intermediates D**, which was used for next step immediately and directly.

The above-obtained **Intermediates D** was suspended in 6N HCl (150 mL) and refluxed for 2 h. The reaction mixture was cooled to room temperature, then diluted with water (200 mL). The aqueous phase was extracted with ethyl acetate for 2 times (2×400 mL) to remove any organic soluble impurities. The aqueous phase was then evaporated under reduced pressure. The residue was then co-evaporated with MeCN for 2 times (2×100 mL) to provide the desired **Target molecule** as a light yellow solid.

(**Structural Characterization by ^{1}HNMR, ^{13}CNMR and HRMS**)

1) Preparation of (2R,3S)-3-amino-2-hydroxyhexanoic acid hydrochloride (8)

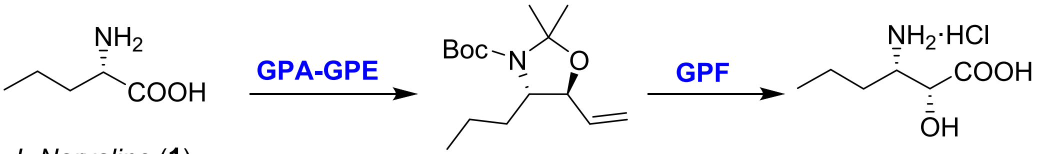

L-Norvaline (**1**) **5** **8**

60% yield from **1** 90% yield from **5**

Compound **5** :

[α]$^{25}_{D}$ +22.4 (c 0.54, EtOAc); ^{1}H NMR (600 MHz, $CDCl_3$) (*exists as rotamers*) δ 5.91 (ddd, *J* = 17.3, 10.3, 7.2 Hz, 1H), 5.33 (d, *J* = 17.1 Hz, 1H), 5.22 (d, *J* = 10.3 Hz, 1H), 4.31 – 4.16 (m, 1H), 3.73 – 3.52 (m, 1H), 1.68 – 1.56 (m, 5H), 1.50 (s, 3H), 1.47 (s, 9H), 1.33 – 1.25 (m, 2H), 0.93 (t, *J* = 7.3 Hz, 3H); ^{13}C NMR (151 MHz, $CDCl_3$) (*exists as rotamers*) δ 151.92, 137.52, 117.84, 94.30, 93.77, 80.80, 79.63, 61.60, 36.61, 28.41, 27.12, 18.49, 14.03; HR-ESIMS m/z: calculated for $C_{15}H_{27}NO_3Na^+$ $[M+Na]^+$: 292.1883, found 292.1885.

Compound **8** :

[α]$^{25}_{D}$ +1.6 (c 0.49, H_2O); ^{1}H NMR (600 MHz, D_2O) δ 4.41 (d, *J* = 3.6 Hz, 1H), 3.61 (td, *J* = 7.1, 3.6 Hz, 1H), 1.78 – 1.71 (m, 1H), 1.66 – 1.60 (m, 1H), 1.45 – 1.38 (m, 2H), 0.92 (t, *J* = 7.3 Hz, 3H).; ^{13}C NMR (151 MHz, D_2O) δ 174.55, 69.11, 52.99, 30.95, 17.98, 12.78; HR-ESIMS m/z: calculated for $C_6H_{14}NO_3^+$ $[M+H]^+$ (free amine): 148.0974, found 148.0975.

2) Preparation of (2R,3S,4S)-3-amino-2-hydroxy-4-methylhexanoic acid hydrochloride (8a)

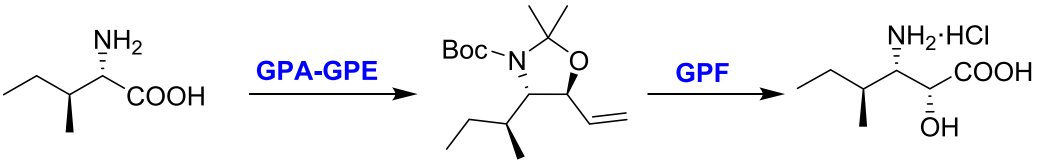

L-Isoleucine (**1a**) **5a** **8a**

60% yield from **1a** 80% yield from **5a**

Compound **5a** :

[α]$^{25}_{D}$ -2.0 (c 0.44, EtOAc); ^{1}H NMR (600 MHz, $CDCl_3$) (*exists as rotamers*) δ 5.91 (ddd, *J* = 17.3, 10.3, 7.2 Hz, 1H), 5.33 (d, *J* = 17.1 Hz, 1H), 5.22 (d, *J* = 10.3 Hz, 1H), 4.31 – 4.16 (m, 1H), 3.73 – 3.52 (m, 1H), 1.68 – 1.56 (m, 5H), 1.50 (s, 3H), 1.47 (s, 9H), 1.33 – 1.25 (m, 2H), 0.93 (t, *J* = 7.3 Hz, 3H); ^{13}C NMR (151 MHz, $CDCl_3$) (*exists as rotamers*) (*exists as rotamers*) δ 152.38, 139.02,

117.21, 94.83, 94.16, 79.80, 77.55, 65.88, 65.39, 36.64, 35.07, 28.68, 28.42, 27.15, 26.40, 14.08, 13.67, 12.33; HR-ESIMS m/z: calculated for $C_{16}H_{29}NO_3Na^+$ $[M+Na]^+$: 306.2040, found 306.2042.

Compound **8a** :

$[\alpha]_D^{25}$ +5.2 (c 0.27, H_2O); ^1H NMR (600 MHz, D_2O) δ 4.53 (d, *J* = 2.9 Hz, 1H), 3.53 (dd, *J* = 6.9, 2.8 Hz, 1H), 1.88 – 1.80 (m, 1H), 1.58 – 1.50 (m, 1H), 1.29 – 1.22 (m, 1H), 1.01 (d, *J* = 6.9 Hz, 3H), 0.92 (t, *J* = 7.4 Hz, 3H); ^{13}C NMR (151 MHz, D_2O) δ 175.18, 67.51, 56.83, 34.56, 24.46, 14.20, 10.16; HR-ESIMS m/z: calculated for $C_7H_{16}NO_3^+$ $[M+H]^+$ (free amine): 162.1130, found 162.1132.

3) Preparation of (2R,3S)-3-amino-2-hydroxy-4-phenylbutanoic acid hydrochloride (8b)

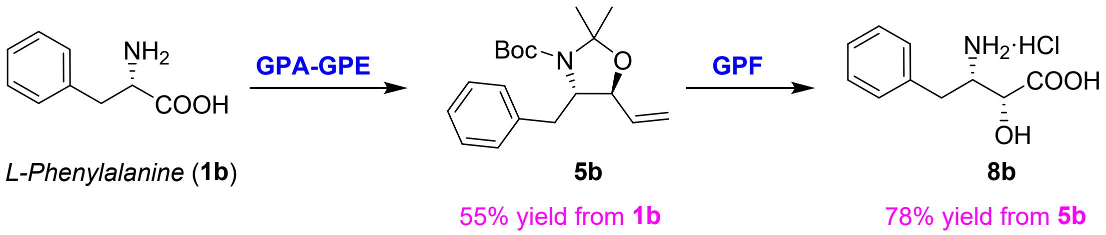

Compound **5b** :

$[\alpha]_D^{25}$ +8.2 (c 0.84, EtOAc); ^1H NMR (600 MHz, $CDCl_3$) (*exists as rotamers*) δ 7.33 – 7.18 (m, 5H), 5.83 – 5.62 (m, 1H), 5.16 (dd, *J* = 65.1, 23.3 Hz, 2H), 4.33 (s, 1H), 3.93 (s, 1H), 3.00 (d, *J* = 139.3 Hz, 2H), 1.67 – 1.57 (m, 3H), 1.56 (s, 9H), 1.36 (dd, *J* = 77.6, 73.7 Hz, 3H); ^{13}C NMR (151 MHz, $CDCl_3$) (*exists as rotamers*) δ 152.22, 151.74, 137.25, 136.47, 130.06, 129.58, 128.43, 128.23, 126.46, 117.97, 117.17, 94.78, 94.02, 80.00, 79.71, 78.73, 63.07, 62.85, 38.94, 36.17, 28.49, 28.27, 27.33, 26.54; HR-ESIMS m/z: calculated for $C_{19}H_{27}NO_3Na^+$ $[M+Na]^+$: 340.1883, found 340.1886.

Compound **8b** :

$[\alpha]_D^{25}$ -13.8 (c 0.61, H_2O); ^1H NMR (600 MHz, D_2O) δ 7.45 – 7.31 (m, 5H), 4.32 (d, *J* = 3.1 Hz, 1H), 3.93 (td, *J* = 7.7, 3.1 Hz, 1H), 3.13 (dd, *J* = 14.1, 7.4 Hz, 1H), 3.00 (dd, *J* = 14.1, 8.0 Hz, 1H).; ^{13}C NMR (151 MHz, D_2O) δ 174.42, 134.99, 129.44, 129.19, 127.74, 68.52, 54.52, 35.17; HR-ESIMS m/z: calculated for $C_{10}H_{14}NO_3^+$ $[M+H]^+$ (free amine): 196.0974, found 196.0975.

4) Preparation of (2R,3S)-3-amino-2-hydroxy-4-methylpentanoic acid hydrochloride (8c)

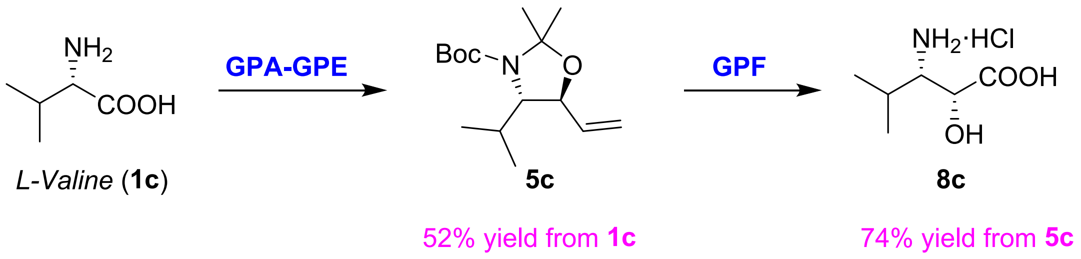

Compound **5c** :

$[\alpha]_D^{25}$ -8.2 (c 0.55, EtOAc); ^{1}H NMR (600 MHz, $CDCl_3$) (*exists as rotamers*) δ 5.91 (ddd, *J* = 17.3, 10.3, 7.2 Hz, 1H), 5.33 (d, *J* = 17.1 Hz, 1H), 5.22 (d, *J* = 10.3 Hz, 1H), 4.31 – 4.16 (m, 1H), 3.73 – 3.52 (m, 1H), 1.68 – 1.56 (m, 5H), 1.50 (s, 3H), 1.47 (s, 9H), 1.33 – 1.25 (m, 2H), 0.93 (t, *J* = 7.3 Hz, 3H); ^{13}C NMR (151 MHz, $CDCl_3$) (*exists as rotamers*) δ 152.46, 139.04, 116.94, 94.23, 79.72, 78.03, 66.75, 30.07, 28.72, 28.35, 27.28, 19.13, 17.14; HR-ESIMS m/z: calculated for $C_{15}H_{27}NO_3Na^+$ $[M+Na]^+$: 292.1883, found 292.1885.

Compound **8c** :

$[\alpha]_D^{25}$ +15.6 (c 0.50, H_2O); ^{1}H NMR (600 MHz, D_2O) δ 4.54 (d, *J* = 3.0 Hz, 1H), 3.42 (dd, *J* = 7.4, 2.8 Hz, 1H), 2.07 (dd, *J* = 13.8, 6.9 Hz, 1H), 1.05 (d, *J* = 6.9 Hz, 6H); ^{13}C NMR (151 MHz, D_2O) δ 175.02, 67.91, 58.53, 27.96, 18.22, 17.51; HR-ESIMS m/z: calculated for $C_6H_{14}NO_3^+$ $[M+H]^+$ (free amine): 148.0974, found 148.0975.

5) Preparation of (2R,3S)-3-amino-2-hydroxy-3-phenylpropanoic acid hydrochloride (8d)

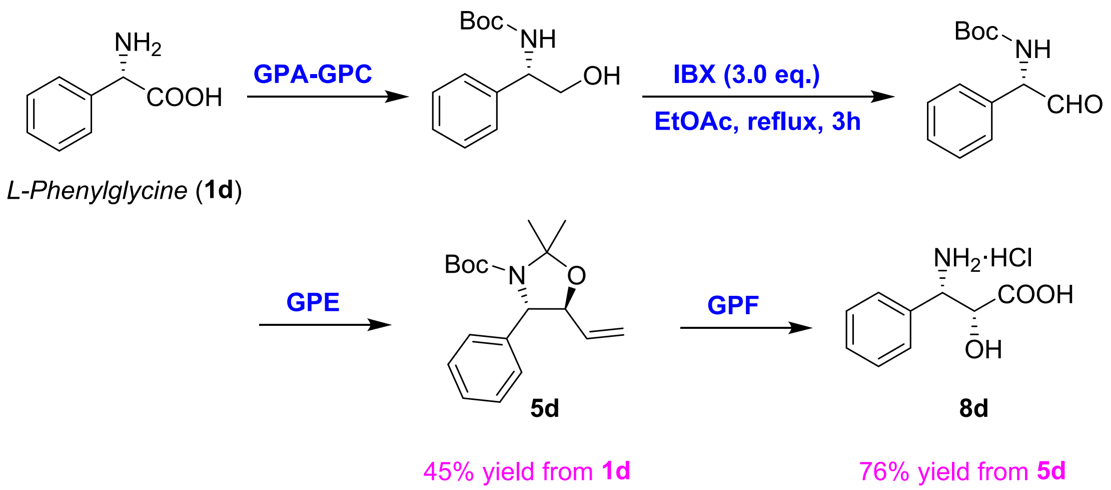

Compound **5d** :

$[\alpha]_D^{25}$ +47.5 (c 1.05, EtOAc); ^{1}H NMR (600 MHz, $CDCl_3$) (*exists as rotamers*) δ 7.36 – 7.21 (m, 5H), 5.89 (ddd, *J* = 17.1, 10.1, 7.1 Hz, 1H), 5.22 (dd, *J* = 29.5, 13.8 Hz, 2H), 4.37 (s, 1H), 4.27 (t, *J* = 7.1 Hz, 1H), 1.76 (d, *J* = 13.5 Hz, 6H), 1.47 – 1.02 (m, 9H); ^{13}C NMR (151 MHz, $CDCl_3$) (*exists as rotamers*) δ 151.83, 140.11, 133.78, 128.30, 127.25, 126.36, 119.26, 94.59, 83.72, 79.58, 67.29, 27.87, 26.34, 25.66, 25.62; HR-ESIMS m/z: calculated for $C_{18}H_{25}NO_3Na^+$ $[M+Na]^+$: 326.1727, found 326.1729.

Compound **8d** :

$[\alpha]_D^{25}$ -8.7 (c 0.38, H_2O); ^{1}H NMR (600 MHz, D_2O) δ 7.53 – 7.42 (m, 5H), 4.66 (d, *J* = 6.5 Hz, 1H), 4.62 (d, *J* = 6.5 Hz, 1H); ^{13}C NMR (151 MHz, D_2O) δ 173.63, 132.68, 129.84, 129.30, 127.45, 71.71, 56.94; HR-ESIMS m/z: calculated for $C_9H_{12}NO_3^+$ $[M+H]^+$ (free amine): 182.0817, found 182.0818.

6) Preparation of (2R,3S)-3-amino-4-cyclohexyl-2-hydroxybutanoic acid hydrochloride (8e)

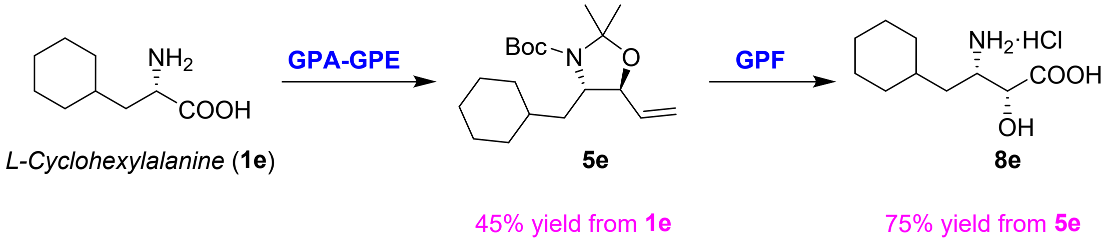

Compound **5e** :

$[\alpha]_D^{25}$ +7.8 (c 0.23, EtOAc); ^{1}H NMR (600 MHz, $CDCl_3$) (*exists as rotamers*) δ 6.02 – 5.83 (m, 1H), 5.30 (d, *J* = 17.2 Hz, 1H), 5.18 (d, *J* = 10.3 Hz, 1H), 4.25 (dd, *J* = 7.1, 3.6 Hz, 1H), 3.90 –

3.71 (m, 1H), 1.76 (d, J = 12.9 Hz, 1H), 1.68 – 1.56 (m, 8H), 1.49 (s, 3H), 1.46 (s, 9H), 1.29 – 1.10 (m, 5H), 0.99 – 0.87 (m, 2H); ^{13}C NMR (151 MHz, $CDCl_3$) (*exists as rotamers*) δ 151.71, 138.14, 132.75, 120.86, 117.30, 112.32, 109.63, 83.07, 81.79, 79.66, 75.11, 59.83, 57.70, 42.10, 37.17, 36.25, 34.92, 34.43, 33.80, 33.19, 32.29, 30.85, 29.64, 28.46, 28.31, 28.00, 27.38, 26.51, 26.41, 26.30, 26.13, 26.01; HR-ESIMS m/z: calculated for $C_{19}H_{33}NO_3Na^+$ $[M+Na]^+$: 346.2353, found 346.2356.

Compound **8e** :

$[\alpha]_D^{25}$ +365.6 (c 0.16, H_2O); ^{1}H NMR (600 MHz, D_2O) δ 4.40 (bs, 1H), 3.71 (bs, 1H), 1.75 – 1.58 (m, 6H), 1.56 – 1.48 (m, 1H), 1.38 (s, 1H), 1.27 – 1.10 (m, 3H), 1.02 – 0.86 (m, 2H).; ^{13}C NMR (151 MHz, D_2O) δ 174.59, 69.35, 50.90, 36.32, 32.92, 32.56, 32.17, 25.88, 25.53, 25.46; HR-ESIMS m/z: calculated for $C_{10}H_{20}NO_3^+$ $[M+H]^+$ (free amine): 202.1443, found 202.1444.

7) Preparation of (2R,3S)-3-amino-2-hydroxy-4,4-dimethylpentanoic acid hydrochloride (8f)

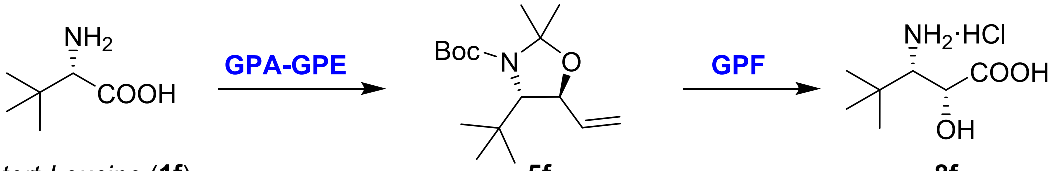

L-tert-Leucine (**1f**) **5f** **8f**

60% yield from **1f** 95% yield from **5f**

Compound **5f** :

$[\alpha]_D^{25}$ -25.6 (c 0.16, EtOAc); ^{1}H NMR (600 MHz, $CDCl_3$) δ 6.03 (ddd, J = 17.0, 10.4, 6.3 Hz, 1H), 5.24 (d, J = 17.2 Hz, 1H), 5.13 (d, J = 10.5 Hz, 1H), 4.45 (dd, J = 6.2, 1.3 Hz, 1H), 3.84 (d, J = 95.1 Hz, 1H), 1.57 (s, 6H), 1.45 (s, 9H), 0.92 (s, 9H); ^{13}C NMR (151 MHz, $CDCl_3$) δ 140.16, 115.86, 79.84, 78.71, 78.15, 69.22, 28.25, 28.01, 27.12; HR-ESIMS m/z: calculated for $C_{16}H_{29}NO_3Na^+$ $[M+Na]^+$: 306.2040, found 306.2044.

Compound **8f** :

$[\alpha]_D^{25}$ +194.8 (c 0.31, H_2O); ^{1}H NMR (600 MHz, D_2O) δ 4.56 (d, J = 1.2 Hz, 1H), 3.43 (s, 1H), 1.00 (s, 9H); ^{13}C NMR (151 MHz, D_2O) δ 175.57, 66.62, 60.59, 32.84, 25.75; HR-ESIMS m/z: calculated for $C_7H_{16}NO_3^+$ $[M+H]^+$ (free amine): 162.1130, found 162.1132.

8) Preparation of (2R,3S)-3-amino-2-hydroxy-5-methylhexanoic acid hydrochloride (8g)

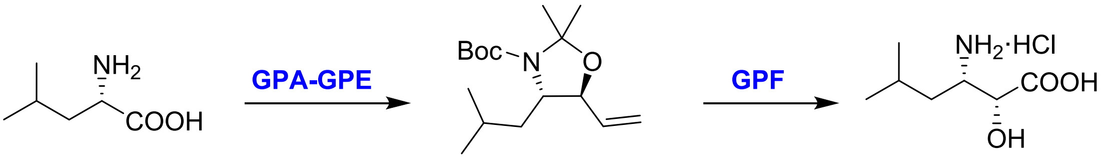

L-Leucine (**1g**) **5g** **8g**

50% yield from **1g** 95% yield from **5g**

Compound **5g** :

$[\alpha]_D^{25}$ +2.8 (c 0.39, EtOAc); ^{1}H NMR (600 MHz, $CDCl_3$) δ 5.94 (ddd, *J* = 17.3, 10.1, 7.4 Hz, 1H), 5.30 (d, *J* = 16.9 Hz, 1H), 5.18 (d, *J* = 10.3 Hz, 1H), 4.25 (dd, *J* = 7.2, 3.5 Hz, 1H), 3.85 – 3.67 (m, 1H), 1.57 (d, *J* = 11.0 Hz, 5H), 1.49 (s, 3H), 1.46 (s, 9H), 1.41 (s, 1H), 0.91 (dd, *J* = 8.9, 4.6 Hz, 6H); ^{13}C NMR (151 MHz, $CDCl_3$) δ 151.70, 138.15, 117.33, 94.26, 81.82, 79.71, 60.36, 43.55, 42.02, 28.46, 28.31, 27.48, 25.38, 23.96, 21.38; HR-ESIMS m/z: calculated for $C_{16}H_{29}NO_3Na^+$ $[M+Na]^+$: 306.2040, found 306.2043.

Compound **8g** :

$[\alpha]_D^{25}$ -3.7 (c 0.35, H_2O); ^{1}H NMR (600 MHz, D_2O) δ 4.41 (d, *J* = 3.4 Hz, 1H), 3.66 (td, *J* = 7.3, 3.4 Hz, 1H), 1.68 (dt, *J* = 13.6, 6.7 Hz, 1H), 1.60 (dt, *J* = 14.4, 7.2 Hz, 1H), 1.51 (dd, *J* = 14.1, 7.0 Hz, 1H), 0.91 (t, *J* = 6.3 Hz, 6H); ^{13}C NMR (151 MHz, D_2O) δ 174.39, 69.19, 51.54, 37.74, 23.72, 21.63, 21.26; HR-ESIMS m/z: calculated for $C_7H_{16}NO_3^+$ $[M+H]^+$ (free amine): 162.1130, found 162.1132.

9) Preparation of (2R,3S)-3-Amino-2-hydroxybutanoic acid hydrochloride (8h)

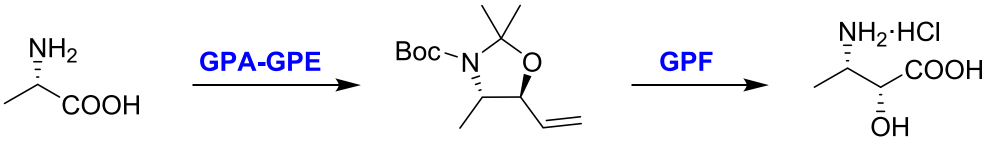

L-Alanine (**1h**) **5h** **8h**

45% yield from **1h** 85% yield from **5h**

Compound **5h** :

$[\alpha]_D^{25}$ +13.9 (c 0.31, EtOAc); ^{1}H NMR (600 MHz, $CDCl_3$) δ 5.83 (ddd, *J* = 17.4, 10.3, 7.3 Hz, 1H), 5.35 (dd, *J* = 17.2, 1.0 Hz, 1H), 5.24 (d, *J* = 10.3 Hz, 1H), 4.02 (t, *J* = 7.3 Hz, 1H), 3.48 (d, *J* =

41.4 Hz, 1H), 1.56 (d, J = 5.9 Hz, 3H), 1.48 (d, J = 10.0 Hz, 3H), 1.45 (s, 9H), 1.28 (d, J = 5.4 Hz, 3H); ^{13}C NMR (151 MHz, $CDCl_3$) δ 152.14, 138.03, 135.64, 132.89, 119.00, 118.78, 116.42, 94.24, 82.87, 79.65, 57.60, 30.84, 28.41, 28.30, 26.60, 26.21, 18.47, 17.18; HR-ESIMS m/z: calculated for $C_{13}H_{23}NO_3Na^+$ $[M+Na]^+$: 264.1570, found 264.1573.

Compound **8h** :

$[\alpha]_D^{25}$ +8.1 (c 0.91, H_2O); ^{1}H NMR (600 MHz, D_2O) δ 4.32 (d, J = 4.8 Hz, 1H), 3.70 – 3.65 (m, 1H), 1.32 (d, J = 6.8 Hz, 3H); ^{13}C NMR (151 MHz, D_2O) δ 173.10, 69.85, 48.45, 13.72; HR-ESIMS m/z: calculated for $C_4H_{10}NO_3^+$ $[M+H]^+$ (free amine): 120.0661, found 120.0662.

10) Preparation of (2R,3S)-3-amino-2-hydroxy-6-methylheptanoic acid hydrochloride (8i)

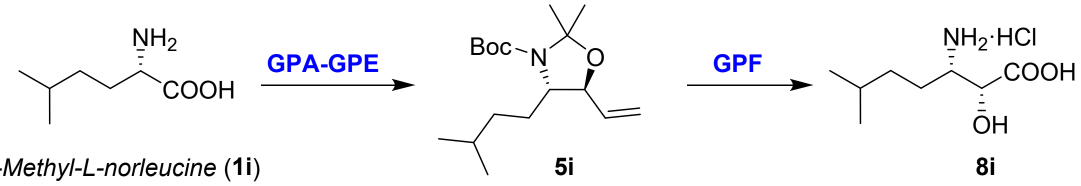

5-Methyl-L-norleucine (**1i**) **5i** **8i**

50% yield from **1i** 84% yield from **5i**

Compound **5i** :

$[\alpha]_D^{25}$ +24.0 (c 0.35, EtOAc); ^{1}H NMR (600 MHz, $CDCl_3$) δ 5.89 (ddd, J = 17.3, 10.3, 7.2 Hz, 1H), 5.31 (d, J = 17.2 Hz, 1H), 5.20 (d, J = 10.3 Hz, 1H), 4.25 – 4.18 (m, 1H), 3.60 (d, J = 54.1 Hz, 1H), 1.72 (s, 1H), 1.68 – 1.63 (m, 1H), 1.58 (s, 3H), 1.53 – 1.51 (m, 1H), 1.49 (s, 3H), 1.45 (s, 9H), 1.13 (s, 2H), 0.89 – 0.85 (m, 6H); ^{13}C NMR (151 MHz, $CDCl_3$) δ 151.90, 137.54, 132.77, 117.81, 80.76, 79.56, 61.92, 34.18, 28.41, 28.31, 28.06, 22.63, 22.49; HR-ESIMS m/z: calculated for $C_{17}H_{31}NO_3Na^+$ $[M+Na]^+$: 320.2196, found 320.2198.

Compound **8i** :

$[\alpha]_D^{25}$ -3.9 (c 0.92, H_2O); ^{1}H NMR (600 MHz, D_2O) δ 4.41 (d, J = 3.7 Hz, 1H), 3.56 (td, J = 6.9, 3.9 Hz, 1H), 1.81 – 1.73 (m, 1H), 1.67 – 1.60 (m, 1H), 1.56 (dt, J = 13.3, 6.7 Hz, 1H), 1.28 (qd, J = 13.0, 6.5 Hz, 2H), 0.86 (dd, J = 6.6, 2.6 Hz, 6H); ^{13}C NMR (151 MHz, D_2O) δ 174.57, 69.19, 53.62, 33.54, 27.11, 26.85, 21.60, 21.57; HR-ESIMS m/z: calculated for $C_8H_{18}NO_3^+$ $[M+H]^+$ (free amine): 176.1287, found 176.1288.

11) Preparation of (2R,3S)-3-amino-2-hydroxydecanoic acid hydrochloride (8j)

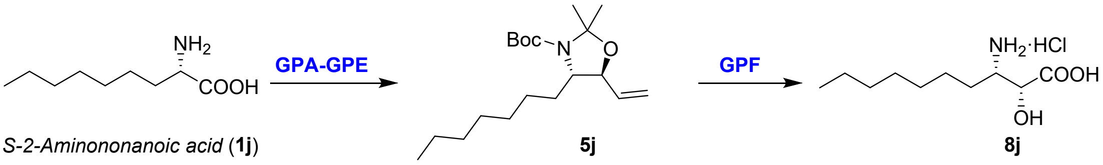

S-2-Aminononanoic acid (**1j**) **5j** **8j**

55% yield from **1j** 85% yield from **5j**

Compound **5j** :

$[\alpha]^{25}_{D}$ +13.8 (c 0.26, EtOAc); ^{1}H NMR (600 MHz, $CDCl_3$) δ 5.89 (ddd, *J* = 17.3, 10.3, 7.2 Hz, 1H), 5.31 (d, *J* = 17.1 Hz, 1H), 5.20 (d, *J* = 10.3 Hz, 1H), 4.26 – 4.17 (m, 1H), 3.78 – 3.56 (m, 1H), 1.65 (d, *J* = 6.4 Hz, 1H), 1.58 (s, 3H), 1.48 (s, 3H), 1.45 (s, 9H), 1.42 (s, 1H), 1.27 (d, *J* = 6.6 Hz, 10H), 0.86 (d, *J* = 8.2 Hz, 3H); ^{13}C NMR (151 MHz, $CDCl_3$) δ 151.90, 137.53, 117.80, 94.24, 80.76, 79.57, 61.71, 31.71, 29.52, 29.19, 28.40, 25.83, 25.16, 22.56, 14.01; HR-ESIMS m/z: calculated for $C_{19}H_{35}NO_3Na^+$ $[M+Na]^+$: 348.2509, found 348.2511.

Compound **8j** :

$[\alpha]^{25}_{D}$ +7.1 (c 0.31, H_2O); ^{1}H NMR (600 MHz, D_2O) δ 4.42 (d, *J* = 3.4 Hz, 1H), 3.59 (s, 1H), 1.76 (dt, *J* = 15.6, 7.9 Hz, 1H), 1.68 – 1.62 (m, 1H), 1.45 – 1.25 (m, 10H), 0.83 (t, *J* = 6.3 Hz, 3H); ^{13}C NMR (151 MHz, D_2O) δ 174.41, 69.05, 53.29, 30.96, 28.88, 28.20, 28.07, 24.45, 21.98, 13.41; HR-ESIMS m/z: calculated for $C_{10}H_{22}NO_3^+$ $[M+H]^+$ (free amine): 204.1600, found 204.1601.

12) Preparation of (2R,3S)-3-amino-2-hydroxy-5-phenylpentanoic acid hydrochloride (8k)

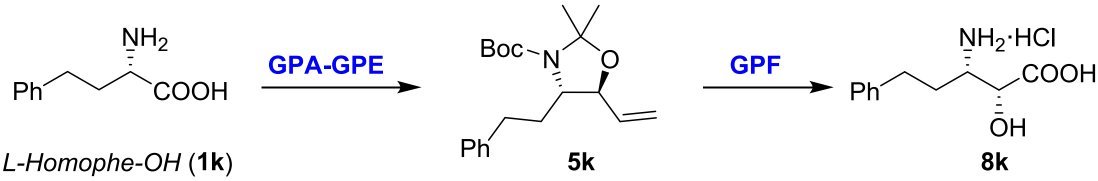

L-Homophe-OH (**1k**) **5k** **8k**

52% yield from **1k** 85% yield from **5k**

Compound **5k** :

$[\alpha]^{25}_{D}$ +27.1 (c 0.34, EtOAc); ^{1}H NMR (600 MHz, $CDCl_3$) δ 7.31 – 7.17 (m, 5H), 5.94 (ddd, *J* = 17.2, 9.9, 7.5 Hz, 1H), 5.36 (d, *J* = 17.1 Hz, 1H), 5.26 (d, *J* = 10.3 Hz, 1H), 4.35 (d, *J* = 5.5 Hz, 1H), 3.74 (d, *J* = 57.9 Hz, 1H), 2.67 – 2.51 (m, 2H), 2.20 – 1.93 (m, 2H), 1.63 (s, 3H), 1.54 (s, 3H),

1.48 (s, 9H); ^{13}C NMR (151 MHz, $CDCl_3$) δ 151.88, 141.46, 137.32, 132.59, 128.38, 128.28, 125.88, 118.12, 94.51, 93.94, 80.79, 79.77, 61.63, 61.20, 34.53, 33.41, 31.70, 28.40, 27.20, 26.82; HR-ESIMS m/z: calculated for $C_{20}H_{29}NO_3Na^+$ $[M+Na]^+$: 354.2040, found 354.2042.

Compound **8k** :

$[\alpha]_D^{25}$ -1.8 (c 0.33, H_2O); ^{1}H NMR (600 MHz, D_2O) δ 7.37 – 7.26 (m, 5H), 4.45 (d, J = 3.5 Hz, 1H), 3.61 (td, J = 6.9, 3.5 Hz, 1H), 2.76 (t, J = 8.0 Hz, 2H), 2.09 (dt, J = 15.2, 7.5 Hz, 1H), 1.96 (dd, J = 15.0, 6.7 Hz, 1H); ^{13}C NMR (151 MHz, D_2O) δ 174.48, 140.42, 128.91, 128.50, 126.62, 68.93, 52.83, 30.80, 30.71; HR-ESIMS m/z: calculated for $C_{11}H_{16}NO_3^+$ $[M+H]^+$ (free amine): 210.1130, found 210.1132.

13) Preparation of (2S,3R)-3-amino-2-hydroxy-3-phenylpropanoic acid hydrochloride (8l)

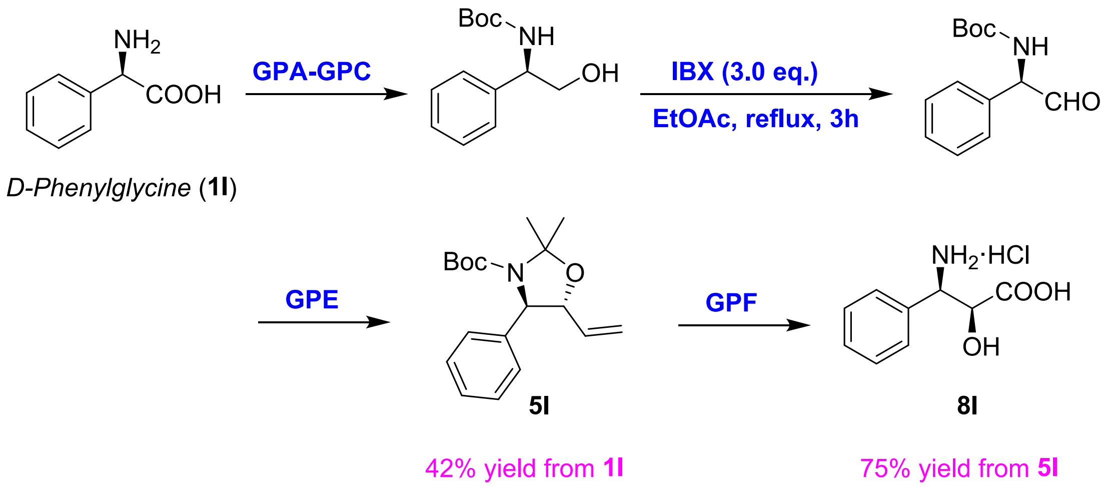

Compound **5l** :

$[\alpha]_D^{25}$ -46.0 (c 1.0, EtOAc); The spectral data for **5l** (^{1}HNMR, ^{13}CNMR and HMRS) was identical with those for **5d**.

Compound **8l** :

$[\alpha]_D^{25}$ +9.0 (c 0.45, H_2O); The spectral data for **8l** (^{1}HNMR, ^{13}CNMR and HMRS) was identical with those for **8d**.

14) Preparation of (2S,3R)-3-amino-2-hydroxy-5-methylhexanoic acid hydrochloride (8m)

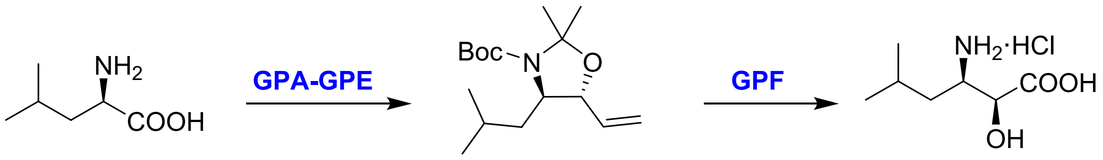

D-Leucine (**1m**) **5m** **8m**

48% yield from **1m** 92% yield from **5m**

Compound **5m** :

$[\alpha]_D^{25}$ -3.0 (c 0.45, EtOAc); The spectral data for **5m** (^{1}HNMR, ^{13}CNMR and HMRS) was identical with those for **5g**.

Compound **8m** :

$[\alpha]_D^{25}$ +3.8 (c 0.4, H_2O); The spectral data for **8m** (^{1}HNMR, ^{13}CNMR and HMRS) was identical with those for **8g**.

15) Preparation of (2S,3R)-3-amino-2-hydroxydecanoic acid hydrochloride (8n)

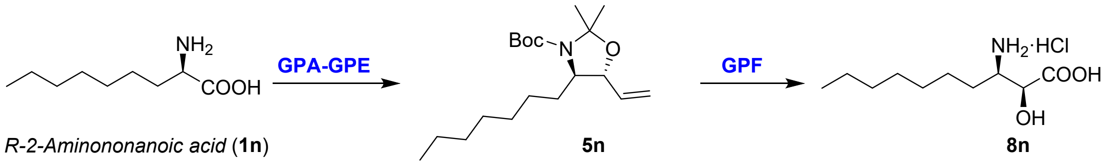

R-2-Aminononanoic acid (**1n**) **5n** **8n**

54% yield from **1n** 83% yield from **5n**

Compound **5n** :

$[\alpha]_D^{25}$ -14.2 (c 0.3, EtOAc); The spectral data for **5n** (^{1}HNMR, ^{13}CNMR and HMRS) was identical with those for **5j**.

Compound **8n** :

$[\alpha]_D^{25}$ -7.4 (c 0.4, H_2O); The spectral data for **8n** (^{1}HNMR, ^{13}CNMR and HMRS) was identical with those for **8j**.

16) Preparation of (2S,3R)-3-amino-2-hydroxy-4-phenylbutanoic acid hydrochloride (8o)

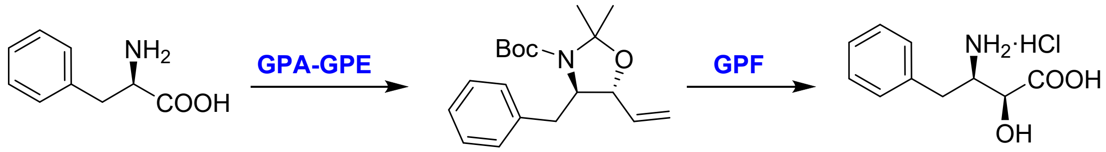

D-Phenylalanine (**1o**) **5o** **8o**

52% yield from **1o** 80% yield from **5o**

Compound **5o** :

$[\alpha]_D^{25}$ -8.0 (c 0.75, EtOAc); The spectral data for **5o** (^{1}HNMR, ^{13}CNMR and HMRS) was identical with those for **5b**.

Compound **8o** :

$[\alpha]_D^{25}$ +13.2 (c 0.6, H_2O); The spectral data for **8o** (^{1}HNMR, ^{13}CNMR and HMRS) was identical with those for **8b**.

17) Preparation of (2S,3R)-3-Amino-2-hydroxybutanoic acid hydrochloride (8p)

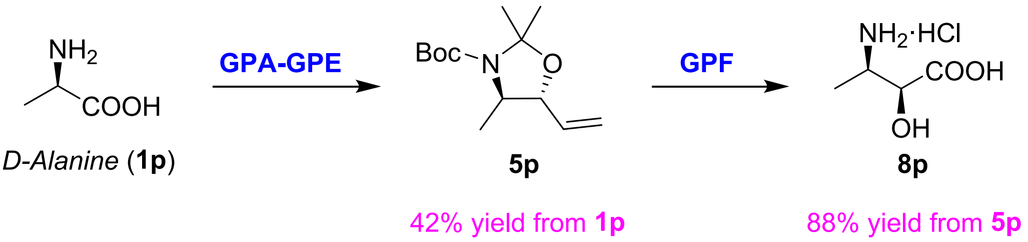

Compound **5p** :

$[\alpha]_D^{25}$ -14.3 (c 0.4, EtOAc); The spectral data for **5p** (^{1}HNMR, ^{13}CNMR and HMRS) was identical with those for **5h**.

Compound **8p** :

$[\alpha]_D^{25}$ -8.5 (c 1.0, H_2O); The spectral data for **8p** (^{1}HNMR, ^{13}CNMR and HMRS) was identical with those for **8h**.

18) Preparation of methyl (2S,3S)-3-((tert-butoxycarbonyl)amino)-2-hydroxyhexanoate (11)

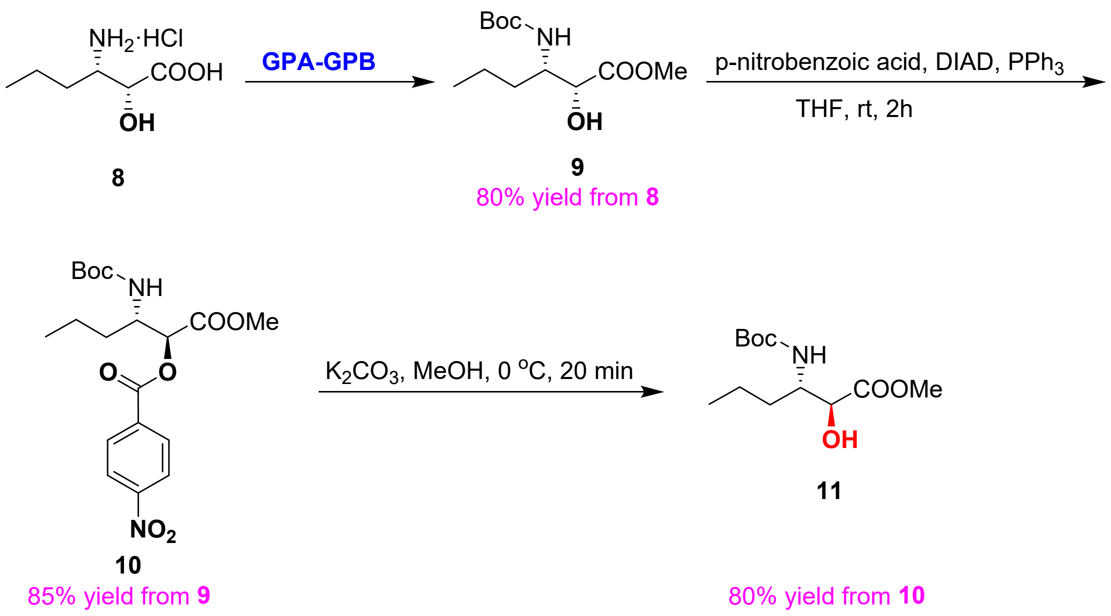

Compound **9** :

$[\alpha]_D^{25}$ -26.9 (c 0.58, EtOAc); ^{1}H NMR (600 MHz, $CDCl_3$) δ 4.72 (d, *J* = 9.5 Hz, 1H), 4.55 (s, 1H), 4.15 (d, *J* = 4.7 Hz, 1H), 3.99 (dd, *J* = 16.1, 8.3 Hz, 1H), 3.77 (s, 3H), 3.25 (d, *J* = 5.2 Hz, 1H), 1.56 (dd, *J* = 15.0, 7.4 Hz, 2H), 1.39 (s, 9H), 1.35 – 1.25 (m, 2H), 0.93 (t, *J* = 7.3 Hz, 3H); ^{13}C NMR (151 MHz, $CDCl_3$) δ 174.28, 155.40, 79.57, 79.26, 72.04, 52.61, 34.12, 28.16, 19.23, 13.68; HR-ESIMS m/z: calculated for $C_{12}H_{23}NO_5Na^+$ $[M+Na]^+$: 284.1468, found 284.1470.

To a solution of compound **9** (2.6 g, 9.95 mmol) in dry THF (100 mL) was added successively p-nitrobenzoic acid (2.5 g, 15 mmol), PPh_3 (5.25 g, 20 mmol) and DIAD (4.0 mL, 20 mmol) at 0 °C under N_2 atmosphere. After being stirred at room temperature for 2 h, volatiles of the reaction mixture were removed in vacuo. The residue was purified by silica gel column chromatography (EA/PE = 1/10) to afford compound **10** as an oil (3.47 g, 85%).

Compound **10** :

$[\alpha]_D^{25}$ -23.0 (c 0.82, EtOAc); ^{1}H NMR (600 MHz, $CDCl_3$) δ 8.26 (dd, *J* = 34.2, 8.4 Hz, 4H), 5.41 (d, *J* = 2.6 Hz, 1H), 4.64 (d, *J* = 9.2 Hz, 1H), 4.39 – 4.20 (m, 1H), 3.80 (s, 3H), 1.58 – 1.46 (m, 4H), 1.43 (s, 9H), 0.95 (t, *J* = 7.1 Hz, 3H); ^{13}C NMR (151 MHz, $CDCl_3$) δ 168.11, 163.92, 155.32, 150.79, 134.62, 131.07, 123.58, 79.96, 75.52, 52.65, 50.91, 32.53, 28.27, 19.16, 13.70; HR-ESIMS m/z: calculated for $C_{19}H_{26}N_2O_8Na^+$ $[M+Na]^+$: 433.1581, found 433.1582.

To a solution of compound **10** (1.36 g, 3.3 mmol) in MeOH (50 mL) was added K_2CO_3 (0.69 g, 5.0 mmol) at 0 °C. After being stirred at 0 °C for 20 min, the reaction was quenched with saturated aqueous NH_4Cl (100 mL) and the methanol was removed under vacuum. The residue was then diluted with water (200 mL). The aqueous phase was extracted with ethyl acetate for 3 times (250 mL×3). The combined organic phase was washed by saturated aqueous $NaHCO_3$ solution (200 mL), brine (300 mL), dried over sodium sulfate (anhydrous) and concentrated in *vacuo*. The residue was purified by silica gel column chromatography (EA/PE = 1/4) to afford compound **11** as a white solid (0.69 g, 80%).

Compound **11** :

$[\alpha]_D^{25}$ -11.7 (c 0.60, EtOAc); ^{1}H NMR (600 MHz, $CDCl_3$) δ 4.81 (d, *J* = 8.8 Hz, 1H), 4.30 (s, 1H), 3.96 (t, *J* = 9.5 Hz, 1H), 3.77 (s, 3H), 3.32 (d, *J* = 5.4 Hz, 1H), 1.43 (d, *J* = 14.5 Hz, 11H), 1.31 – 1.21 (m, 2H), 0.88 (t, *J* = 7.2 Hz, 3H); ^{13}C NMR (151 MHz, $CDCl_3$) δ 173.46, 155.84, 79.57, 73.39, 52.96, 52.59, 31.43, 28.28, 28.19, 19.04, 13.78; HR-ESIMS m/z: calculated for $C_{12}H_{23}NO_5Na^+$ $[M+Na]^+$: 284.1468, found 284.1470.

19) Preparation of L-(-)-threo-3-Hydroxyaspartic acid hydrochloride (10)

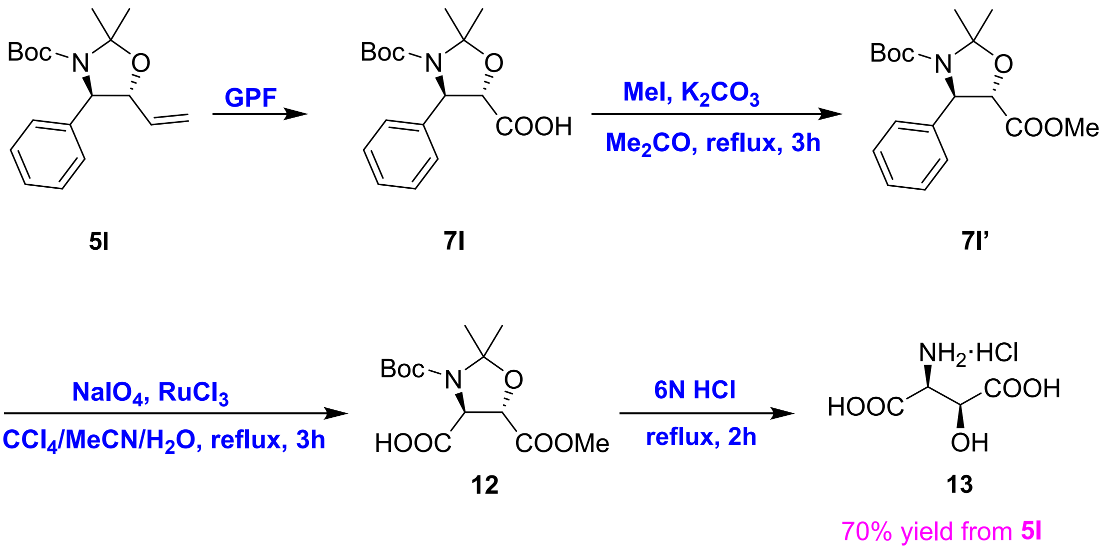

To a solution of **7l** (10 mmol, *1.0 eq.*) in dry acetone (100 mL) was added K_2CO_3 (4.2 g, 30 mmol, *3.0 eq.*) and MeI (1.3 mL, 20 mmol, *2.0 eq.*). After 15 min, the resultant mixture was

heated to reflux and stirred for 3h. The solution was cooled to room temperature and the solid was removed by filtration through a pad of celite and washed with EtOAc (100 mL). The total filtrate was concentrated in vacuo to give **7l'**, which was used for next step immediately and directly.

To a solution of the above-obtained **7l'** in CCl_4/MeCN/H_2O (1/1/2) (300 mL)was added $NaIO_4$ (64.8 g, 300 mmol, *30 eq.*) and $RuCl_3$ (0.21 g, 1 mmol, *0.1 eq.*). After 15 min, the resultant mixture was heated to reflux and stirred for 3h. The solution was cooled to room temperature, then quenched with saturated aqueous solution of Na_2SO_3 (300 mL). Volatiles of the mixture were removed in vacuo. The solution was then diluted with water (200 mL) and adjusted to pH 2 by slow addition of solid $KHSO_4$. The aqueous phase was extracted with ethyl acetate for 3 times (3×400 mL). The combined organic phase was washed by brine (300 mL), dried over sodium sulfate (anhydrous) and concentrated in *vacuo* to give the compound **12**, which was used for next step immediately and directly.

The above-obtained compound **12** was suspended in 6N HCl (120 mL) and refluxed for 2h. The reaction mixture was cooled to room temperature, then diluted with water (200 mL). The aqueous phase was extracted with ethyl acetate for 2 times (2×400 mL) to remove any organic soluble impurities. The aqueous phase was then evaporated under reduced pressure. The residue was then co-evaporated with MeCN for 2 times (2×100 mL) to provide the desired compound **13** as a yellow solid (70% yield over 3 steps from **5l**).

Compound **13** :

$[\alpha]_D^{25}$ -7.0 (c 0.75, 5N HCl); ^1H NMR (600 MHz, D_2O) δ 4.86 (bs, 1H), 4.42 (bs, 1H); ^{13}C NMR (151 MHz, D_2O) δ 173.34, 169.80, 68.54, 55.36; HR-ESIMS m/z: calculated for $C_4H_8NO_5^+$ $[M+H]^+$ (free amine): 150.0402, found 150.0404.

20) Preparation of tert-butyl ((2R,3S)-1,2-dihydroxy-5-phenylpentan-3-yl)carbamate (15)

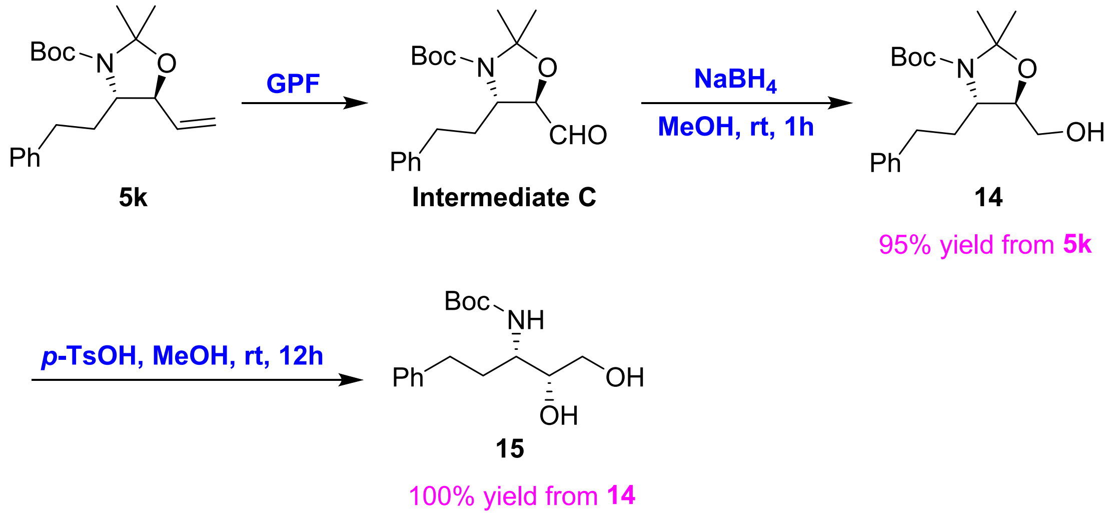

To a solution of **<u>Intermediate C</u>** (10 mmol, *1.0 eq.*) in MeOH (100 mL) was added $NaBH_4$ (1.2 g, 30 mmol, *3.0 eq.*) at 0 °C. After being stirred at room temperature for 1h, volatiles of the reaction mixture were removed in vacuo. The residue was then quenched with saturated aqueous NH_4Cl (200 mL). The aqueous phase was extracted with ethyl acetate for 3 times (3×300 mL). The combined organic phase was washed by brine (200 mL), dried over sodium sulfate (anhydrous) and concentrated in *vacuo* to give the compound **14** in essentially quantitative yield, which was used for next step immediately and directly. For analyses a small amount of this product was chromatographed on a SiO_2 column (EA/PE = 1/2).

Compound **14** :

$[\alpha]_D^{25}$ +10.8 (c 0.39, EtOAc); ^{1}H NMR (600 MHz, $CDCl_3$) δ 7.28 (t, *J* = 7.4 Hz, 2H), 7.19 (d, *J* = 6.8 Hz, 3H), 4.06 (dd, *J* = 10.8, 4.4 Hz, 1H), 3.76 (d, *J* = 45.6 Hz, 1H), 3.64 (dt, *J* = 16.0, 7.7 Hz, 2H), 2.60 (s, 2H), 2.00 (d, *J* = 44.1 Hz, 2H), 1.65 (d, *J* = 46.2 Hz, 6H), 1.46 (s, 9H); ^{13}C NMR (151 MHz, $CDCl_3$) δ 151.78, 141.10, 128.49, 128.29, 126.04, 94.50, 80.00, 63.93, 57.86, 35.15, 34.17, 32.03, 28.44, 27.57; HR-ESIMS m/z: calculated for $C_{19}H_{29}NO_4Na^+$ $[M+Na]^+$: 358.1989, found 358.1991.

To a solution of the above-obtained compound **14** (10 mmol, *1.0 eq.*) in MeOH (100 mL) was added *p*-TsOH (0.17 g, 1 mmol, 0.1 *eq.*). After being stirred at room temperature for 12h, the reaction mixture was directly quenched with excess Et_3N (0.28 mL, 2 mmol *0.2 eq.*), then

concentrated in *vacuo,* then purified by flash silica gel column chromatography (EA/PE=1/1) to afford the compound **15** in essentially quantitative yield.

Compound **15** :

$[\alpha]_D^{25}$ -25.8 (c 0.38, EtOAc); ^1H NMR (600 MHz, $CDCl_3$) δ 7.35 – 7.01 (m, 5H), 4.88 (d, *J* = 9.2 Hz, 1H), 3.81 – 3.57 (m, 2H), 3.60 – 3.31 (m, 3H), 2.92 (s, 1H), 2.77 – 2.62 (m, 2H), 1.97 – 1.73 (m, 2H), 1.45 (s, 9H); ^{13}C NMR (151 MHz, $CDCl_3$) δ 157.14, 141.44, 128.41, 128.34, 125.94, 79.99, 73.35, 63.62, 50.92, 38.58, 33.85, 32.52, 28.29; HR-ESIMS m/z: calculated for $C_{16}H_{25}NO_4Na^+$ $[M+Na]^+$: 318.1676, found 318.1677.

NMR Spectra of New Compounds and Selected Known Compounds

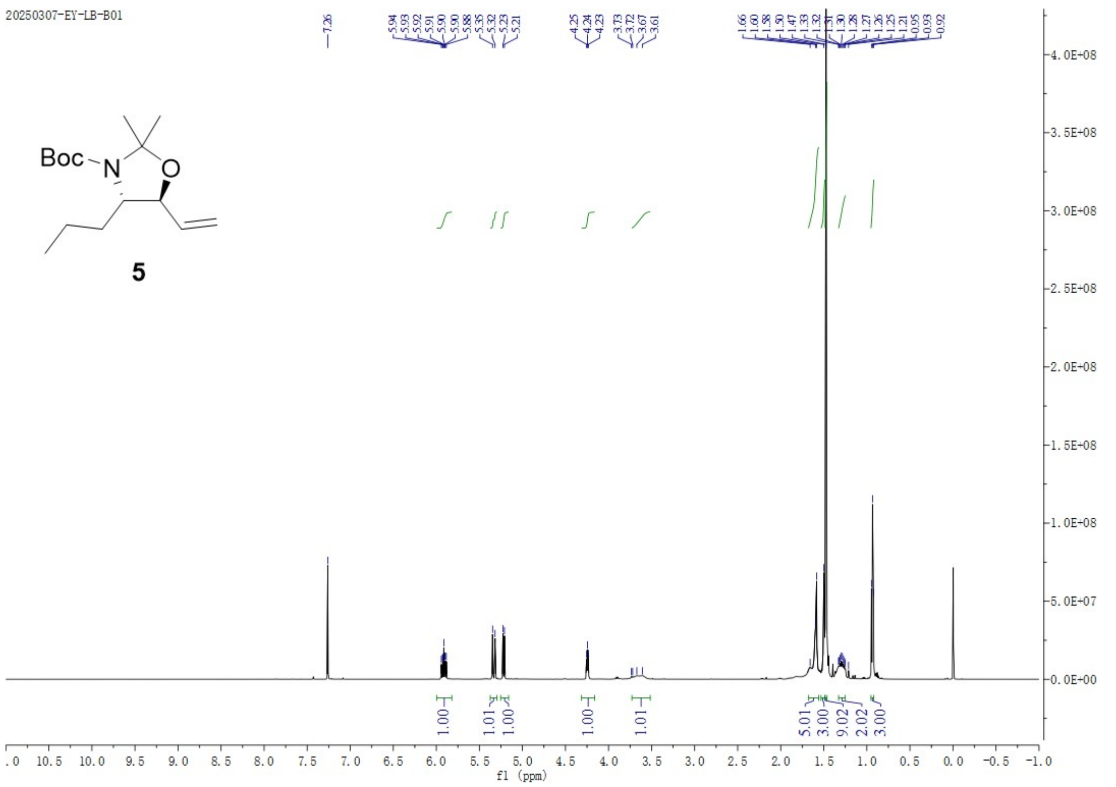

Figure S1. ^{1}H NMR of **5** ($CDCl_3$, 600 MHz) (*exists as rotamers*)

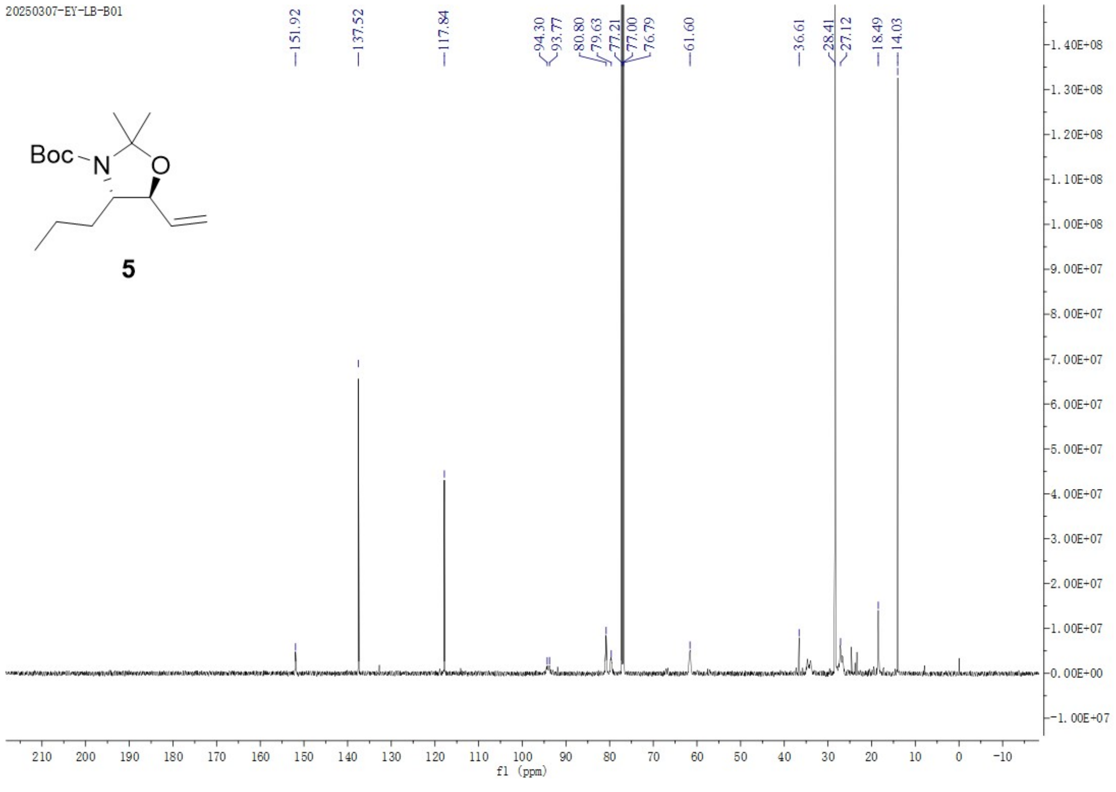

Figure S2. ^{13}C NMR of **5** ($CDCl_3$, 151 MHz) (*exists as rotamers*)

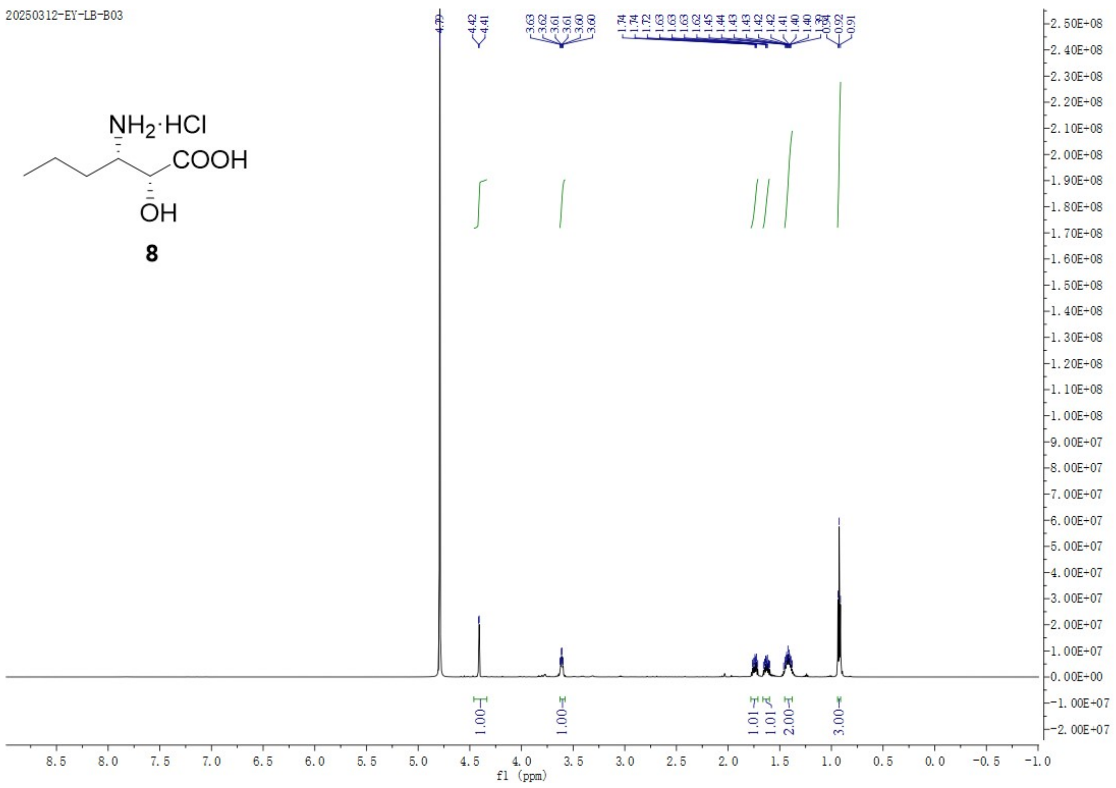

Figure S3. ^{1}H NMR of **8** (D_2O, 600 MHz)

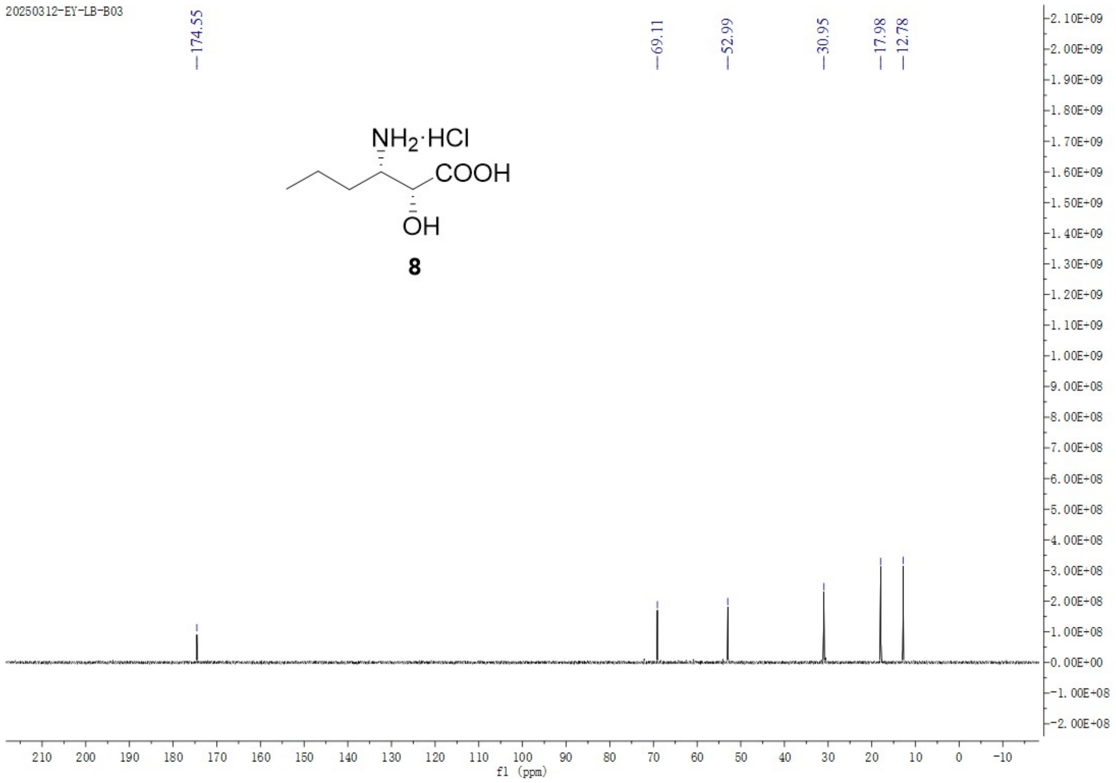

Figure S4. ^{13}C NMR of **8** (D_2O, 151 MHz)

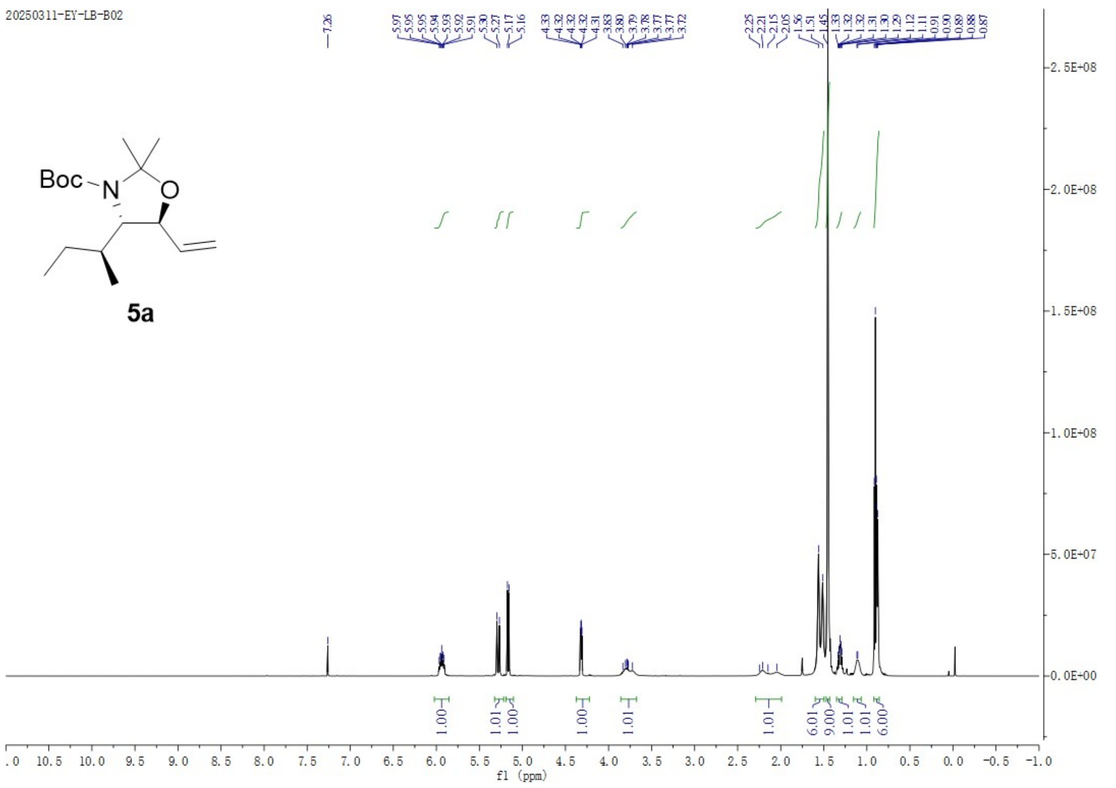

Figure S5. ^{1}H NMR of **5a** ($CDCl_3$, 600 MHz) (*exists as rotamers*)

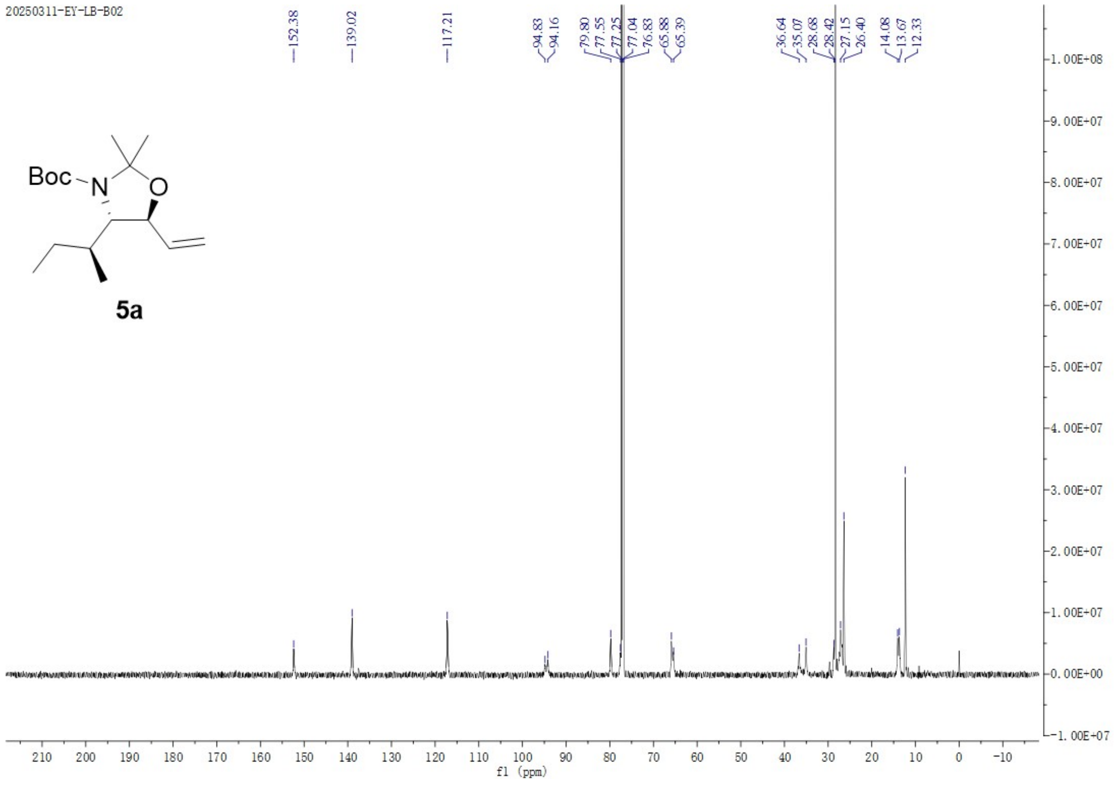

Figure S6. ^{13}C NMR of **5a** ($CDCl_3$, 151 MHz) (*exists as rotamers*)

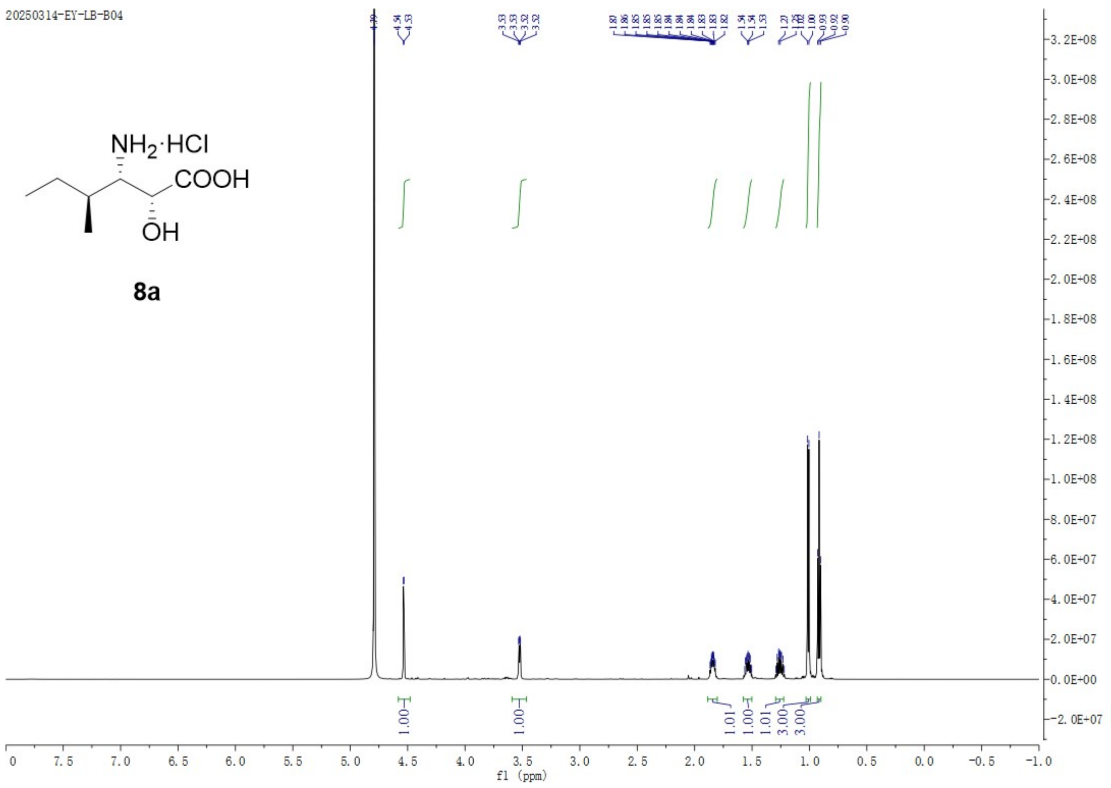

Figure S7. ^{1}H NMR of **8a** (D_2O, 600 MHz)

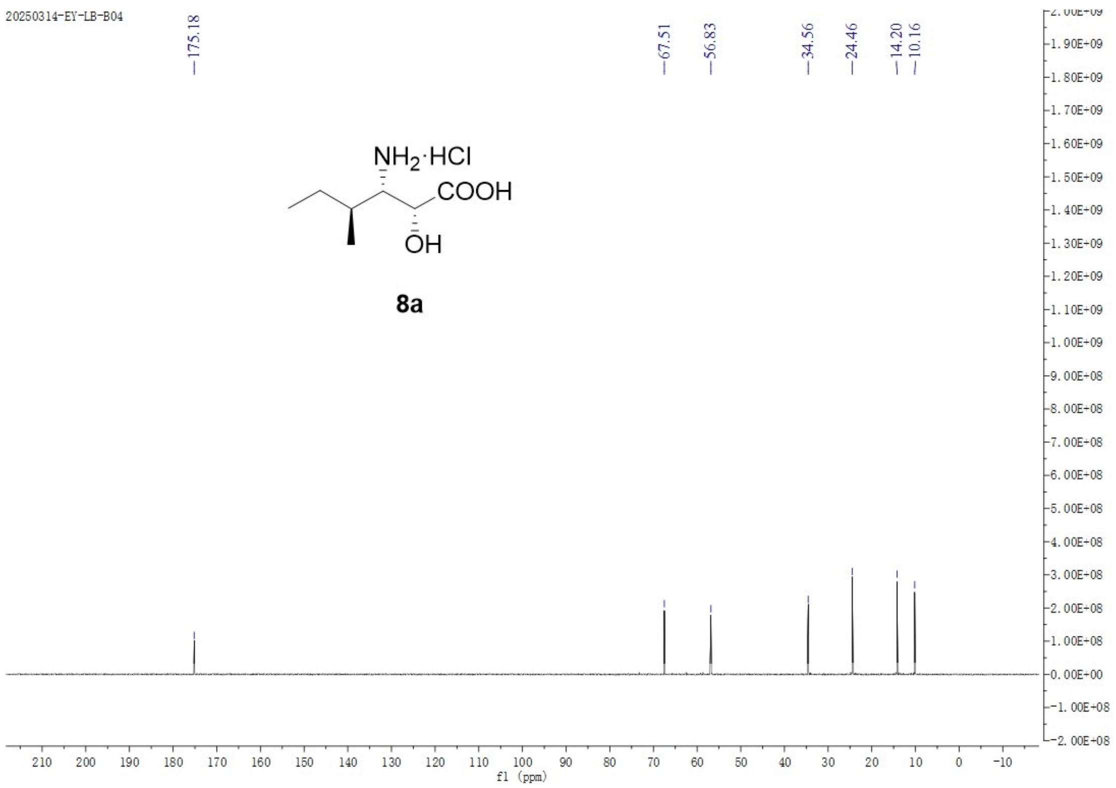

Figure S8. ^{13}C NMR of **8a** (D_2O, 151 MHz)

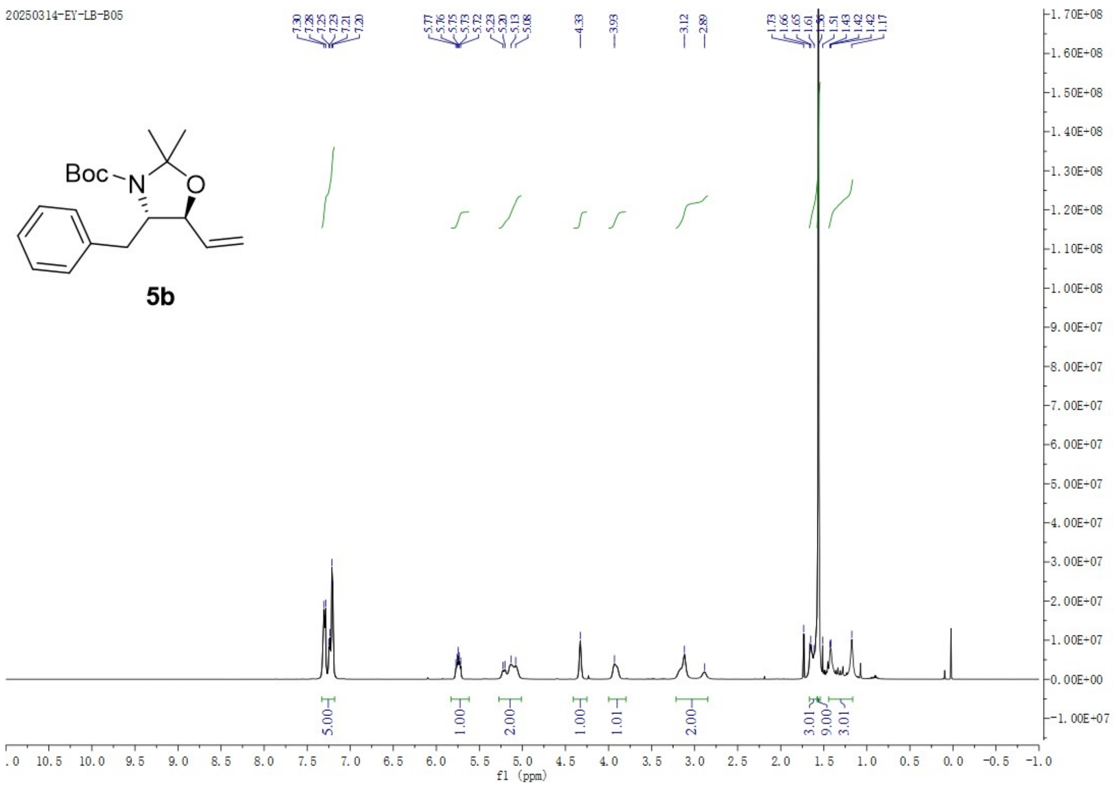

Figure S9. ^{1}H NMR of **5b** ($CDCl_3$, 600 MHz) (*exists as rotamers*)

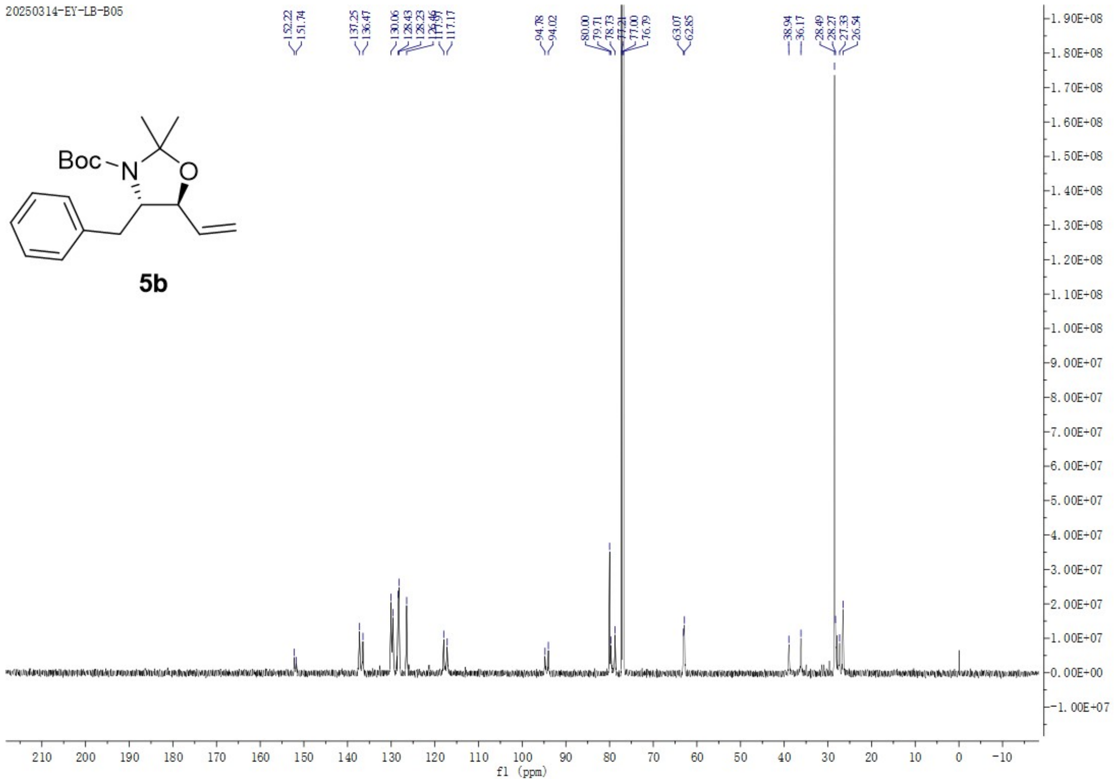

Figure S10. ^{13}C NMR of **5b** ($CDCl_3$, 151 MHz) (*exists as rotamers*)

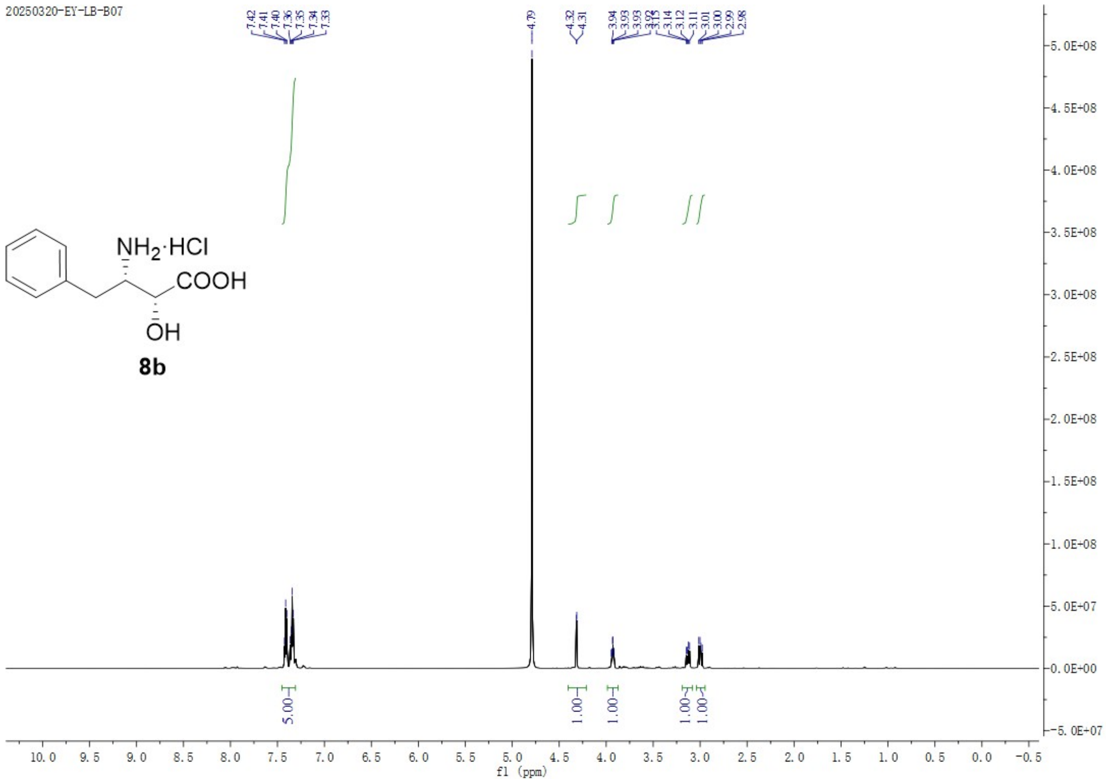

Figure S11. ^{1}H NMR of **8b** (D_2O, 600 MHz)

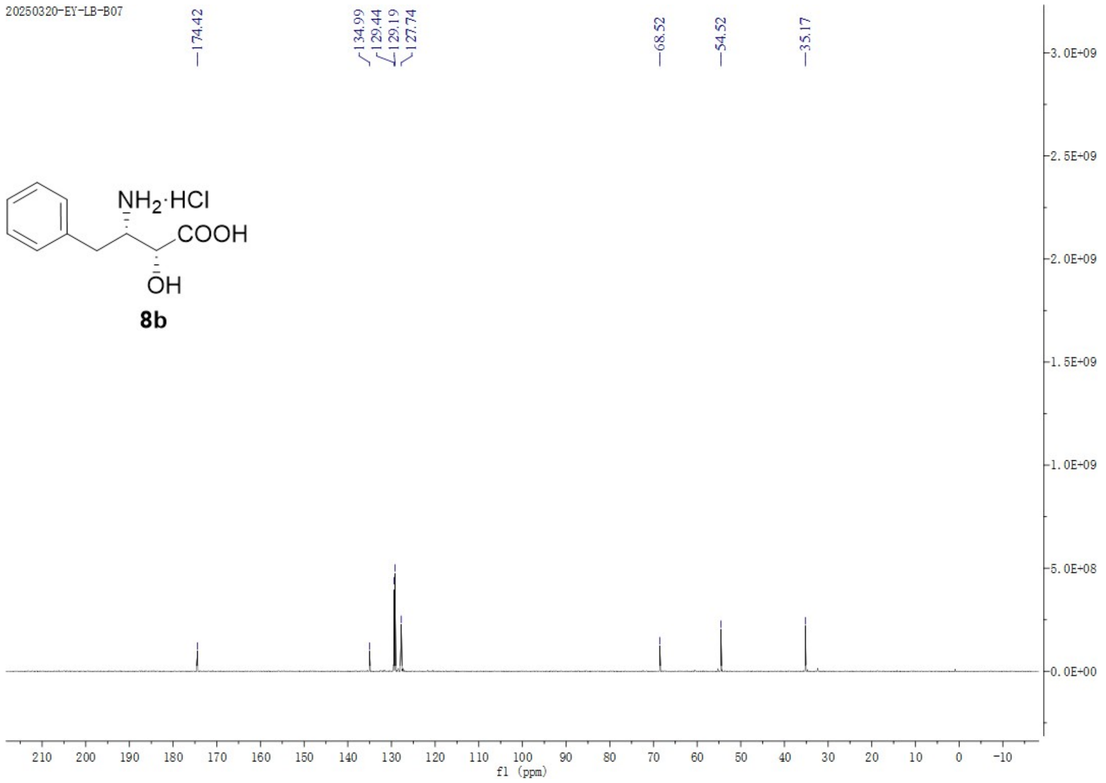

Figure S12. ^{13}C NMR of **8b** (D_2O, 151 MHz)

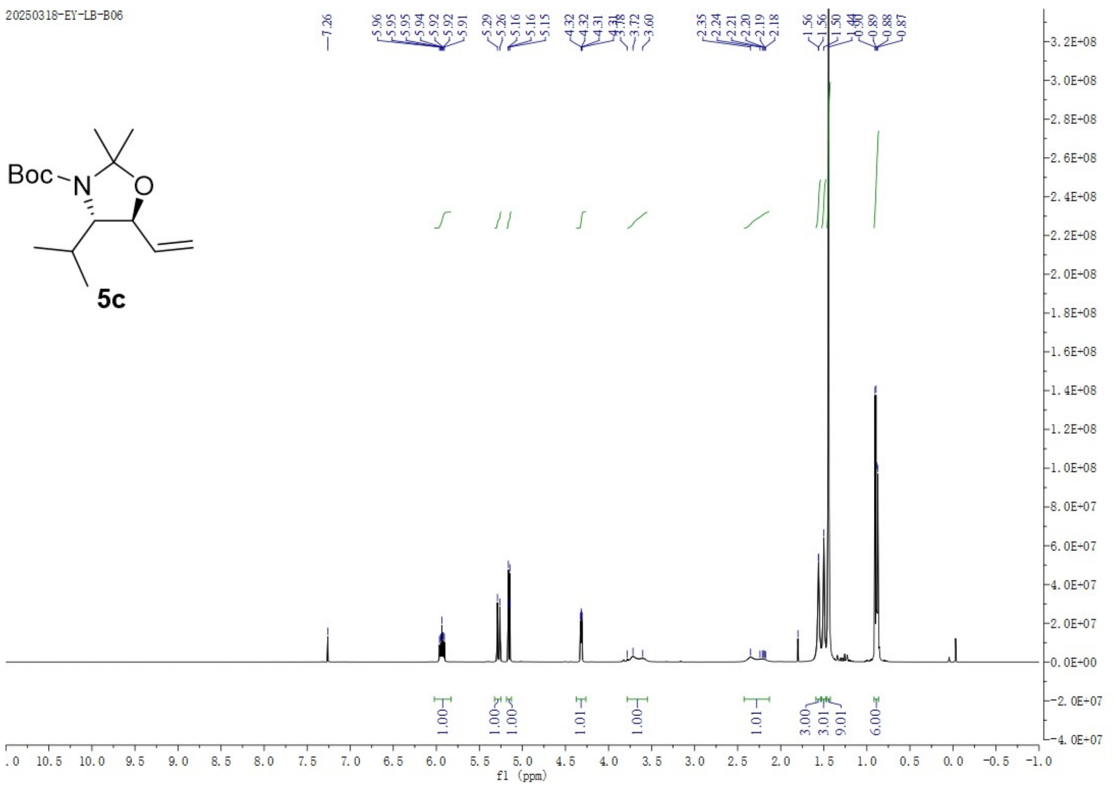

Figure S13. ^{1}H NMR of **5c** ($CDCl_3$, 600 MHz) (*exists as rotamers*)

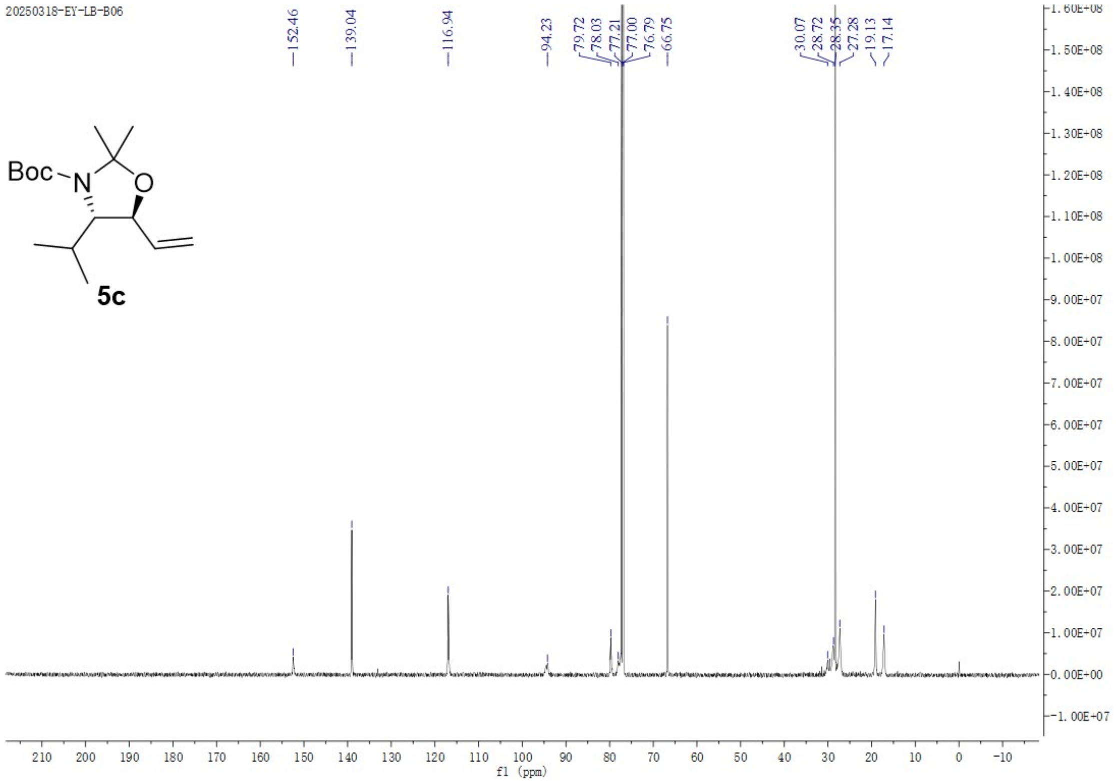

Figure S14. ^{13}C NMR of **5c** ($CDCl_3$, 151 MHz) (*exists as rotamers*)

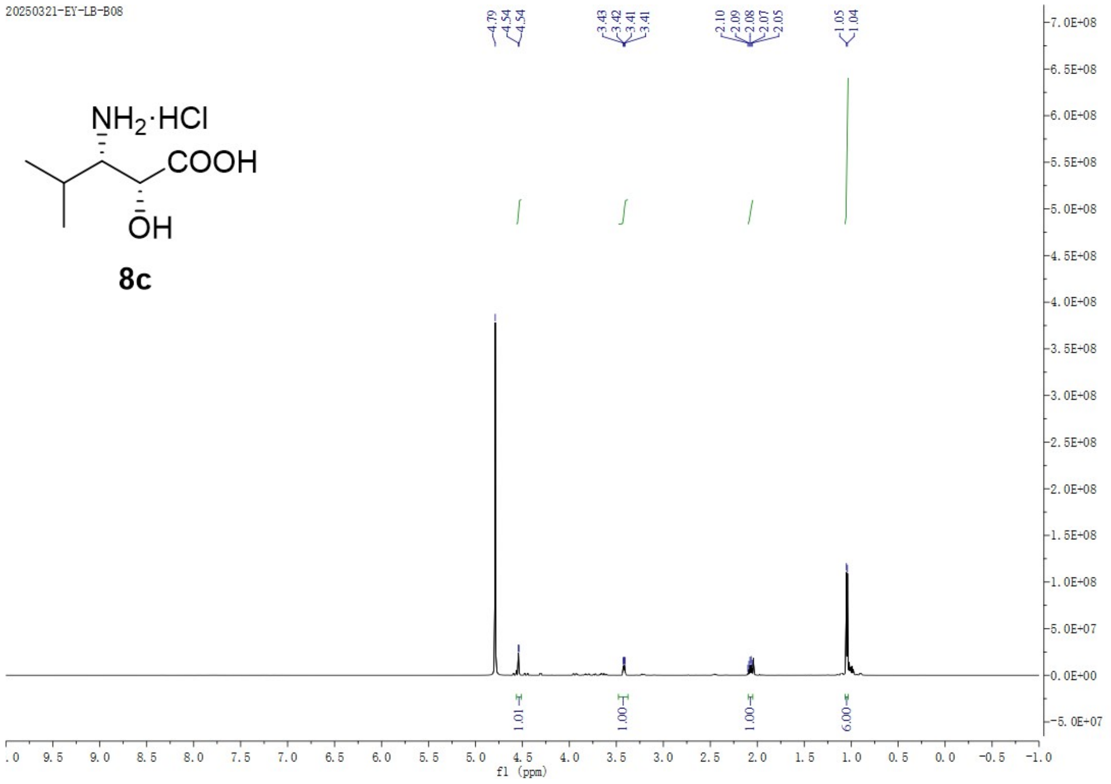

Figure S15. ^{1}H NMR of **8c** (D_2O, 600 MHz)

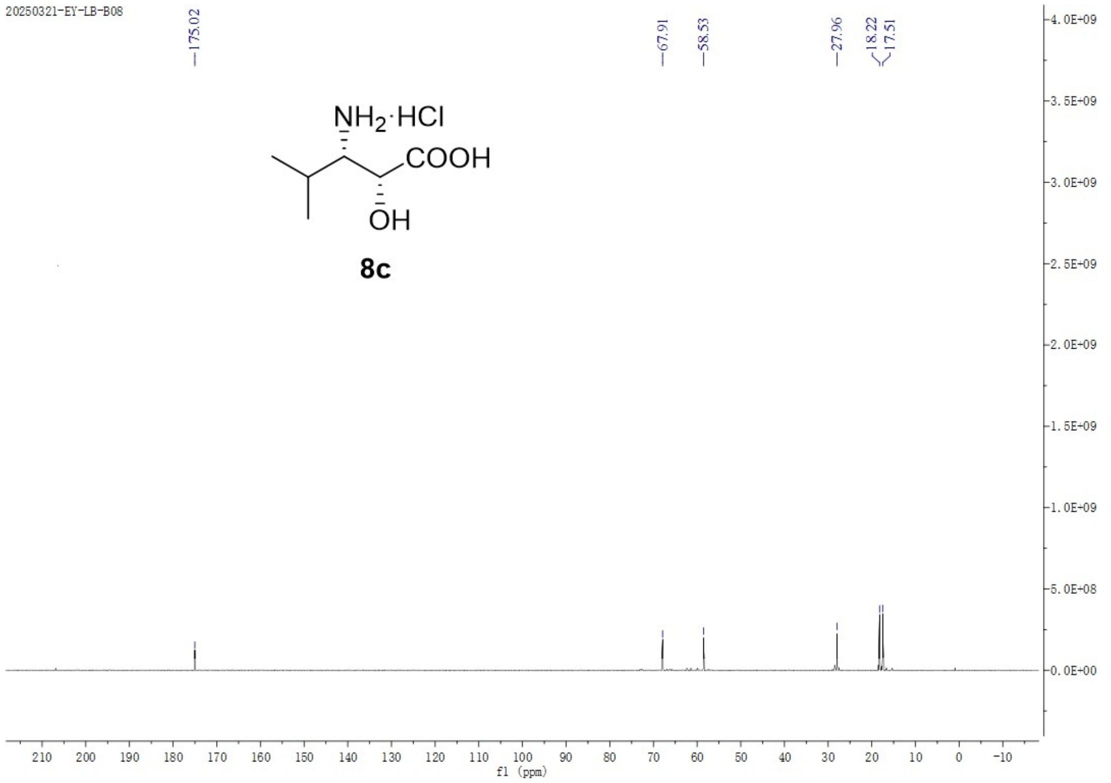

Figure S16. ^{13}C NMR of **8c** (D_2O, 151 MHz)

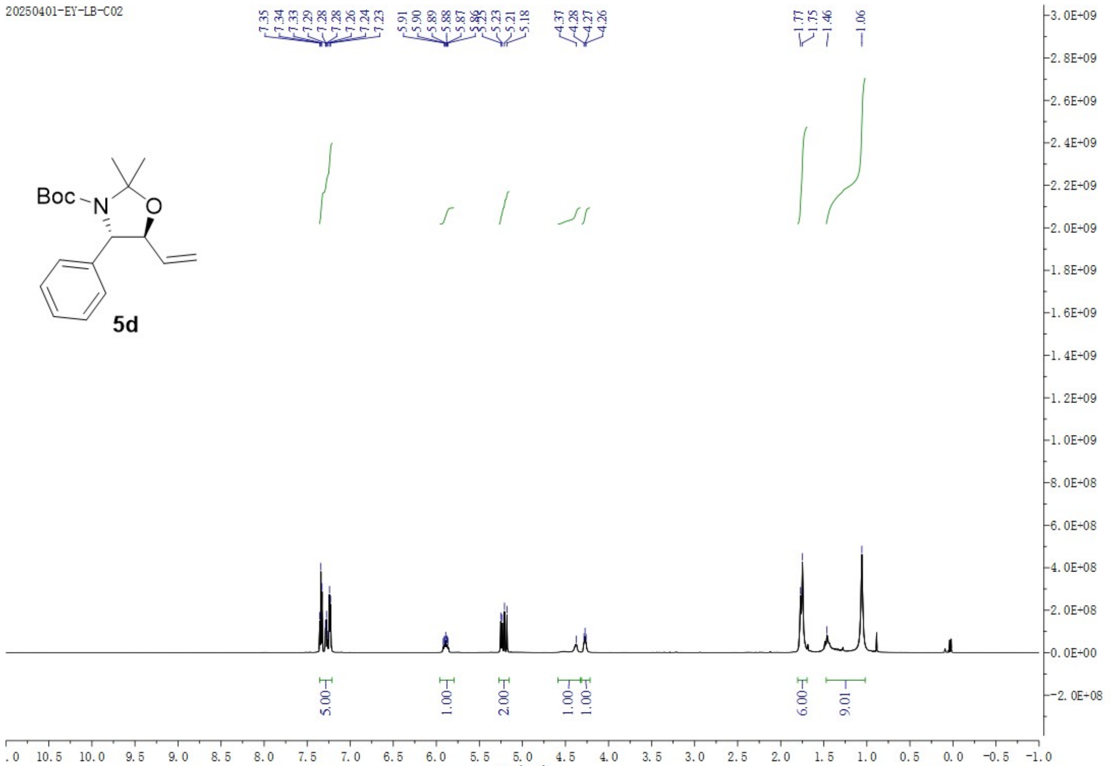

Figure S17. ^{1}H NMR of **5d** ($CDCl_3$, 600 MHz) (*exists as rotamers*)

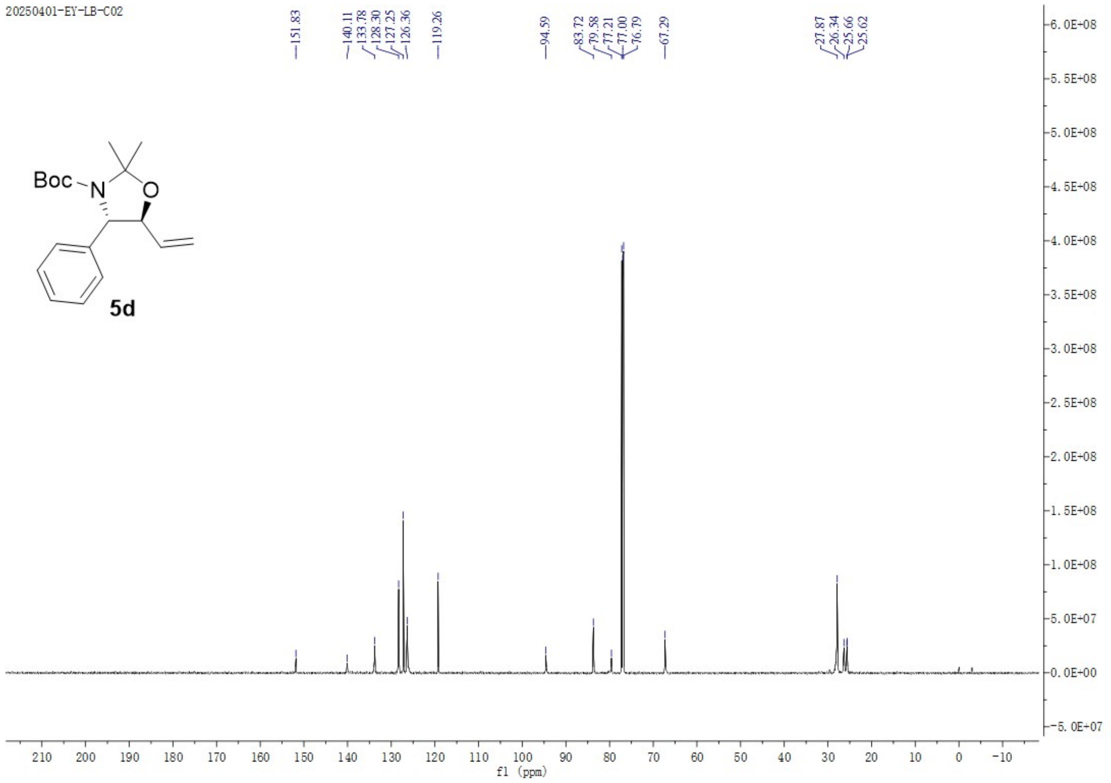

Figure S18. ^{13}C NMR of **5d** ($CDCl_3$, 151 MHz) (*exists as rotamers*)

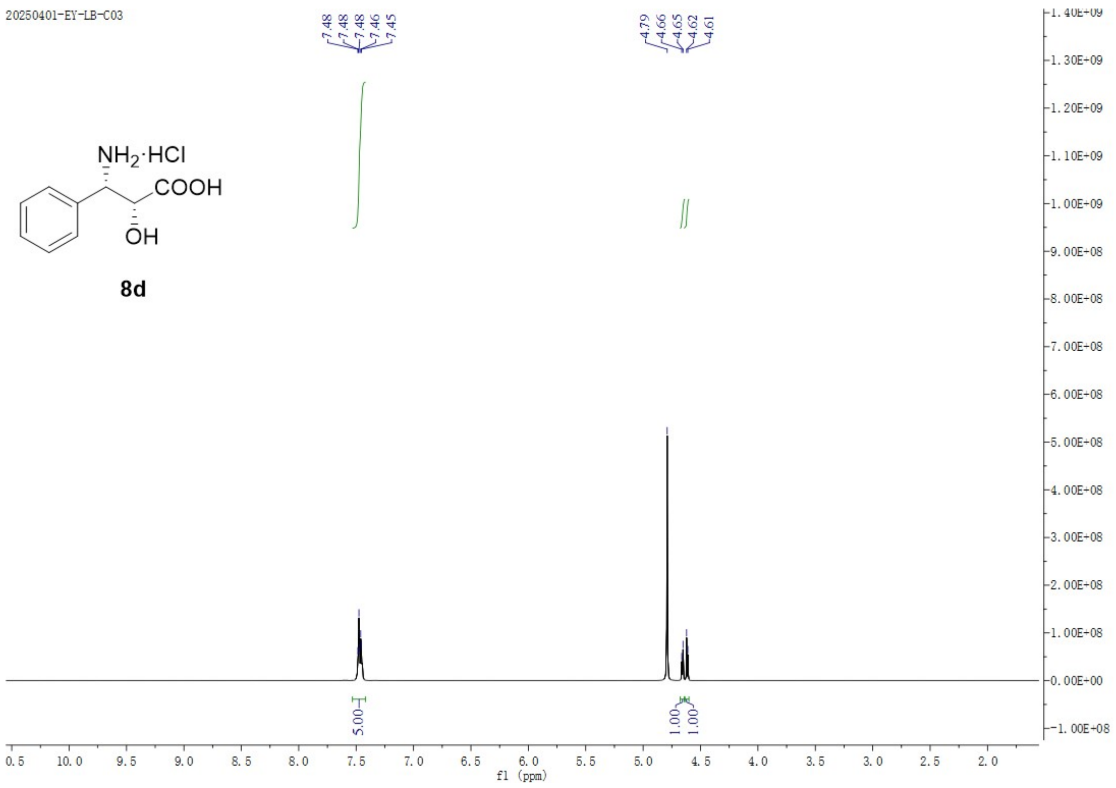

Figure S19. ^{1}H NMR of **8d** (D_2O, 600 MHz)

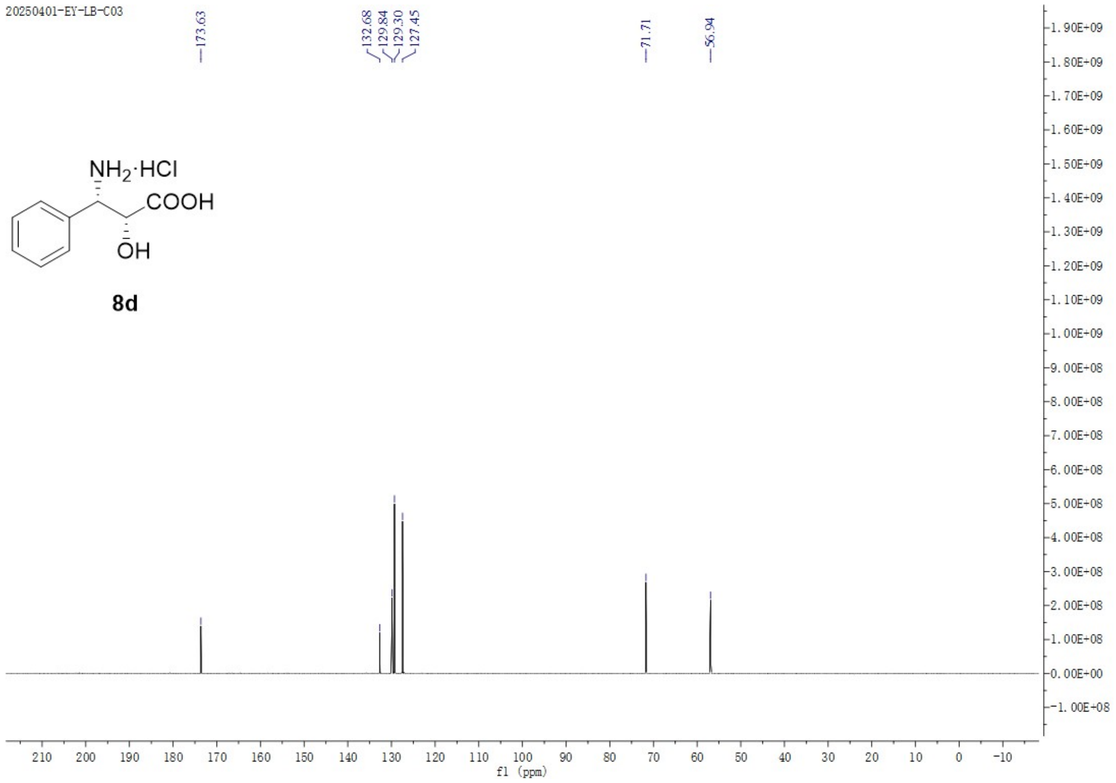

Figure S20. ^{13}C NMR of **8d** (D_2O, 151 MHz)

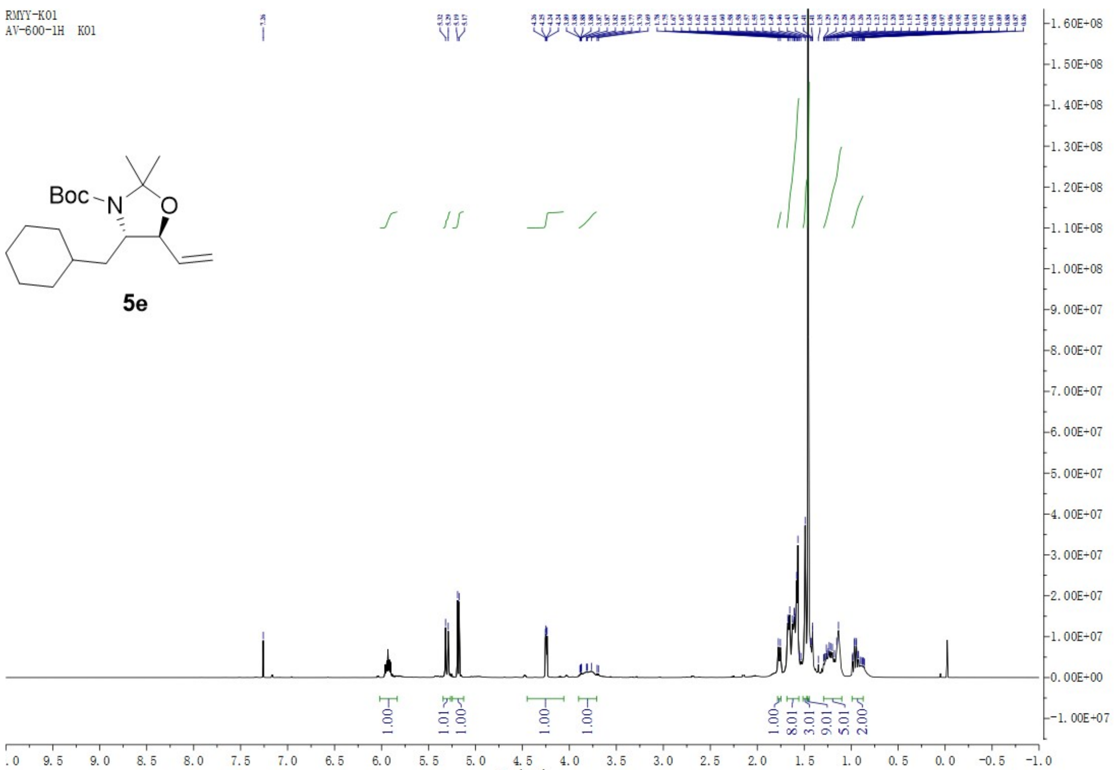

Figure S21. ^{1}H NMR of **5e** ($CDCl_3$, 600 MHz) (*exists as rotamers*)

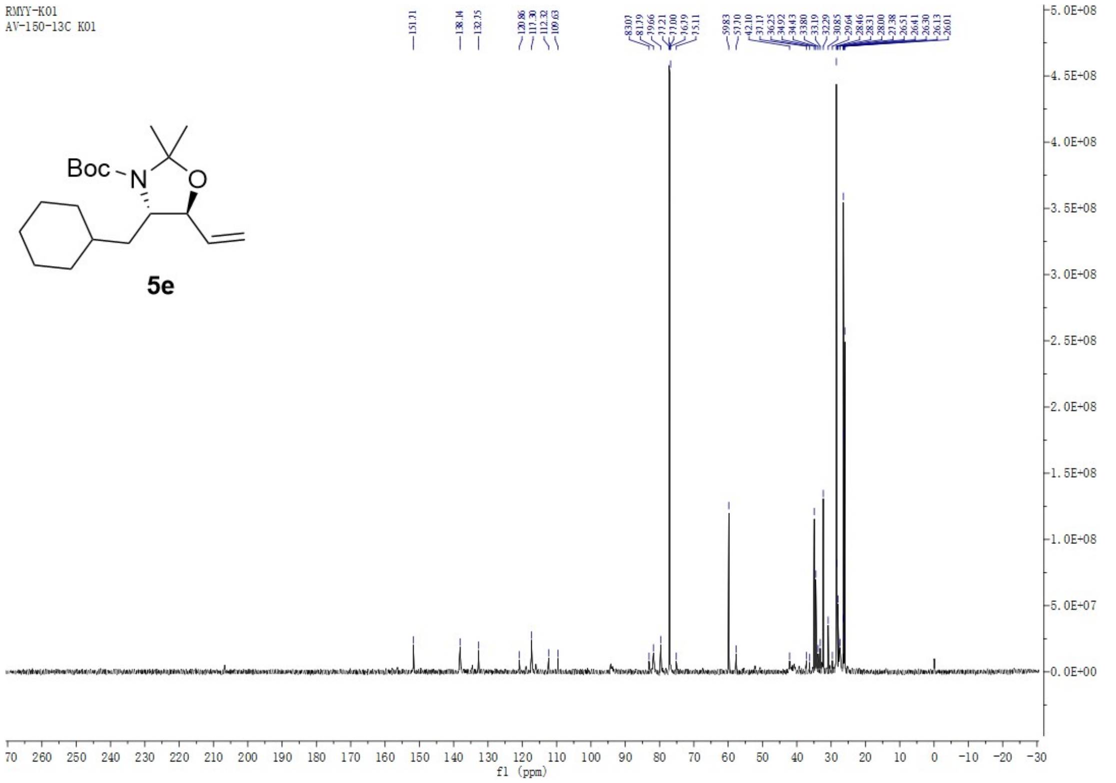

Figure S22. ^{13}C NMR of **5e** ($CDCl_3$, 151 MHz) (*exists as rotamers*)

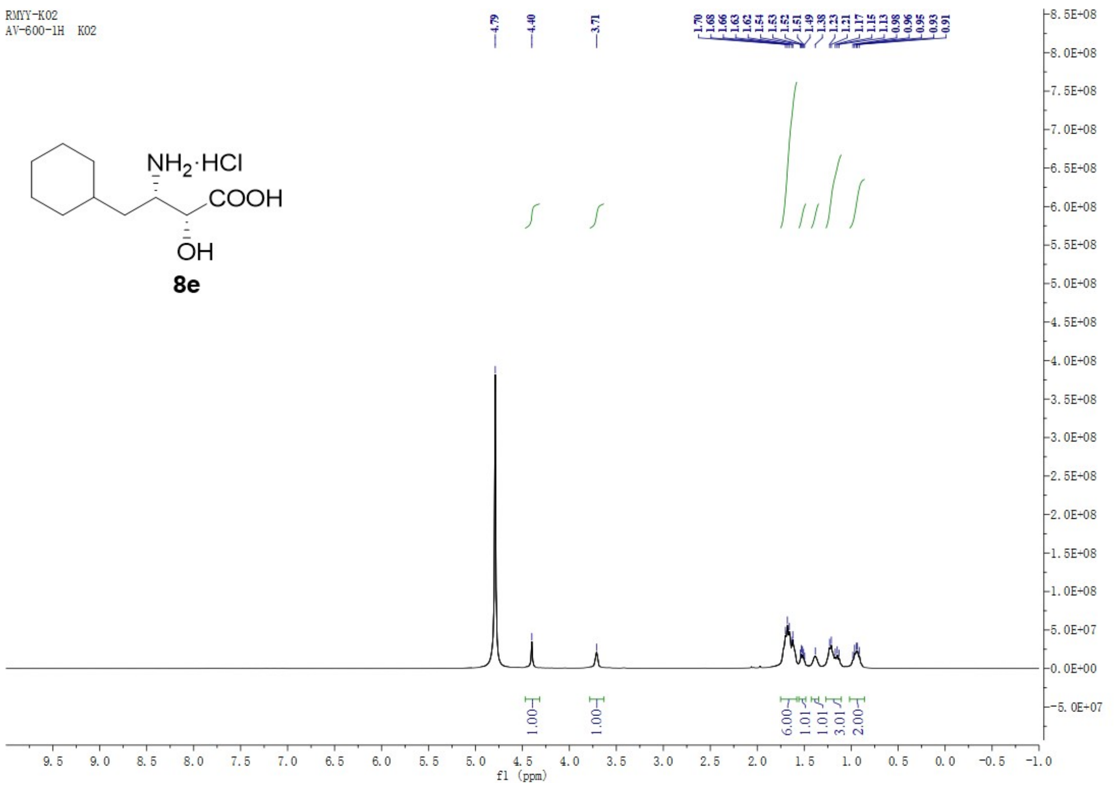

Figure S23. ^{1}H NMR of **8e** (D_2O, 600 MHz)

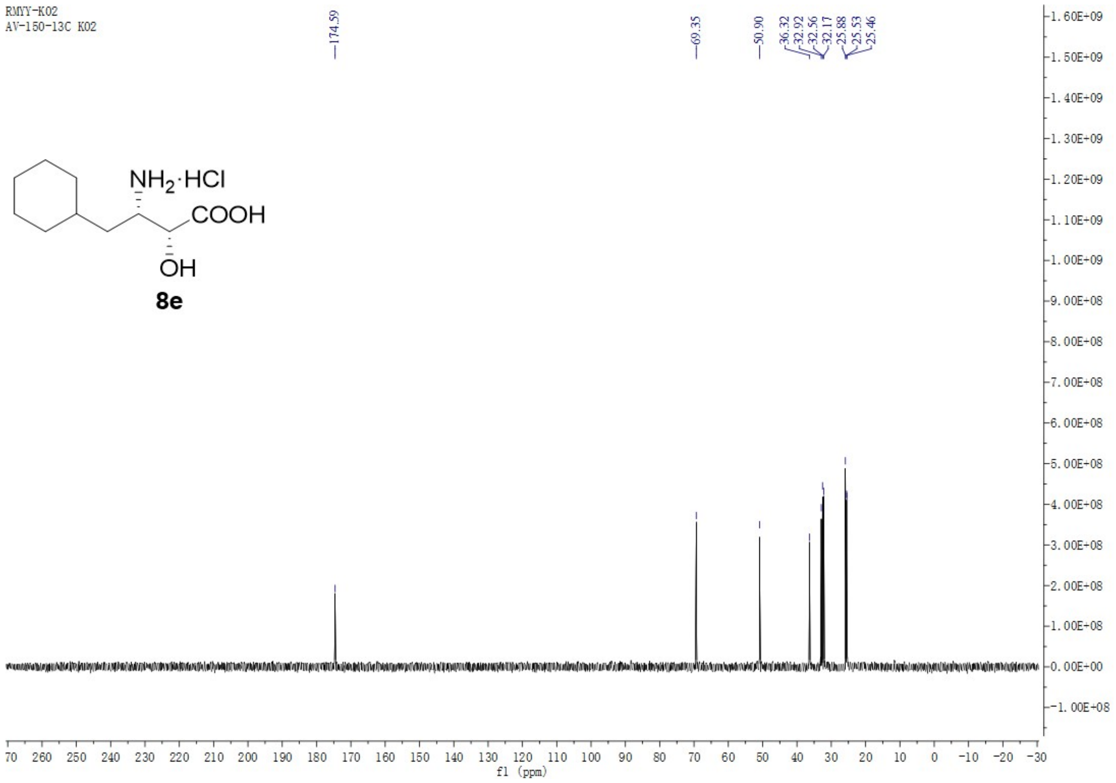

Figure S24. ^{13}C NMR of **8e** (D_2O, 151 MHz)

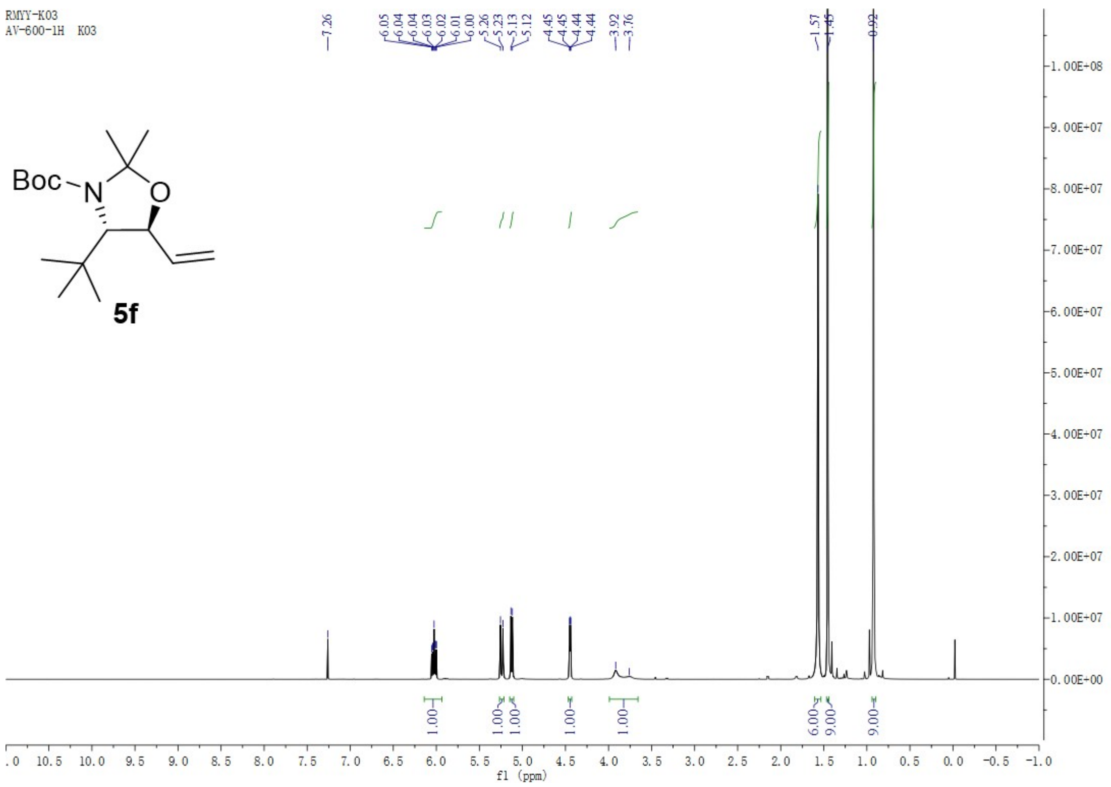

Figure S25. ^{1}H NMR of **5f** ($CDCl_3$, 600 MHz) (*exists as rotamers*)

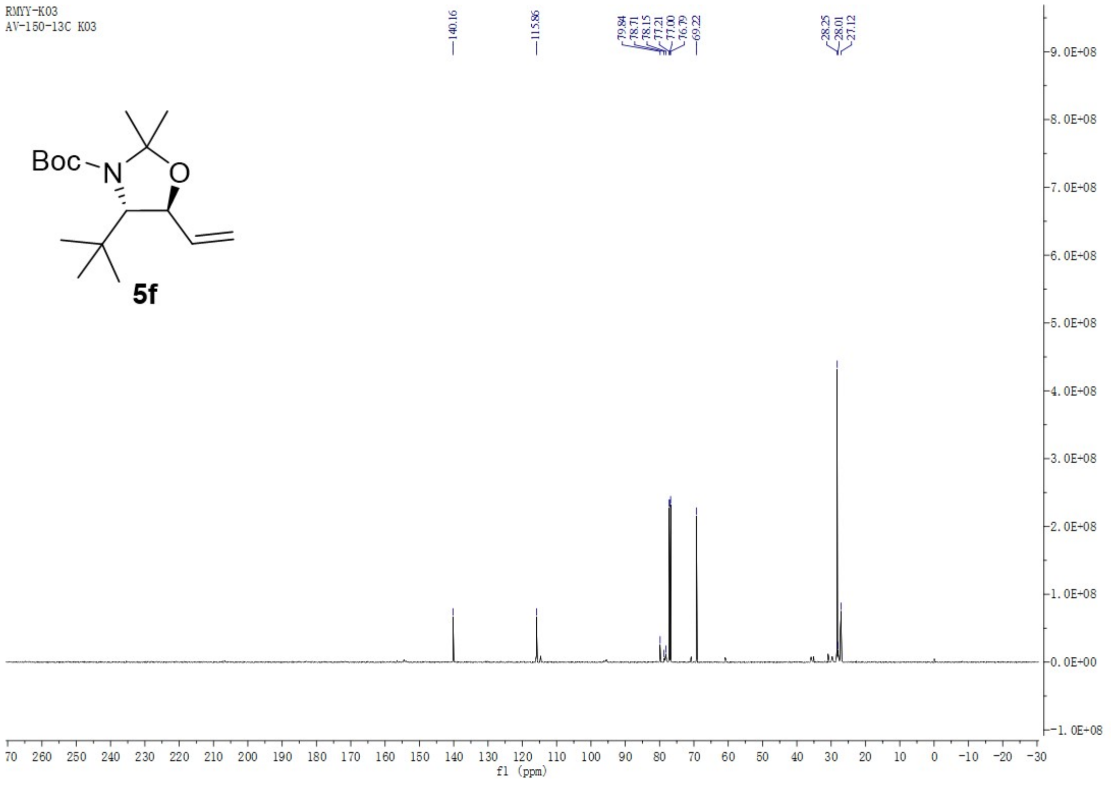

Figure S26. ^{13}C NMR of **5f** ($CDCl_3$, 151 MHz) (*exists as rotamers*)

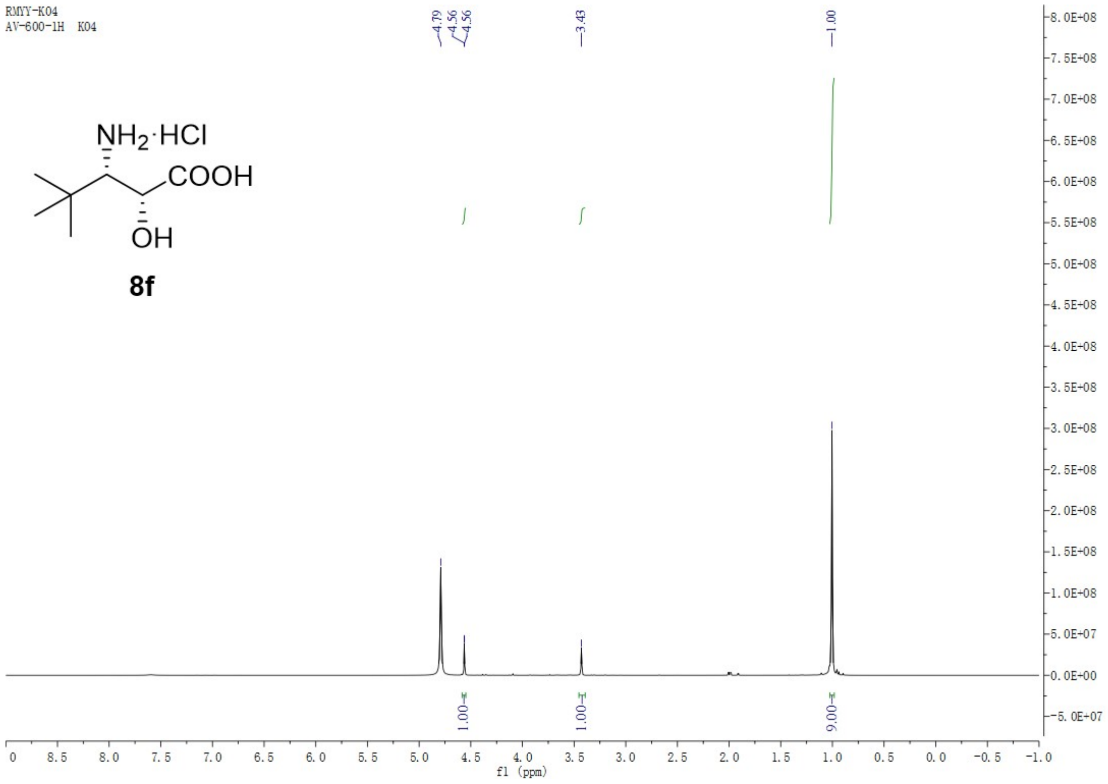

Figure S27. ^{1}H NMR of **8f** (D_2O, 600 MHz)

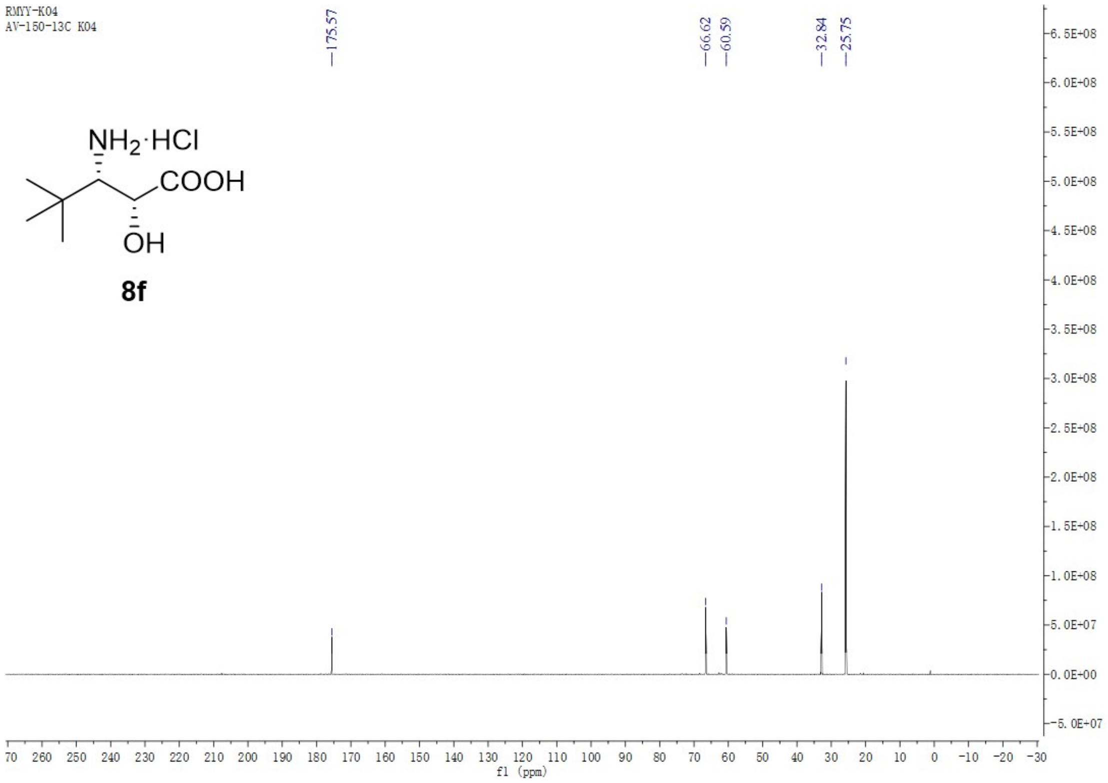

Figure S28. ^{13}C NMR of **8f** (D_2O, 151 MHz)

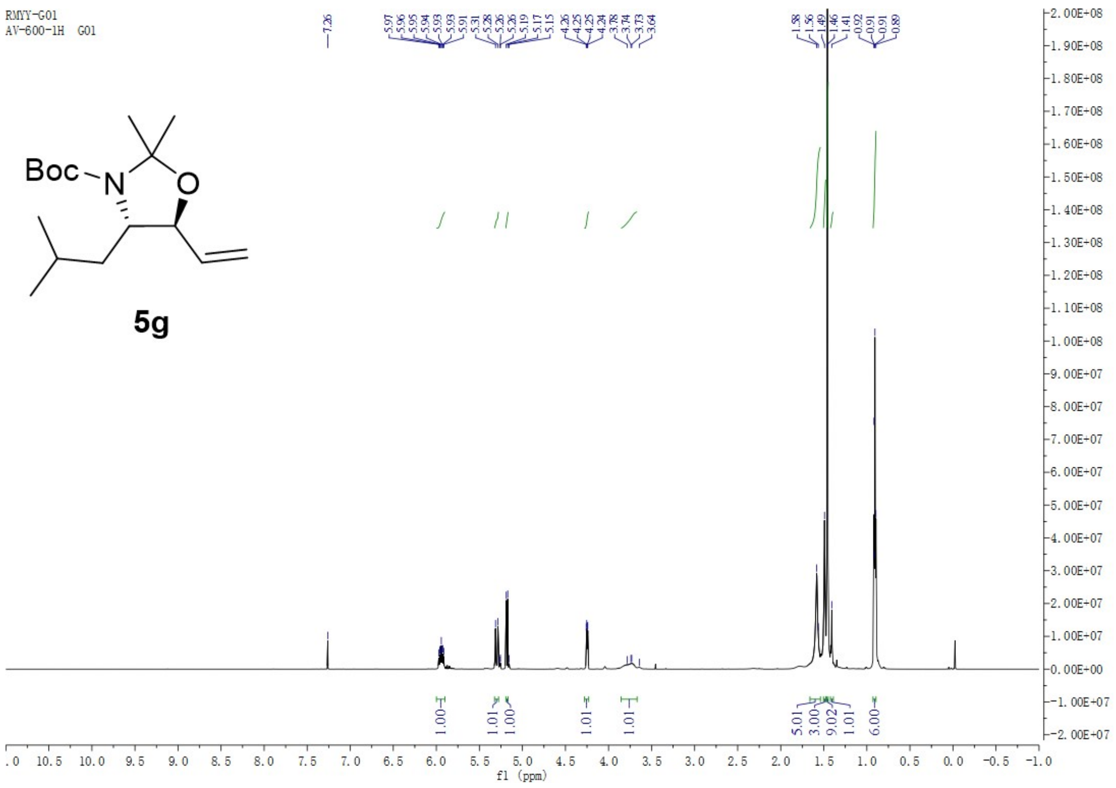

Figure S29. ^{1}H NMR of **5g** ($CDCl_3$, 600 MHz) (*exists as rotamers*)

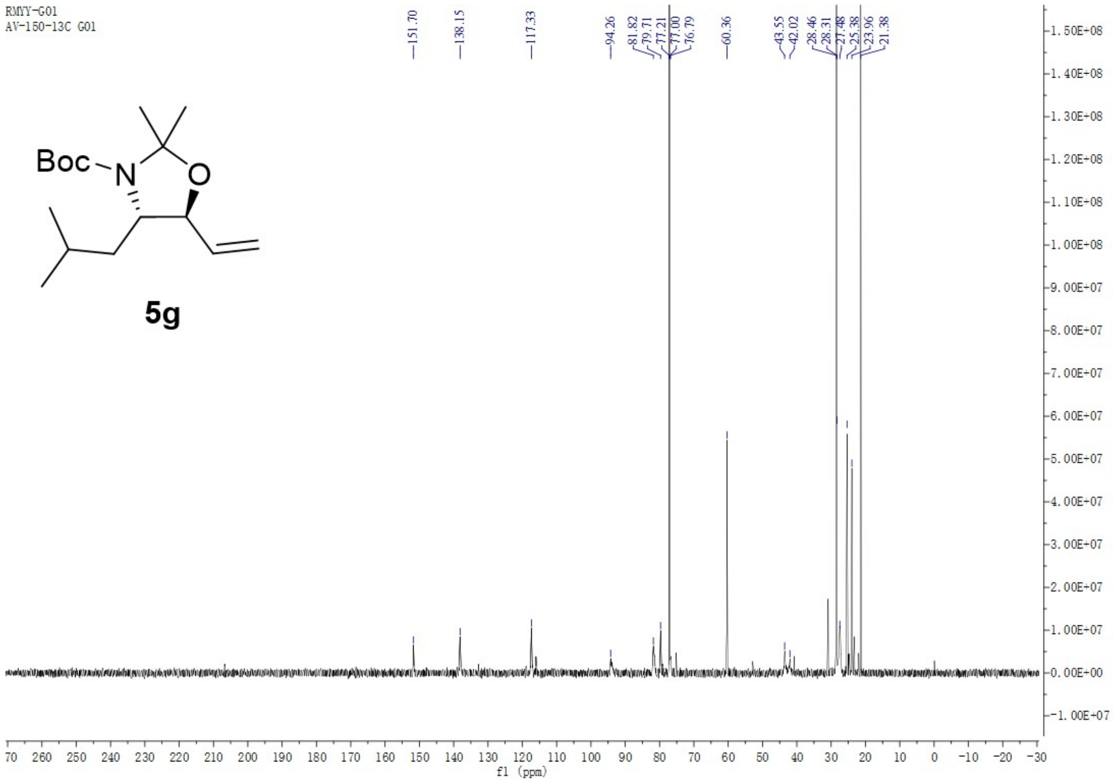

Figure S30. ^{13}C NMR of **5g** ($CDCl_3$, 151 MHz) (*exists as rotamers*)

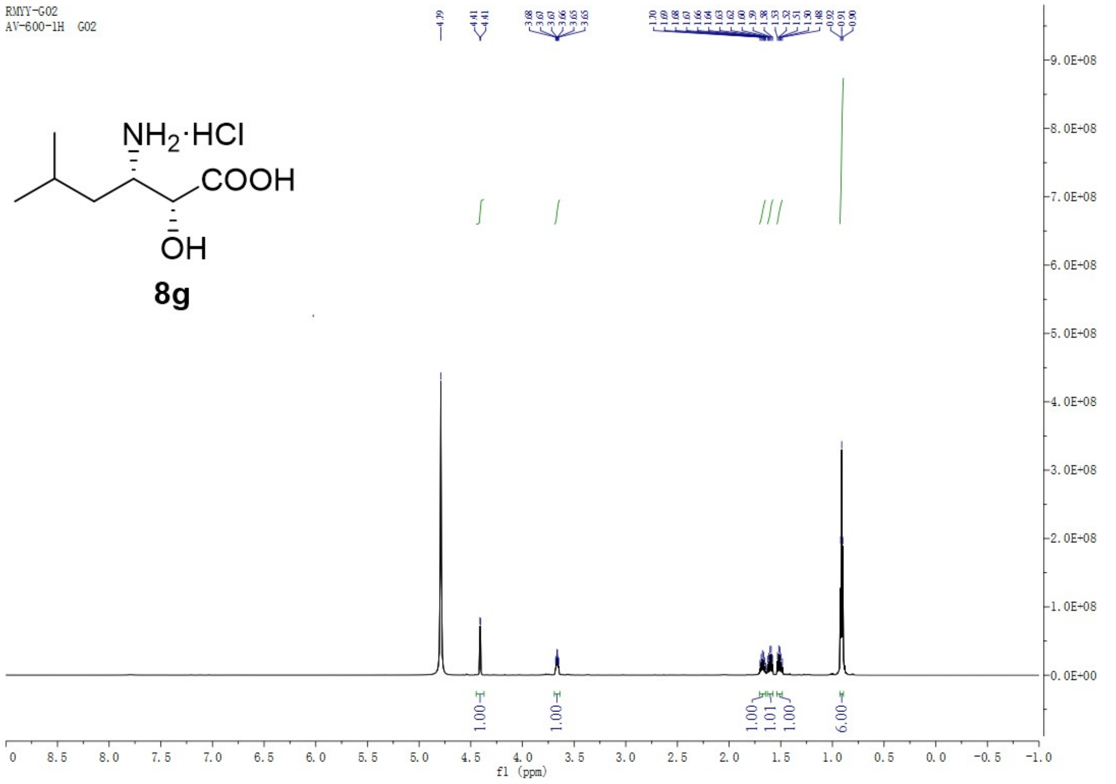

Figure S31. ^{1}H NMR of **8g** (D_2O, 600 MHz)

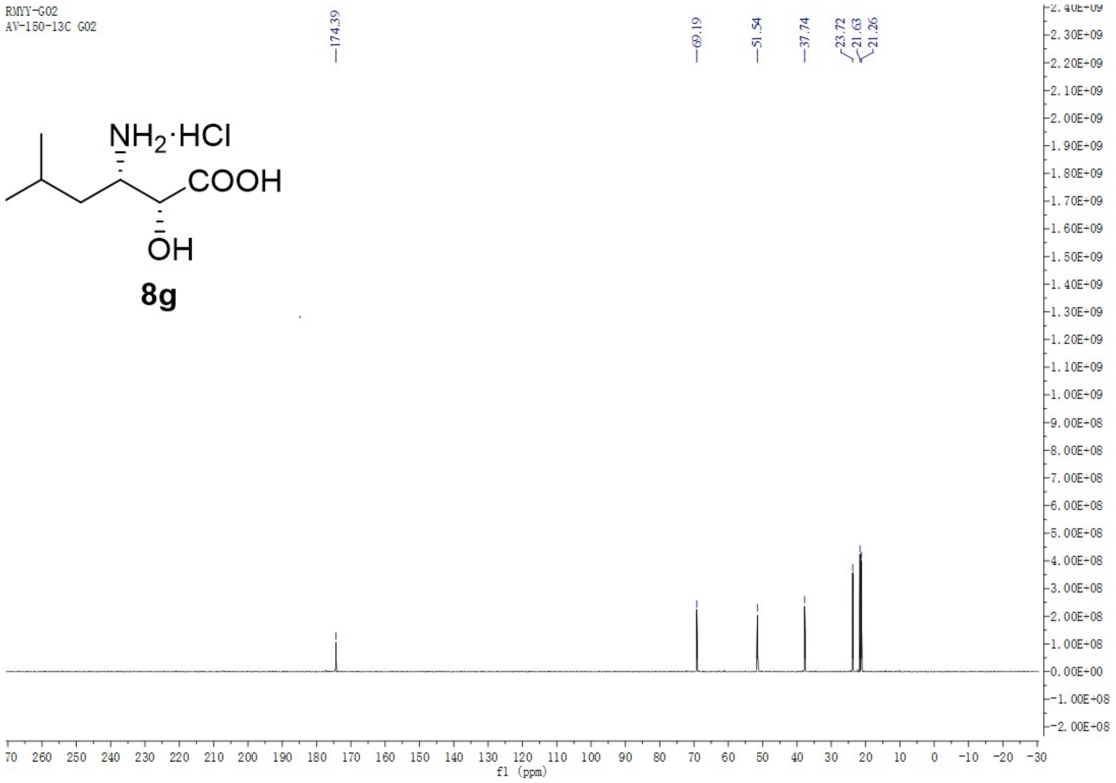

Figure S32. ^{13}C NMR of **8g** (D_2O, 151 MHz)

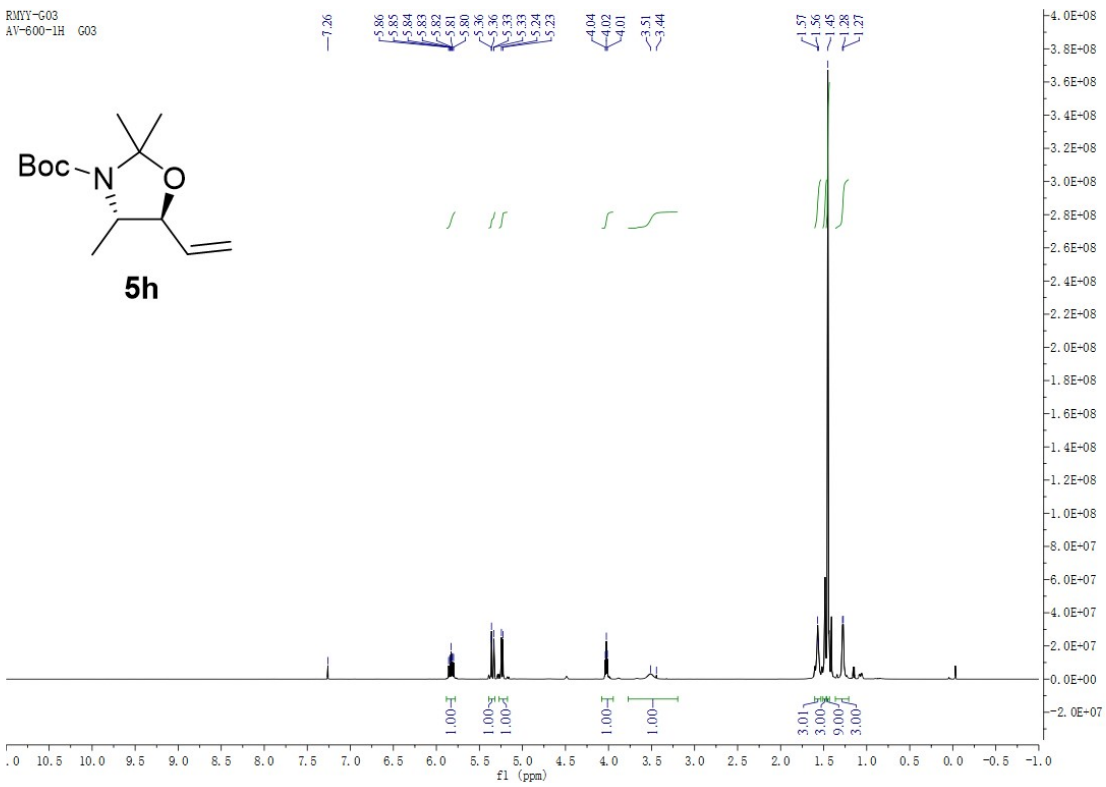

Figure S33. ^{1}H NMR of **5h** ($CDCl_3$, 600 MHz) (*exists as rotamers*)

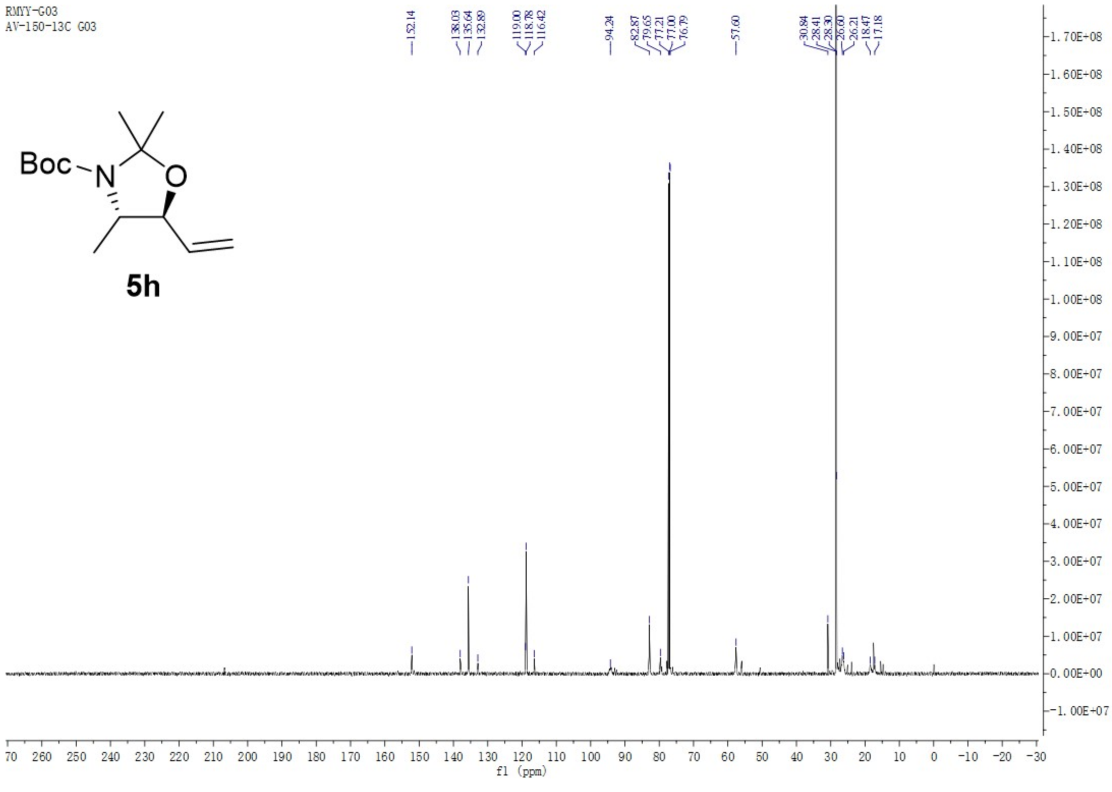

Figure S34. ^{13}C NMR of **5h** ($CDCl_3$, 151 MHz) (*exists as rotamers*)

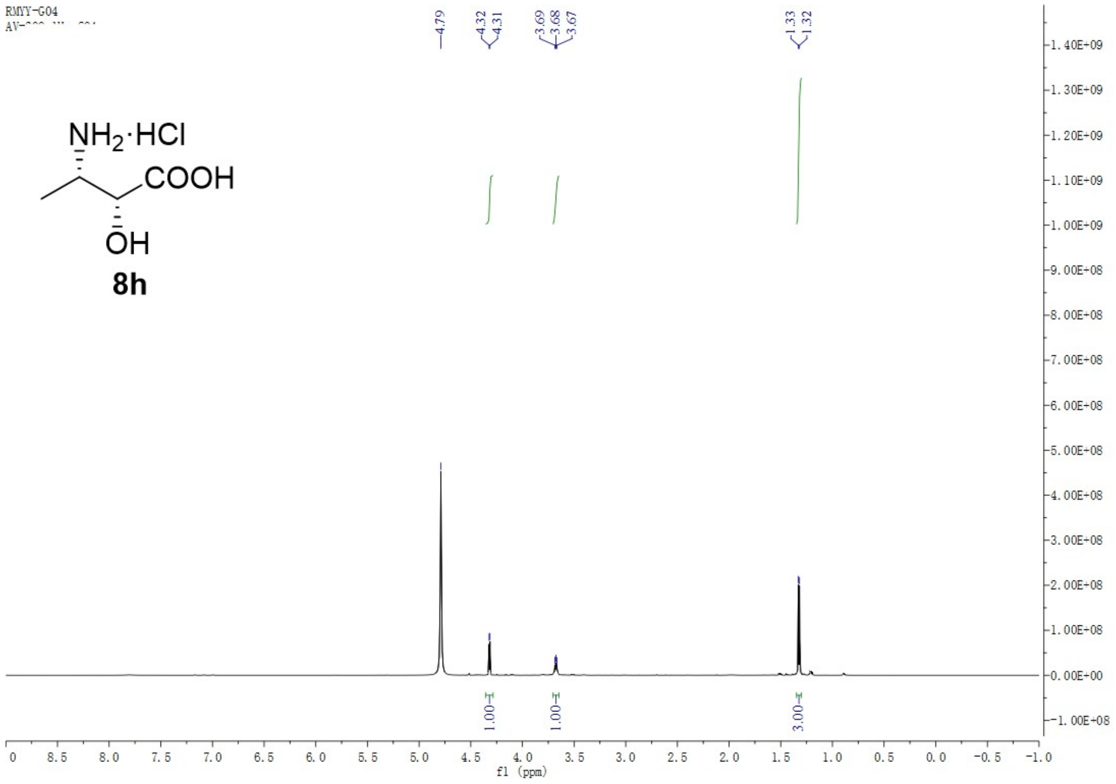

Figure S35. ^{1}H NMR of **8h** (D_2O, 600 MHz)

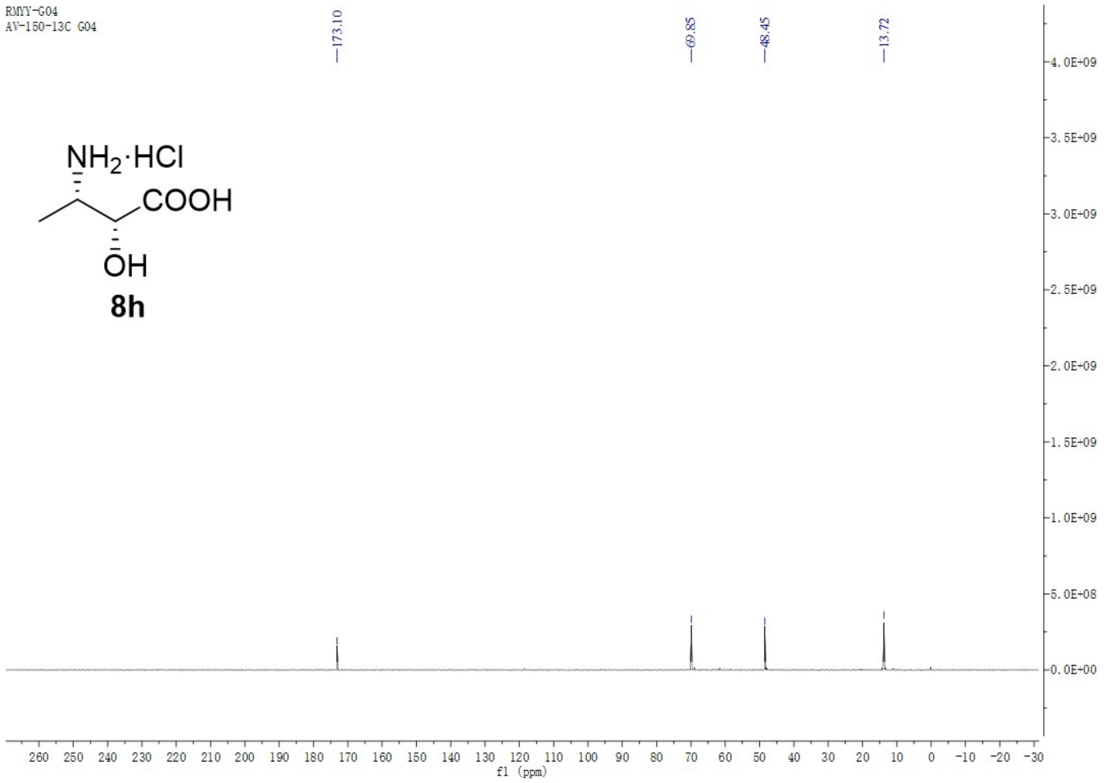

Figure S36. ^{13}C NMR of **8h** (D_2O, 151 MHz)

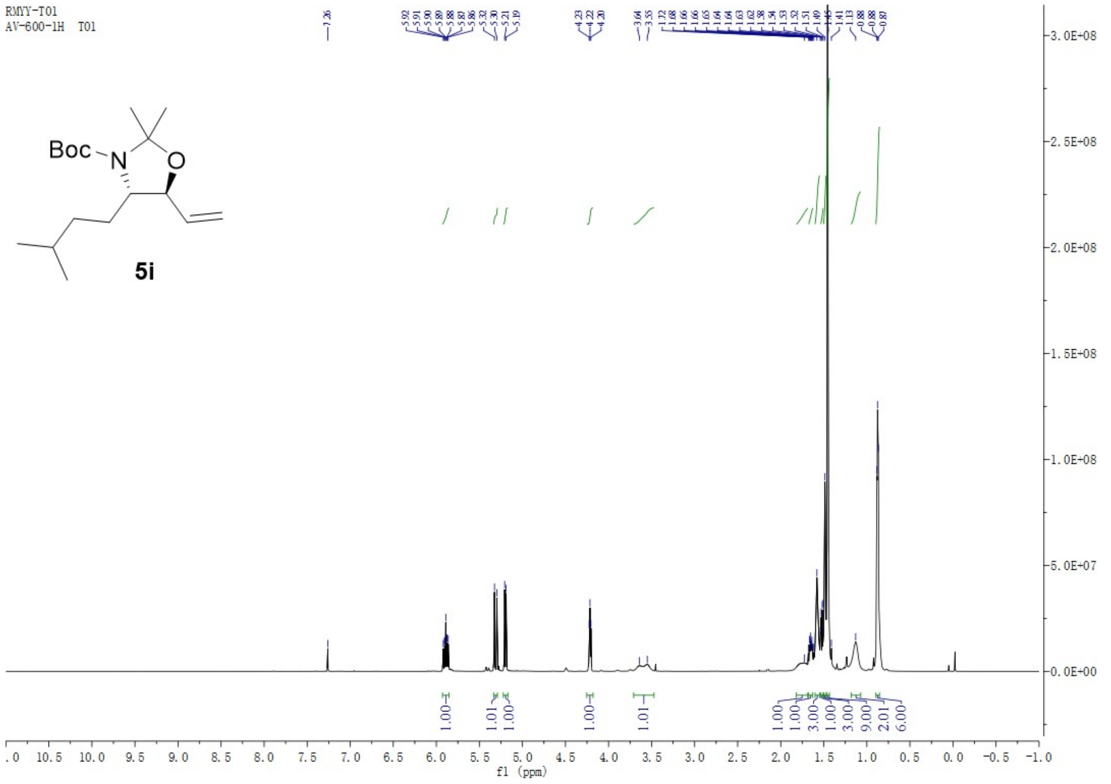

Figure S37. ^{1}H NMR of **5i** ($CDCl_3$, 600 MHz) (*exists as rotamers*)

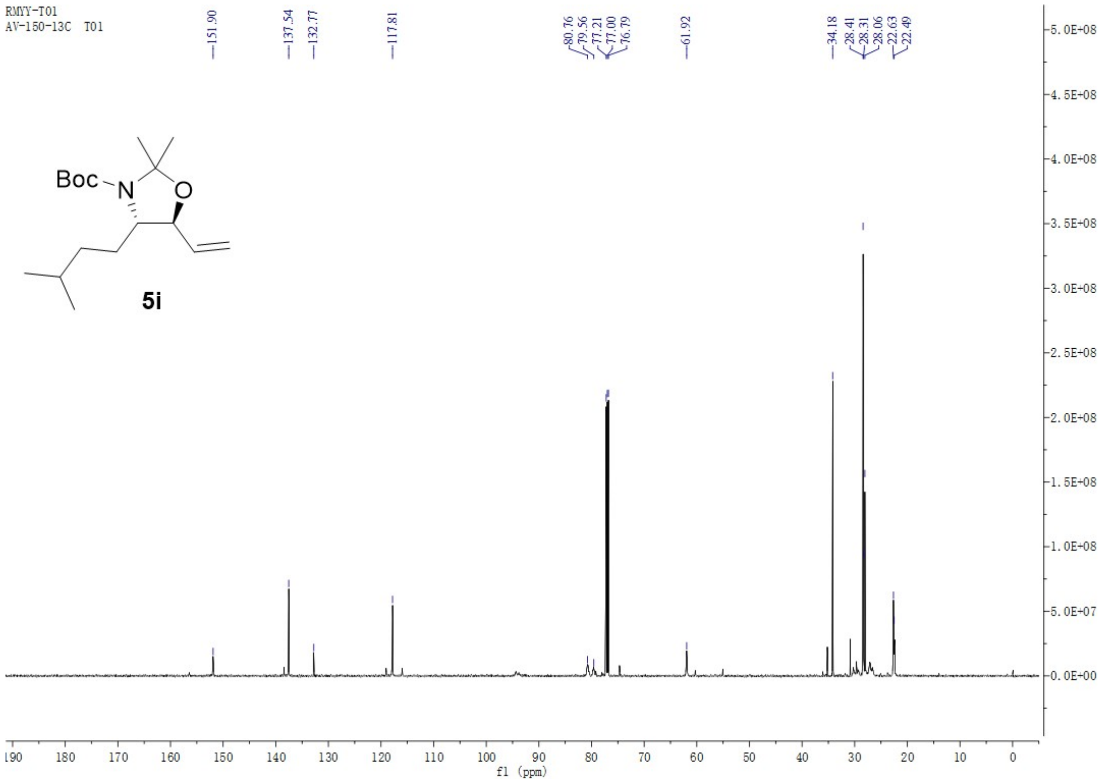

Figure S38. ^{13}C NMR of **5i** ($CDCl_3$, 151 MHz) (*exists as rotamers*)

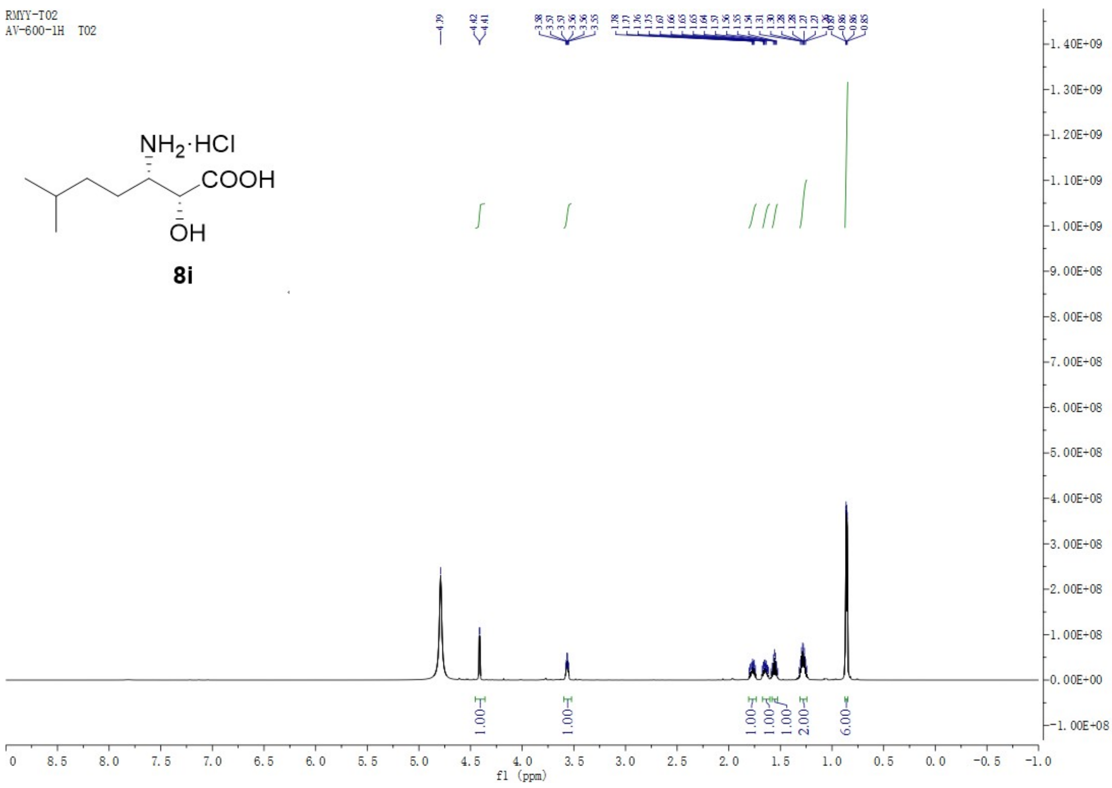

Figure S39. ^{1}H NMR of **8i** (D_2O, 600 MHz)

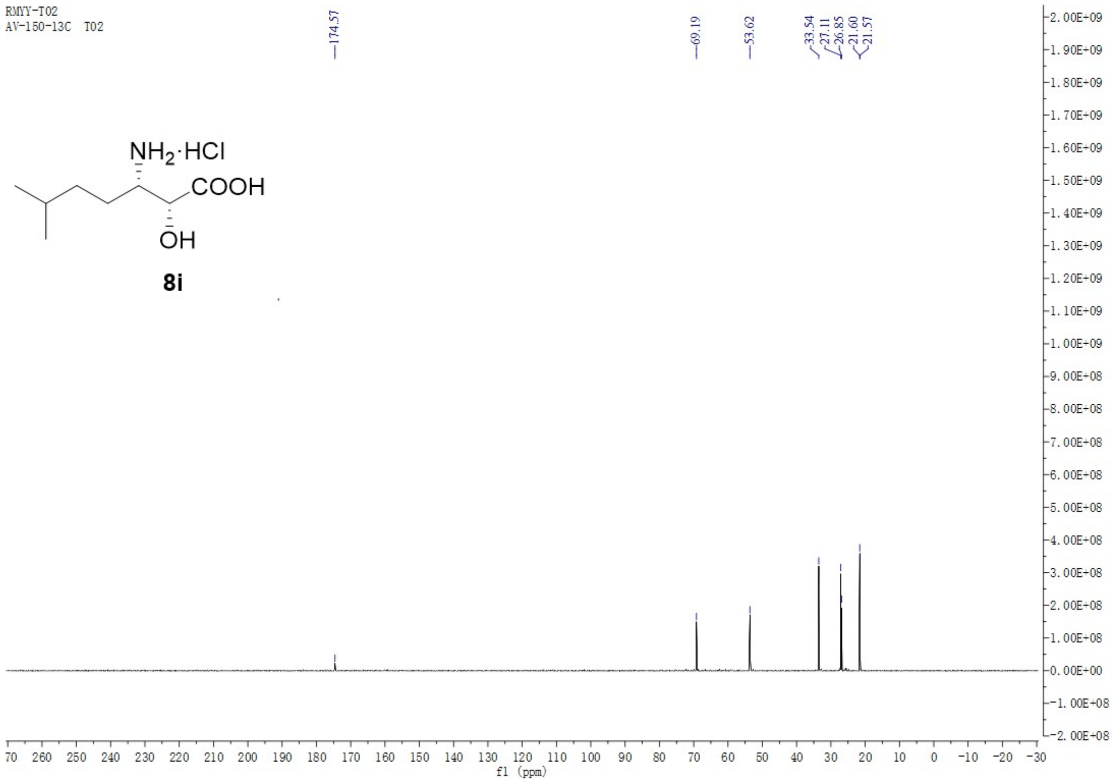

Figure S40. ^{13}C NMR of **8i** (D_2O, 151 MHz)

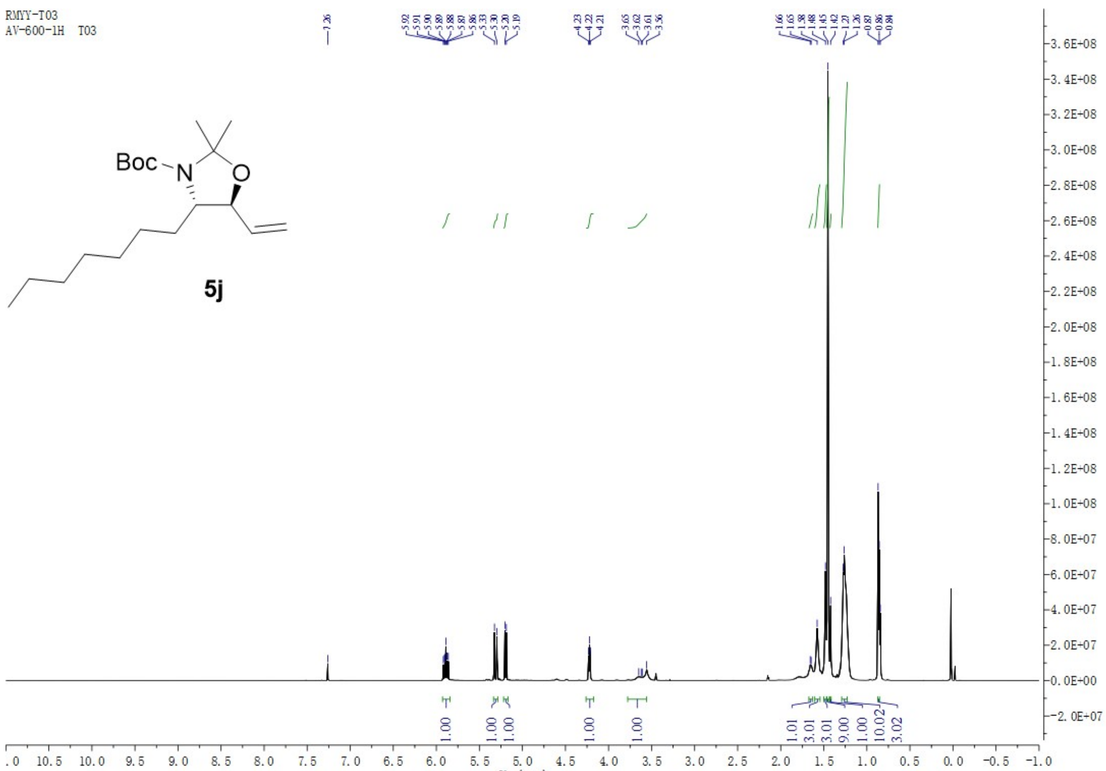

Figure S41. ^{1}H NMR of **5j** ($CDCl_3$, 600 MHz) (*exists as rotamers*)

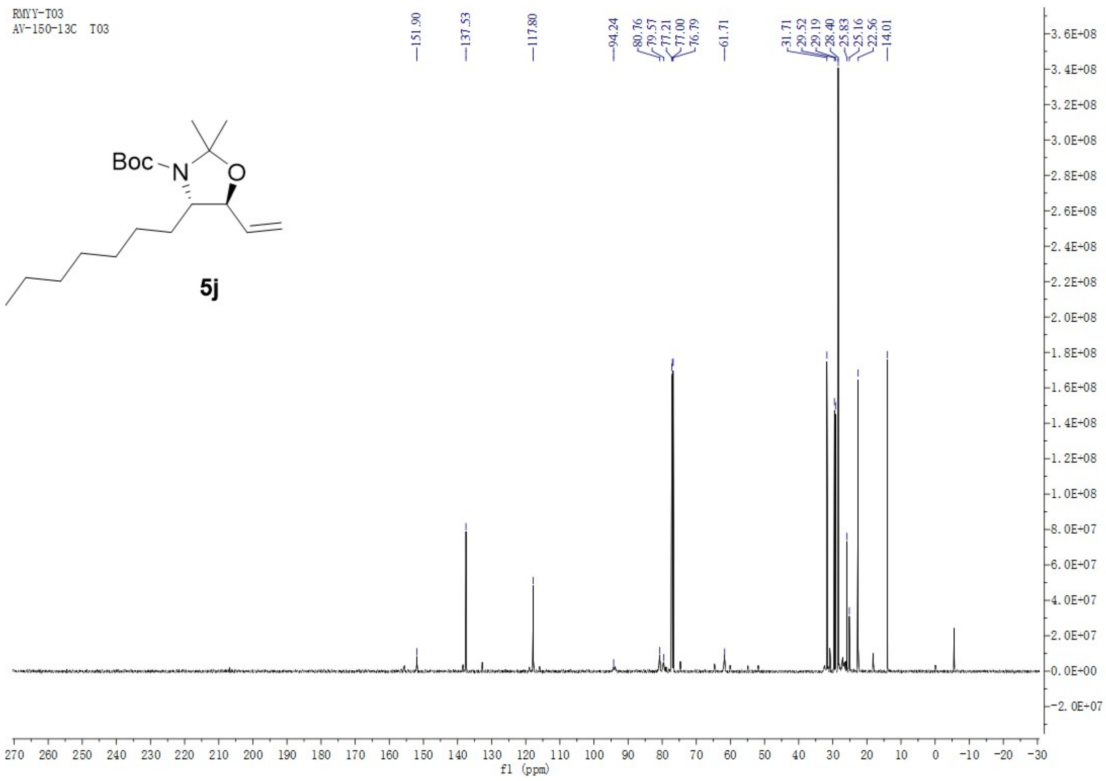

Figure S42. ^{13}C NMR of **5j** ($CDCl_3$, 151 MHz) (*exists as rotamers*)

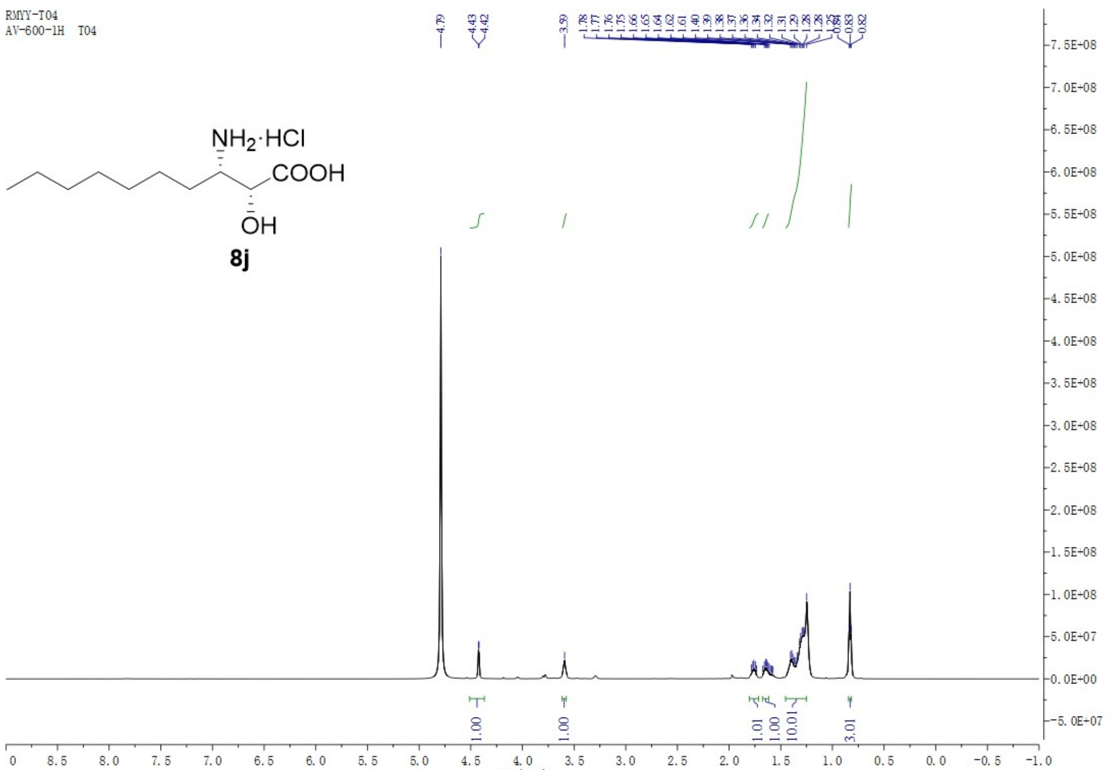

8j

Figure S43. ^{1}H NMR of **8j** (D_2O, 600 MHz)

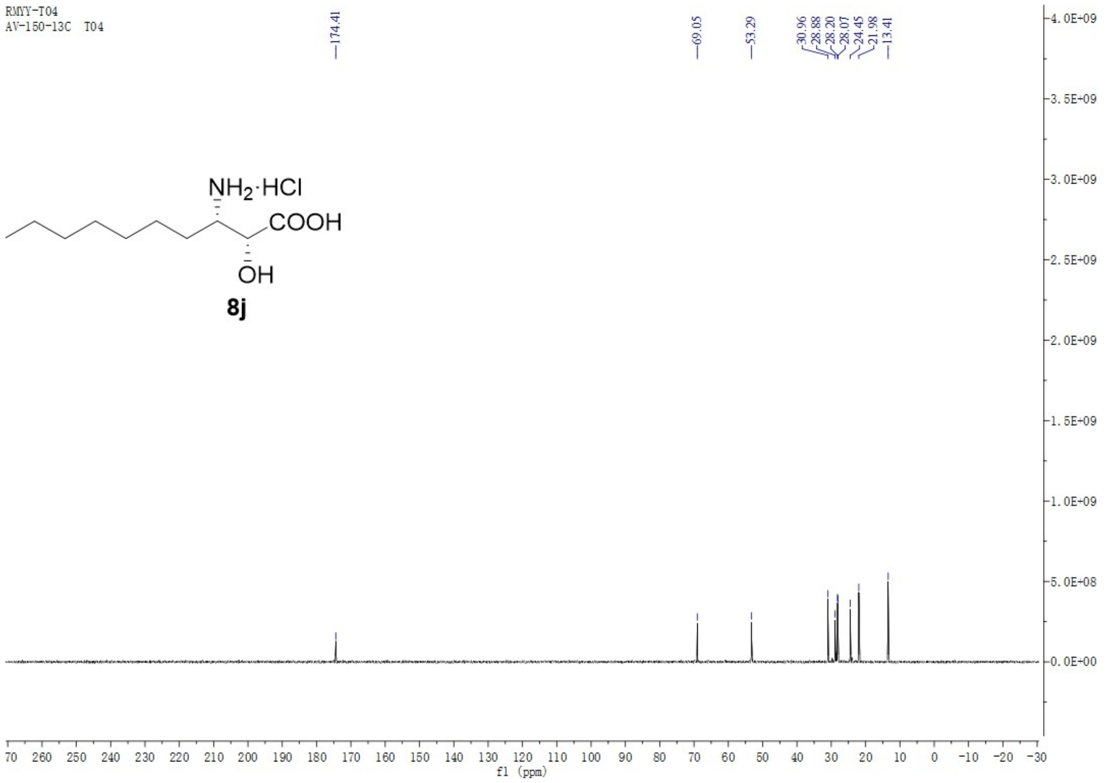

8j

Figure S44. ^{13}C NMR of **8j** (D_2O, 151 MHz)

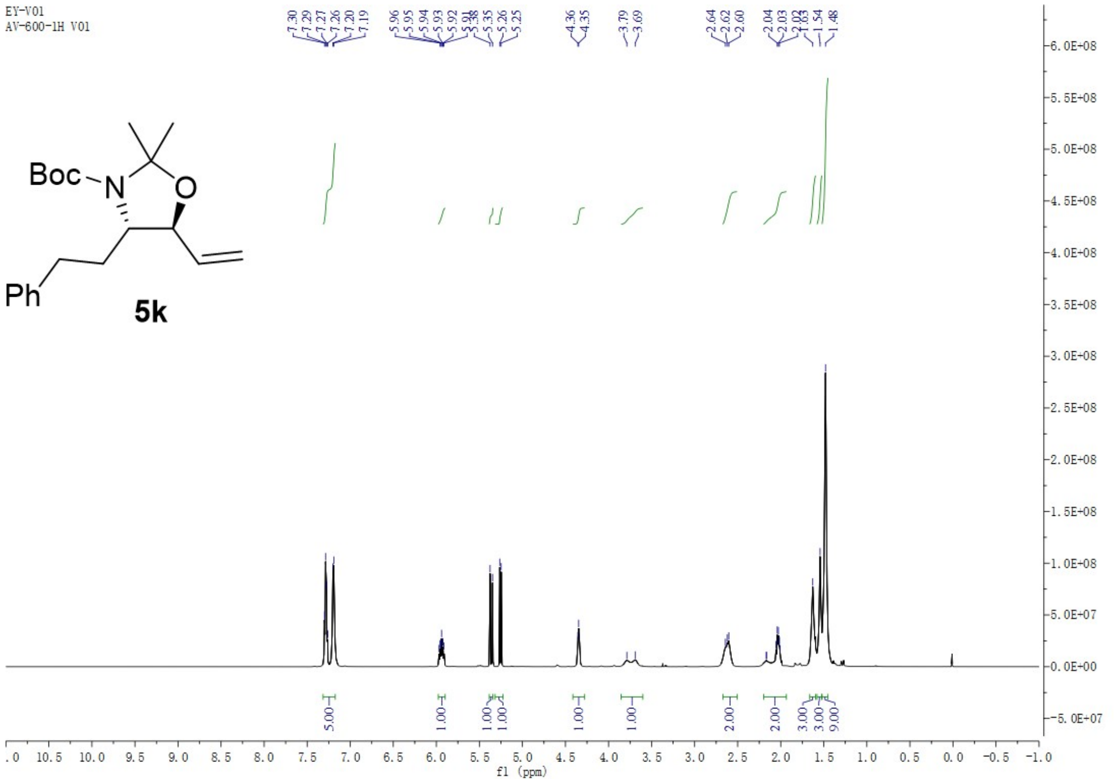

Figure S45. ^{1}H NMR of **5k** ($CDCl_3$, 600 MHz) (*exists as rotamers*)

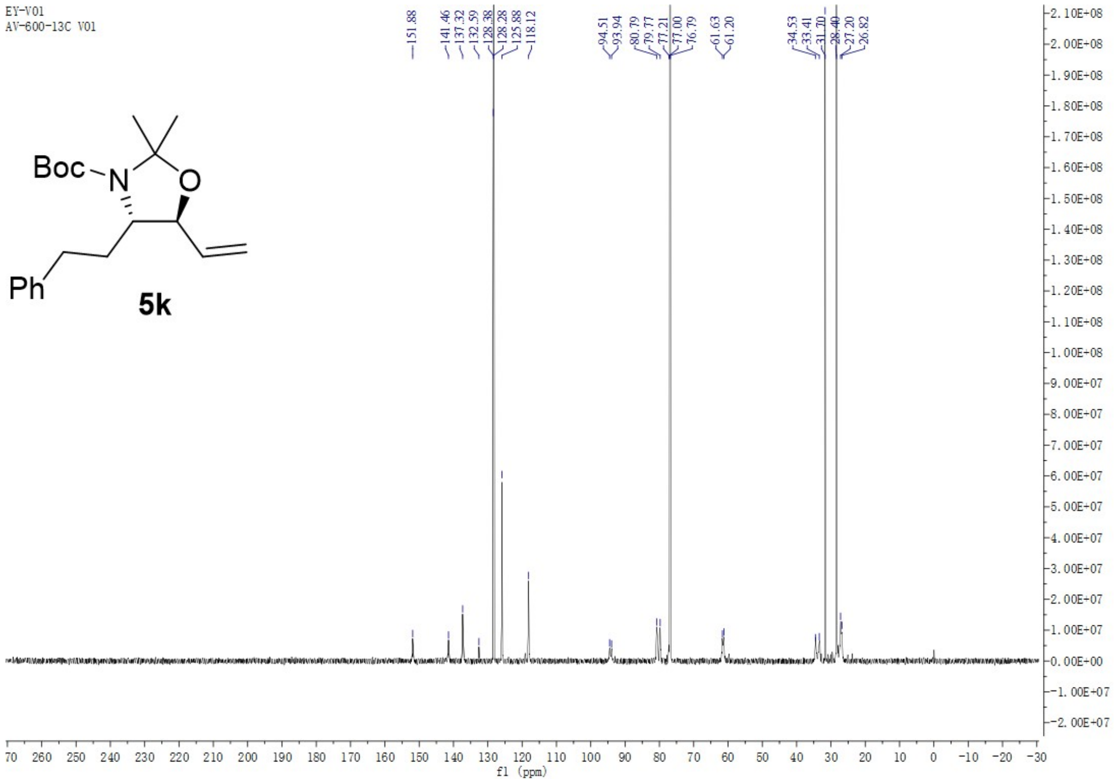

Figure S46. ^{13}C NMR of **5k** ($CDCl_3$, 151 MHz) (*exists as rotamers*)

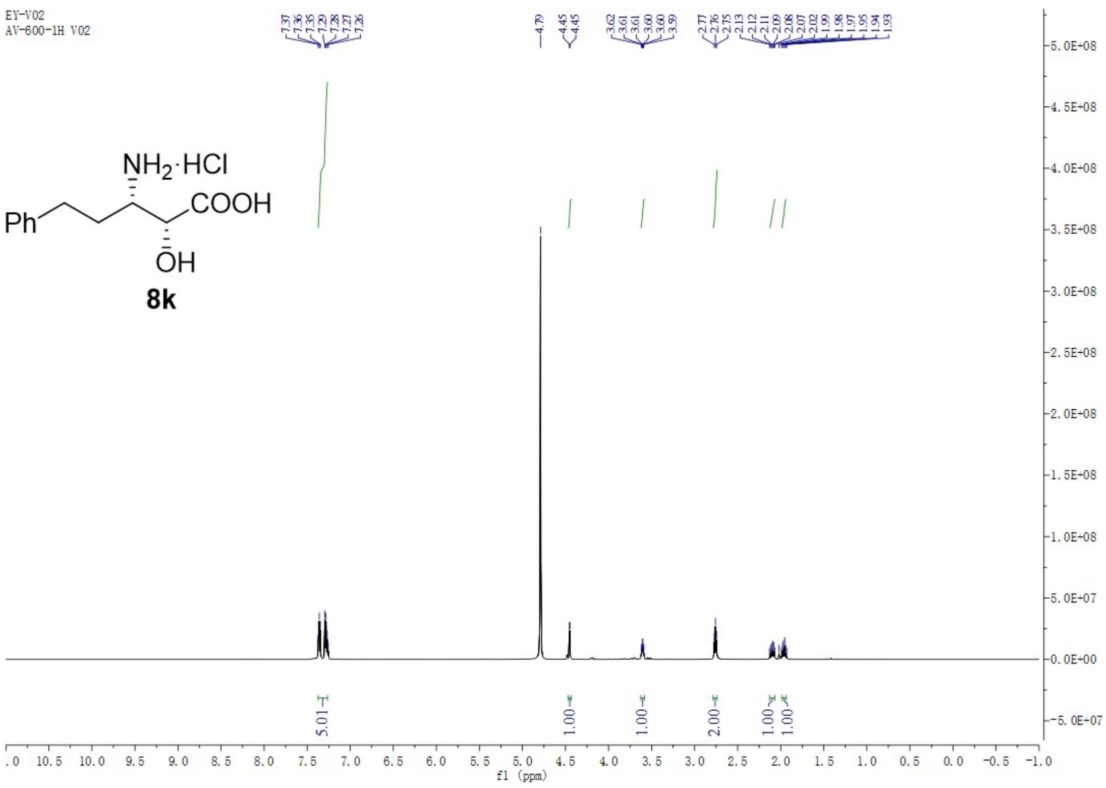

Figure S47. ^{1}H NMR of **8k** (D_2O, 600 MHz)

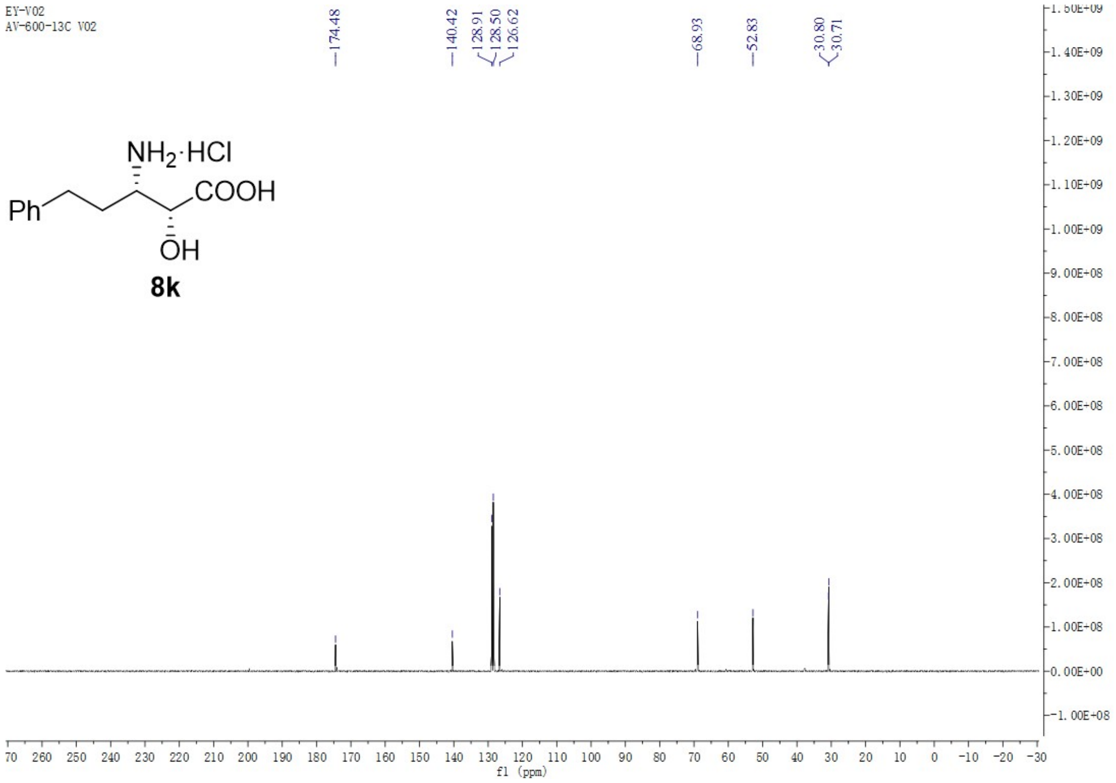

Figure S48. ^{13}C NMR of **8k** (D_2O, 151 MHz)

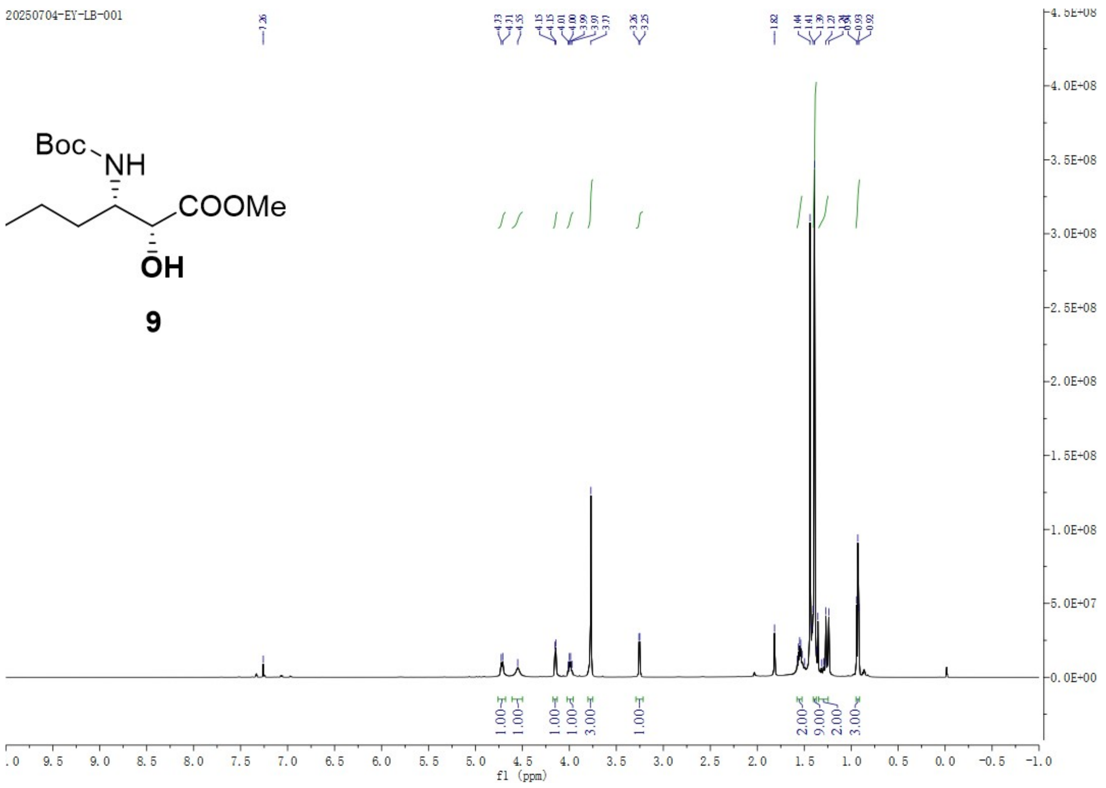

Figure S49. ^{1}H NMR of **9** ($CDCl_3$, 600 MHz)

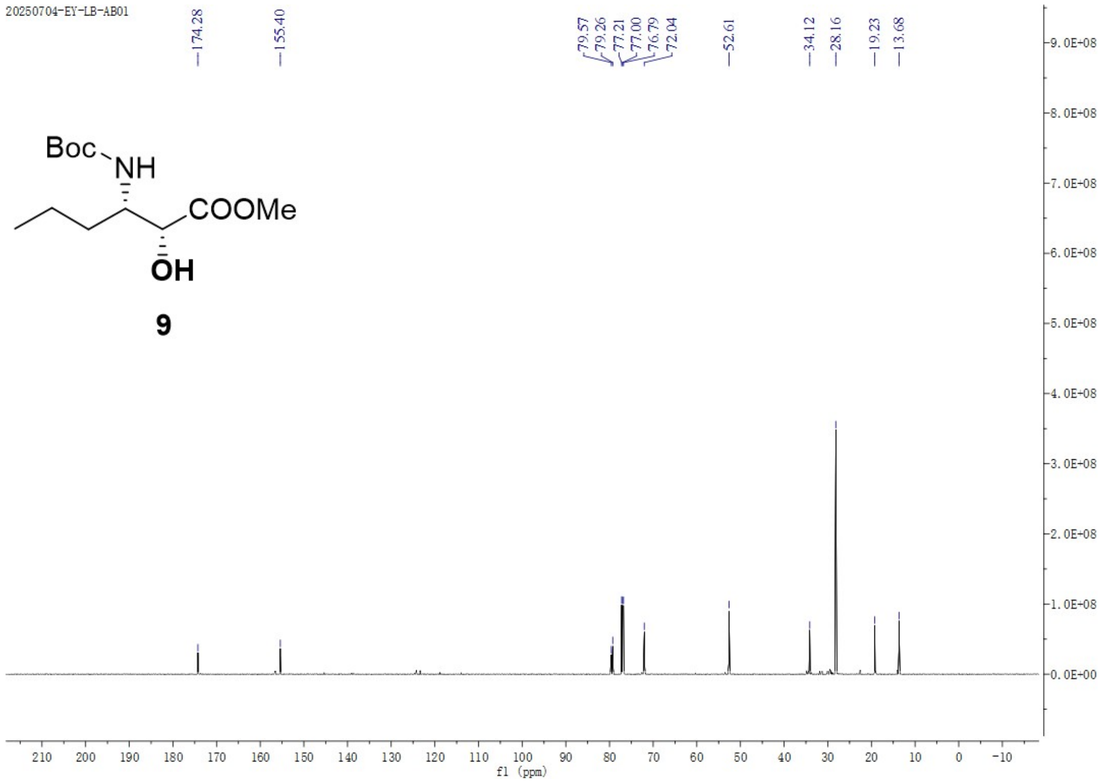

Figure S50. ^{13}C NMR of **9** ($CDCl_3$, 151 MHz)

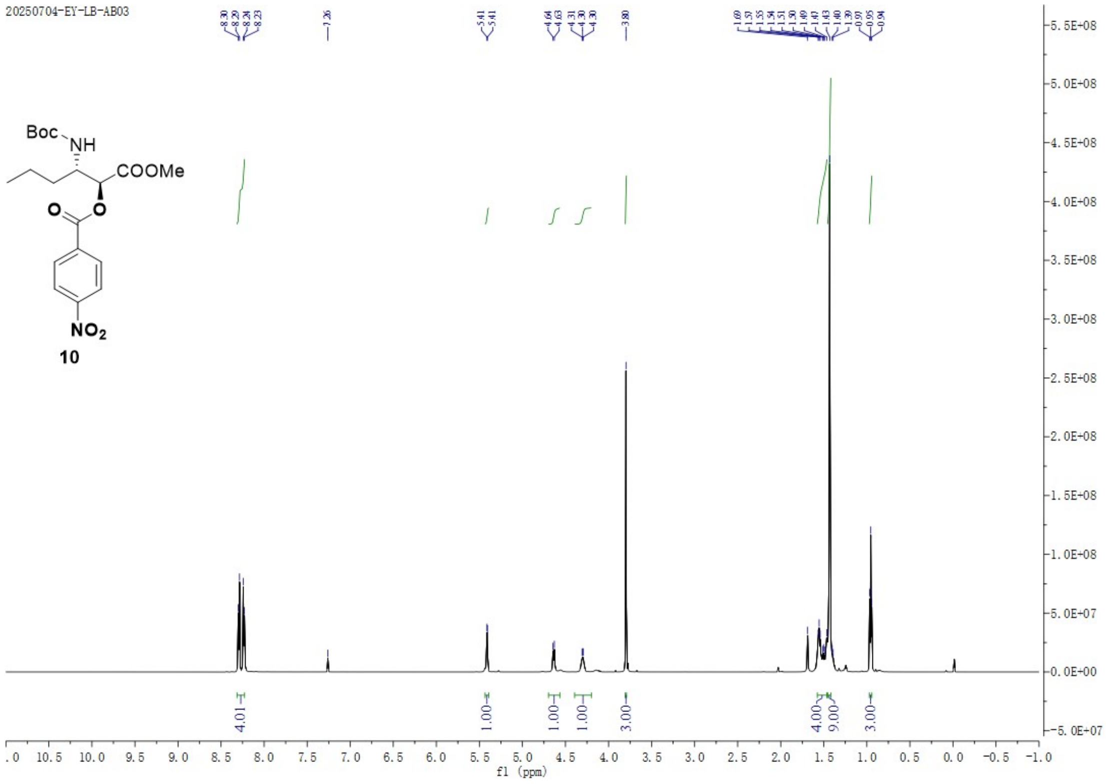

Figure S51. ^{1}H NMR of **10** ($CDCl_3$, 600 MHz)

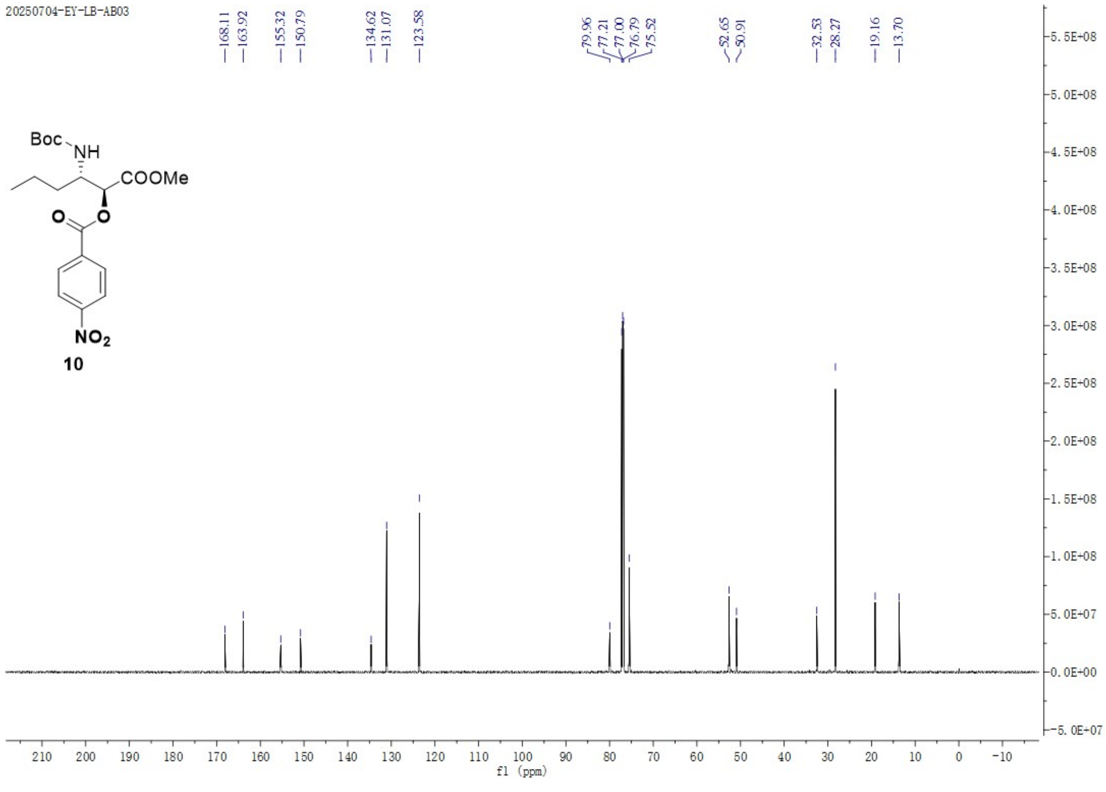

Figure S52. ^{13}C NMR of **10** ($CDCl_3$, 151 MHz)

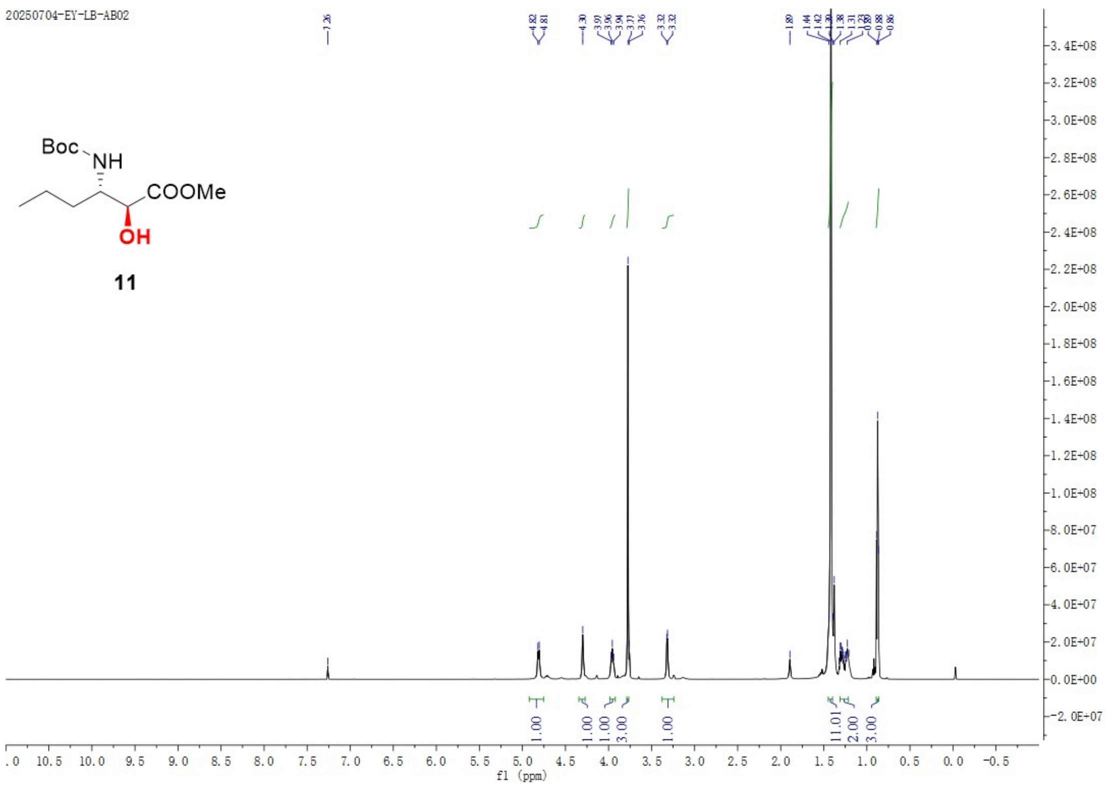

Figure S53. ^{1}H NMR of **11** ($CDCl_3$, 600 MHz)

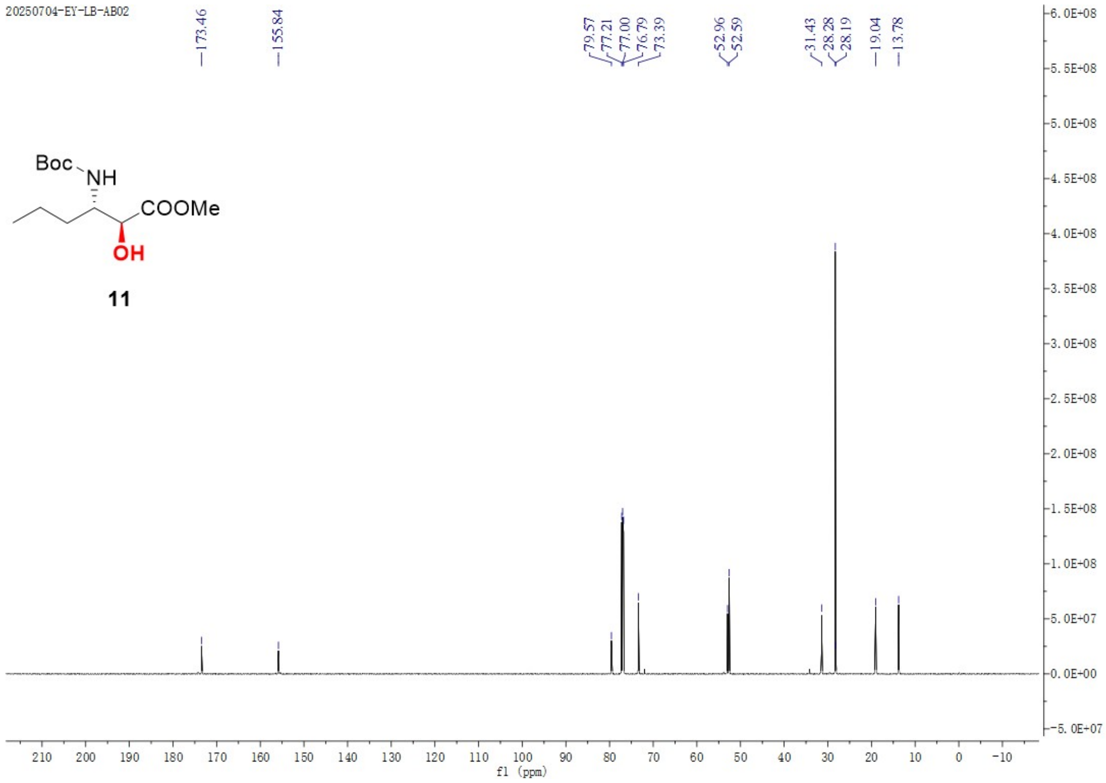

Figure S54. ^{13}C NMR of **11** ($CDCl_3$, 151 MHz)

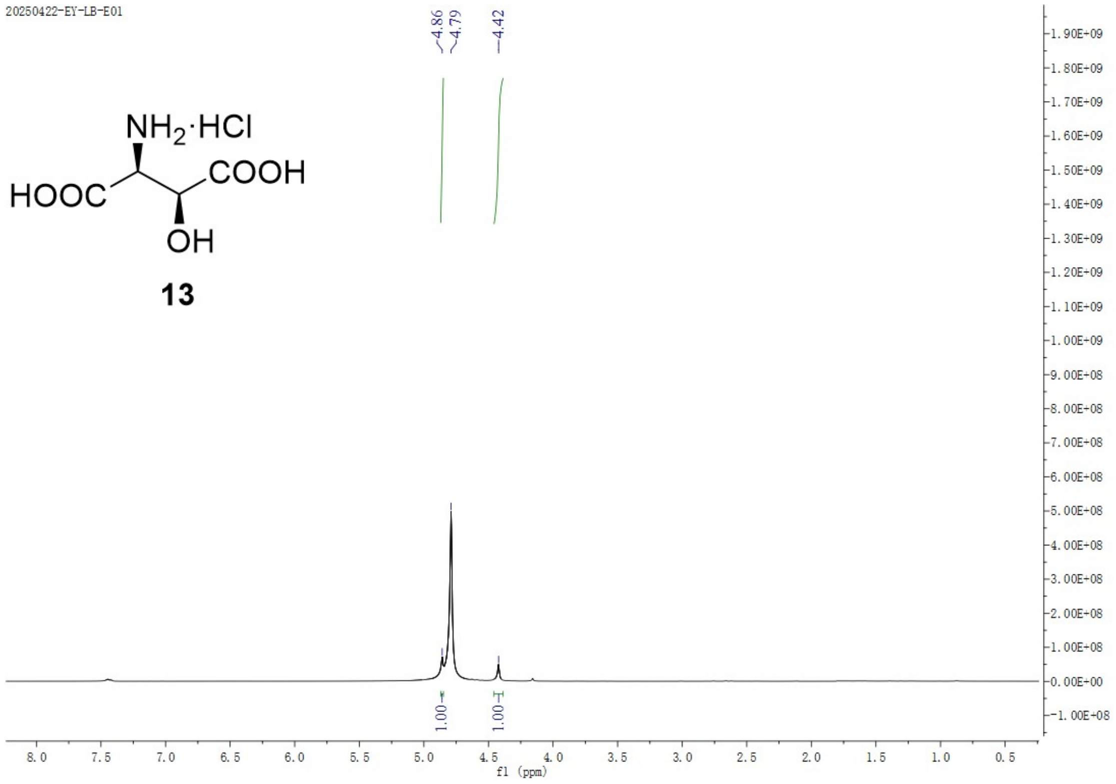

Figure S55. ^{1}H NMR of **13** (D_2O, 600 MHz)

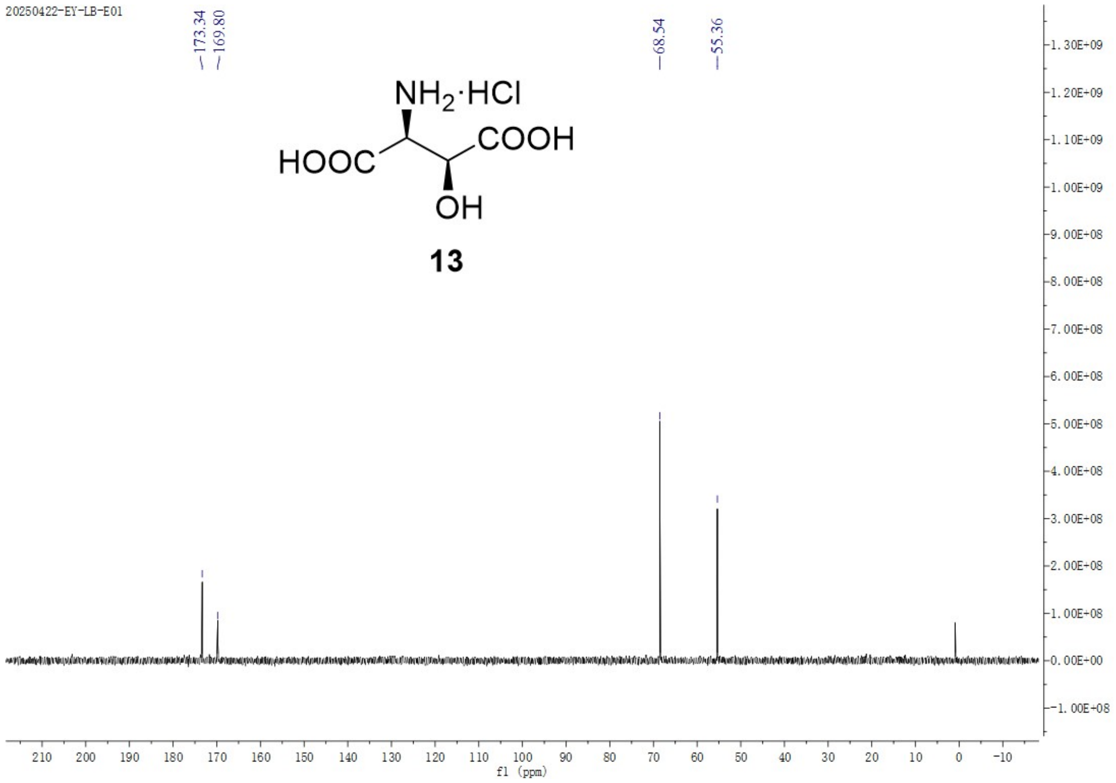

Figure S56. ^{13}C NMR of **13** (D_2O, 151 MHz)

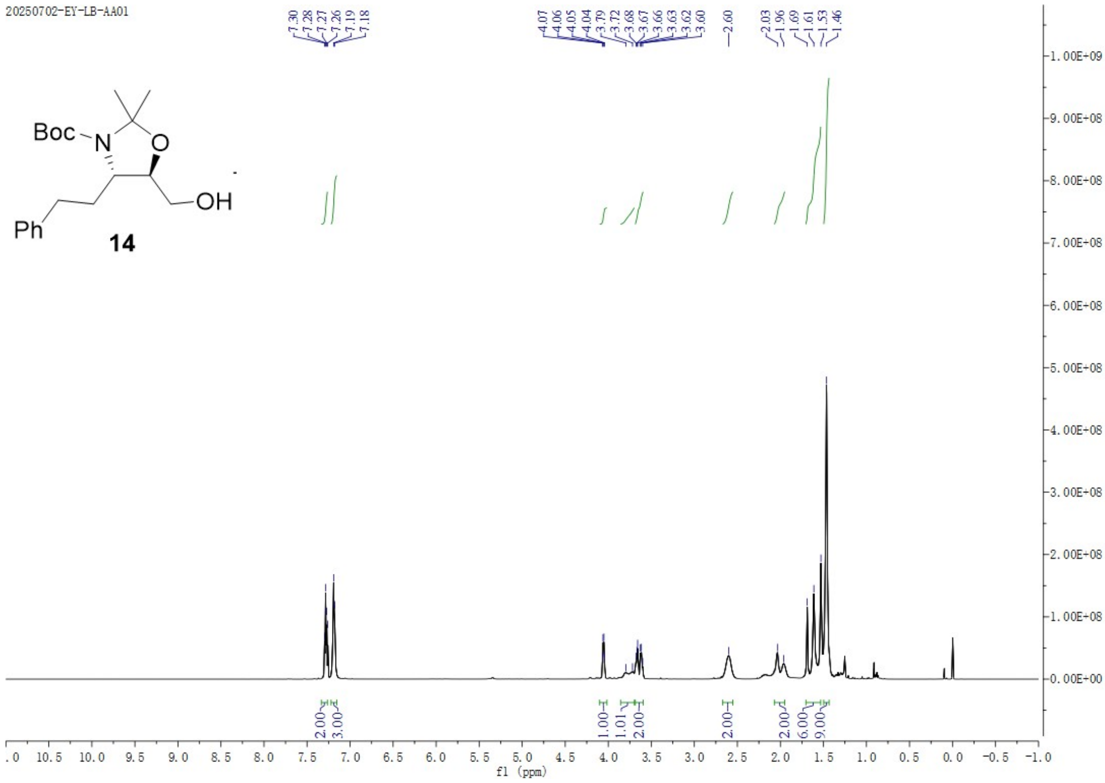

Figure S57. ^{1}H NMR of **14** ($CDCl_3$, 600 MHz) (*exists as rotamers*)

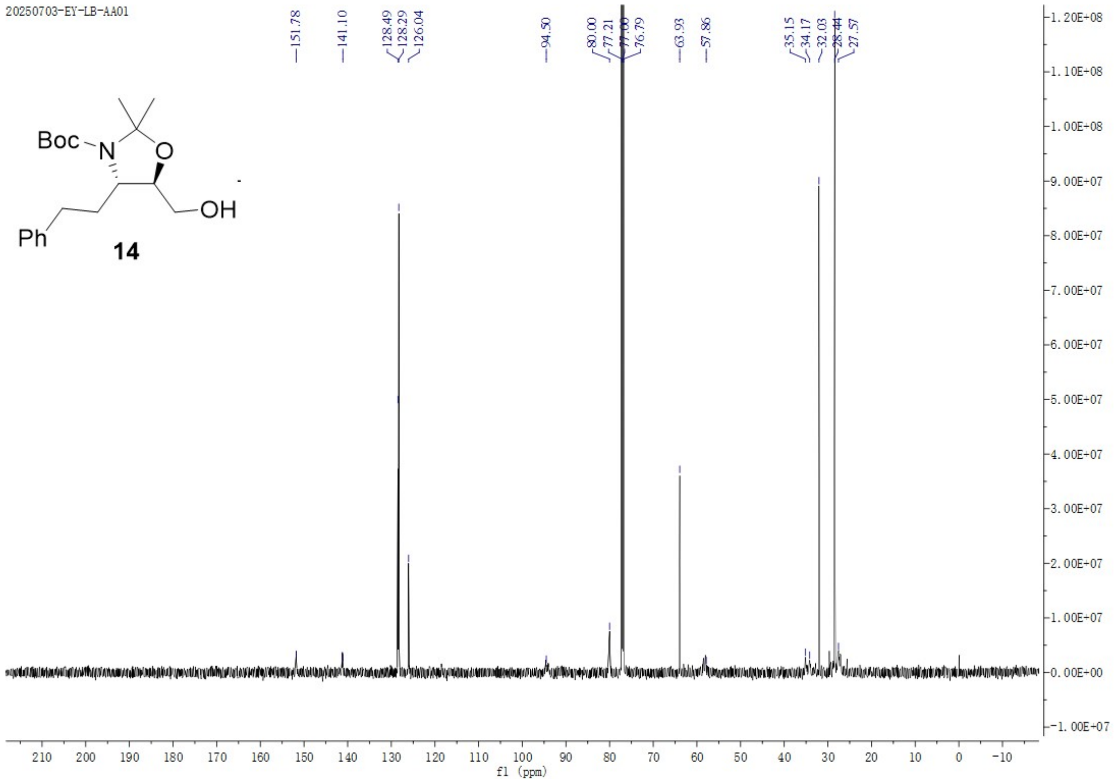

Figure S58. ^{13}C NMR of **14** ($CDCl_3$, 151 MHz) (*exists as rotamers*)

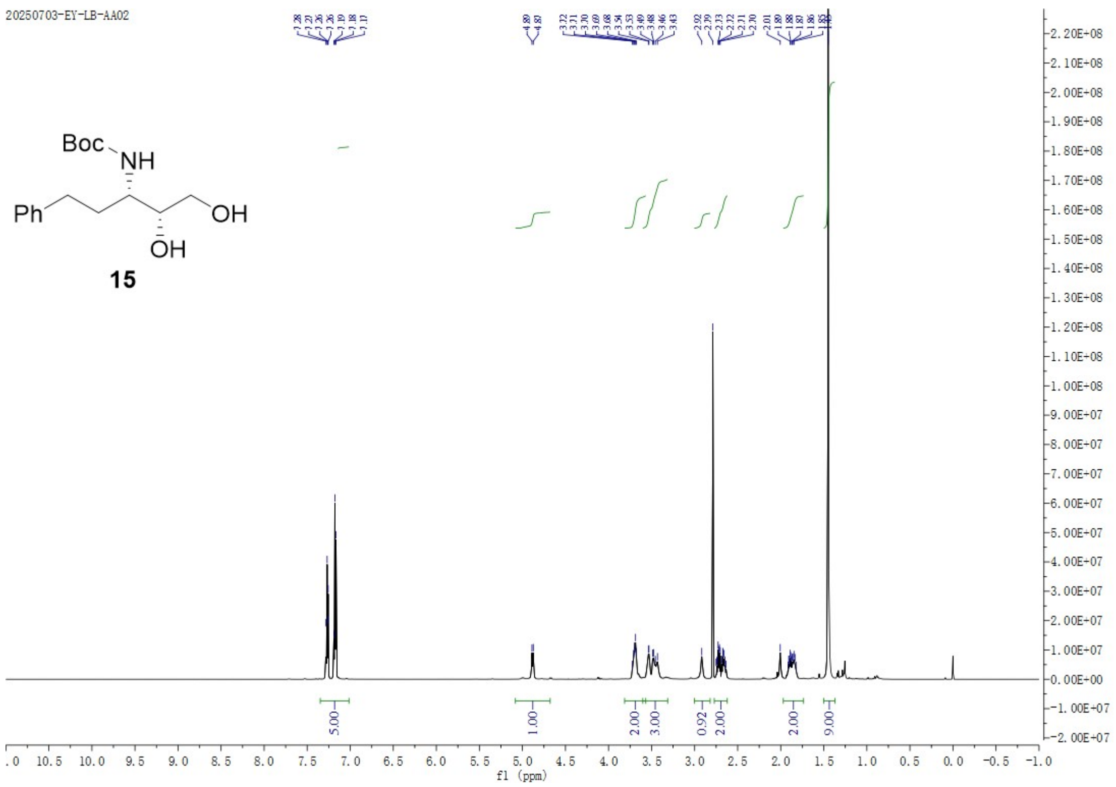

Figure S59. ^{1}H NMR of **15** ($CDCl_3$, 600 MHz)

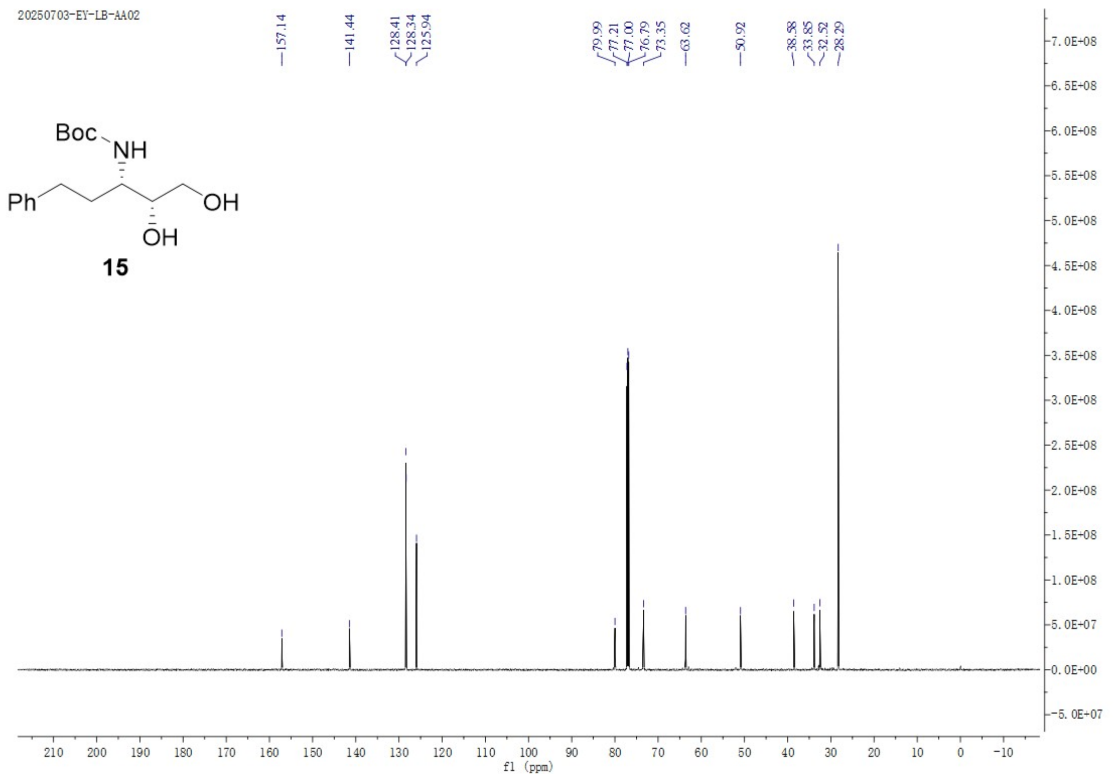

Figure S60. ^{13}C NMR of **15** ($CDCl_3$, 151 MHz)
